# GPCR Genes Are Preferentially Retained after Whole Genome Duplication

**DOI:** 10.1371/journal.pone.0001903

**Published:** 2008-04-02

**Authors:** Jenia Semyonov, Jae-Il Park, Chia Lin Chang, Sheau Yu Teddy Hsu

**Affiliations:** 1 Division of Reproductive Biology, Department of Obstetrics and Gynecology, Stanford University School of Medicine, Stanford, California, United States of America; 2 Chang Gung University School of Medicine, and Department of Obstetrics and Gynecology, Chang Gung Memorial Hospital, Tao-Yuan, Taiwan; Institut Pasteur, France

## Abstract

One of the most interesting questions in biology is whether certain pathways have been favored during evolution, and if so, what properties could cause such a preference. Due to the lack of experimental evidence, whether select gene families have been preferentially retained over time after duplication in metazoan organisms remains unclear. Here, by syntenic mapping of nonchemosensory G protein-coupled receptor genes (nGPCRs which represent half the receptome for transmembrane signaling) in the vertebrate genomes, we found that, as opposed to the 8–15% retention rate for whole genome duplication (WGD)-derived gene duplicates in the entire genome of pufferfish, greater than 27.8% of WGD-derived nGPCRs which interact with a nonpeptide ligand were retained after WGD in pufferfish *Tetraodon nigroviridis*. In addition, we show that concurrent duplication of cognate ligand genes by WGD could impose selection of nGPCRs that interact with a polypeptide ligand. Against less than 2.25% probability for parallel retention of a pair of WGD-derived ligands and a pair of cognate receptor duplicates, we found a more than 8.9% retention of WGD-derived ligand-nGPCR pairs–threefold greater than one would surmise. These results demonstrate that gene retention is not uniform after WGD in vertebrates, and suggest a Darwinian selection of GPCR-mediated intercellular communication in metazoan organisms.

## Introduction

Studies of the evolutionary paths of genes have shown that genome novelty is generated primarily by gene duplication and subsequent functional changes, and to a lesser extent, by *de novo* generation or the creation of mosaic genes [1], [2], [3]. Gene duplication not only provides more substrates for divergence through subfunctionalization or neofunctionalization, but also establishes a robustness against null phenotypes through compensation [2], [4], [5], [6], [7], [8], [9], [10], [11]. Recently, it was shown that gene duplicability may be associated with gene and protein complexity [12], [13], [14]; however, from these earlier studies one cannot discern whether the fixation of gene duplicate(s) is due to incidences of increased duplication or preferential retention. Consequently, no consensus theory has been presented on whether specific families of genes are preferentially retained following gene duplication at either local, segmental, chromosomal, or genomic level.

To investigate whether the rate of retention for select genes after duplication, not gene duplicability which is the sum of results from gene duplication and gene retention, is greater when compared to the genome average, we explored the recently available syntenic maps of representative tetrapods and teleosts that experienced a lineage-specific whole genome duplication (WGD) more than 230 million years ago. Given an equal opportunity for duplication for all genes during WGD, one could quantitatively analyze the relationship between gene retention and gene function by comparing the inventory of orthologous genes in nonduplicated species (tetrapods) with that from lineages experiencing WGD (teleosts). As all genes duplicate in parallel during WGD, these analyses would avoid errors associated with heterogeneity in gene divergence (heterotachy) [15], [16]. With this understanding, we reasoned that if WGD-derived duplicates belonging to a select group of genes are present in greater proportion when compared to the average of the entire genome, the data would support the hypothesis that select gene families are predisposed for retention after gene duplication.

Major advances during metazoan evolution include overall divergence in cell types associated with specialized functions and the expansion of intercellular signaling networks. As cell types increase, the need for selective intercellular communication increases. As opposed to the single cell yeast that encodes only a primitive mating signaling system, vertebrates have a multitudinous selection of specialized intercellular signaling pathways for communicating among >250 different cell types. Our earlier studies have shown that different classes of cell surface receptors emerged and expanded at discrete evolutionary times. Whereas some cell surface receptor families are vertebrate-, chordate-, or urbilateria-specific, the seven-transmembrane (7TM) receptors are present in all eukaryotes [17], and the proportion of 7TM receptor genes increases from 0.05% in unicellular yeast to more than 3% in multiple metazoan lineages [18], [19], [20]. Although the mechanisms underlying the expansion of cell surface receptors in metazoans are not clear, we hypothesized that the fitness associated with, 1) the potential to increase signaling specificity, and 2) the unidirectional signaling characteristics of cell surface receptors could impose a lower genetic constraint on the retention of receptor genes after duplication, thereby setting them apart from intracellular proteins that normally interact with a multitude of partners in two-way communication.

To test this hypothesis, we analyzed the retention of WGD-derived duplicates of nonchemosensory G protein-coupled receptors (nGPCRs), which together represent a majority of the receptome in vertebrates, as well as their cognate ligands in pufferfish [17], [18]. Among the protein families, the structurally constrained nGPCRs represent one of the few groups of genes that meets our requirements for gene retention studies–descendent genes must retain features of their predecessors significant enough to allow the tracing of orthologous relationships, and have similar gene ontology in molecular function, biological processes, and cellular components [21]. For the quantitative analysis of gene retention, these requirements are essential to reduce the bias associated with heterotachy, gene shuffling, and chimerization [15], [16]. In agreement with our hypothesis, our study demonstrates that gene retention is not uniform after WGD, and suggests a Darwinian selection of GPCR-mediated signaling for intercellular signaling in metazoan organisms.

## Results and Discussion

### Ancestral nGPCR genes gave rise to twice as many descendents in teleosts as in tetrapods

Earlier analyses of genomes showed that humans and mice share a similar repertoire of nGPCRs and encode about 367 and 392 nGPCRs, respectively, belonging to rhodopsin (class A), secretin receptor (class B), GABA receptor (class C), and Frizzled receptor (class F) classes [19], [22], [23]. In addition to these nGPCRs, vertebrates encode several groups of chemosensory GPCRs including olfactory receptors, vomeronasal receptor-like genes, taste receptors, and pheromone receptors [18], [20], [24], [25], [26], [27], [28], [29], [30], [31]. Because selection pressure has driven significant lineage-specific expansions of these chemosensory receptors, the inventory of these 7TM receptor genes varies drastically even among closely related species, thereby precluding them from the assignment of orthologous relationships among species [32]. Thus, we focused our analyses on nGPCRs.

**Figure 1:**
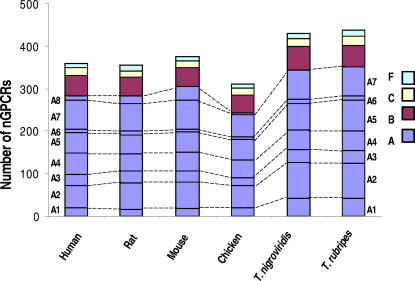
Inventory of nonchemosensory GPCRs (nGPCRs) in vertebrates. Nonchemosensory GPCRs belonging to the rhodopsin class (A), the secretin class (B), the metabotropic glutamate class (C), and the Frizzled class (F) were identified from the genomes of human (N = 359), rat (N = 359), mouse (N = 382), chicken (N = 310), pufferfish *Tetraodon nigroviridis* (N = 431), and pufferfish *Takifugu rubripes* (N = 438)(see Tables S1 A and S1 B for a complete list). Receptors belonging to different classes are indicated by different colors in the stacked bars. The rhodopsin class nGPCRs are subdivided into eight subclasses (A1–A8) based on their phylogenetic relationships and the chemical properties of the ligand [19].

In our searches using the published inventory of human and mouse nGPCRs as queries, we found that human, rat, mouse, chicken, *T. rubripes*, and *T. nigroviridis* each contain approximately 359, 359, 382, 310, 431, and 438 genes, respectively, belonging to the four main nGPCR classes (A, B, C, and F) (Fig. 1, Tables S1 A and S1 B) [18], [19], [22], [23]. In addition, we subdivided the largest class, class A nGPCRs, into eight subclasses (A1–A8) based on phylogenetic relatedness and the chemical properties of the cognate ligand(s) in order to facilitate our subsequent analyses of the relationships between gene retention and receptor characteristics [19]: The majority of nGPCRs in the A1–A4 subclasses interact with a ligand not encoded by a gene, including photons, ions, derivatives of lipids, carbohydrates, amino acids, and nucleosides, whereas those in other subclasses mainly interact with polypeptide ligand(s) (Fig. 1, Tables S1 A and S1 B).

To obtain a basal point for tracing the evolutionary changes of orthologous nGPCRs in duplicated (teleost) and nonduplicated (tetrapod) genomes, we first defined the nGPCR inventory in the most recent common ancestor (MRCA) of these species. Through phylogenetic analysis and syntenic mapping, we identified a total of 269 clusters of orthologous nGPCRs (207 class A, 38 class B, 14 class C, and 10 class F) activated by a variety of neurotransmitters, nucleoside derivatives, lipophilic compounds, or peptide hormones (Tables S2 A and S2 B). The major exceptions are approximately two dozen mammal- or tetrapod-specific tandem duplication-derived trace amine receptors (subclass A2), chemokine receptors (subclass A7), MAS-related family receptors (subclass A8), and the origins of these nGPCRs remain to be determined (Table S2 C) [33], [34], [35]. Based on these analyses, we inferred that the MRCAs of tetrapods and teleosts contained at least 269 ancestral nGPCRs belonging to A1–A7, B, C, and F classes over 450 million years ago.

**Figure 2:**
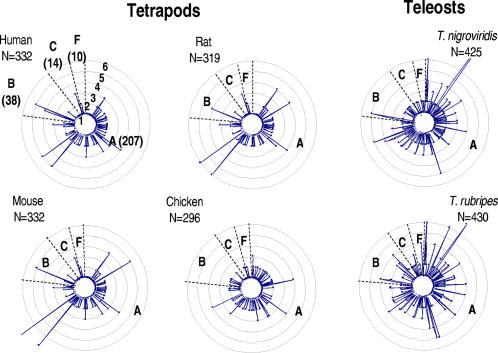
Radial evolution of nGPCR genes in tetrapods and teleosts. Based on phylogenetic analyses of nGPCR homologs from different vertebrate species, we deduced that the genome of the most recent common ancestor (MRCA) of tetrapods and teleosts encoded at least 269 ancestral nGPCR genes belonging to the four main classes (207 class A, 38 class B, 14 class C, and 10 class F) of vertebrate nGPCRs. A) The radar plot shows the number of paralogous nGPCRs derived from each of the 269 nGPCR ancestors in human, rat, mouse, chicken, *T. nigroviridis*, and *T. rubripes* (see complete list in Tables S2 A and S2 B). Each ring of the radar plot represents one copy of a paralogous gene, and the divisions of different classes of nGPCRs are indicated by dashed lines. The presence of duplicates is most widespread in *T. nigroviridis* and *T. rubripes*. B) Approximately 65% of pufferfish nGPCRs are derived from lineage-specific gene duplications whereas only 32.3–37.5% of nGPCRs in tetrapods evolved from gene duplications after the separation of tetrapods and teleosts (lower panel). Analyses based on a per ancestral gene basis showed more than 41% of MRCA nGPCRs evolved into more than one paralog in teleosts as compared to only 16.3–21.3% in tetrapods (upper panel).

Radar-plots of descendent genes derived from each of the 269 ancestral nGPCRs showed that the number of descendent genes varies from 0 to more than 6 in these species (Fig. 2A). As expected, the number of descendent genes derived from the 269 nGPCR ancestors is highly correlated between the two pufferfish (R^2^ = 0.79) or tetrapod species (e.g., R^2^ = 0.91 between human and mouse)(Table S2 D). However, the correlations between that of teleost and tetrapod species are significantly lower (e.g., R^2^ = 0.12 for *T. nigroviridis* and human, Table S2 D). These analyses further indicated that more than 41% of nGPCR ancestors evolved into more than one descendent gene in *T. nigroviridis* and *T. rubripes* (Fig. 2B, upper panel; Table S2 E), whereas only 16.3–21.3% of nGPCR ancestors gave rise to more than one nGPCR paralog in tetrapods. In pufferfish, these descendent genes represent approximately 65% of the nGPCR repertoire (276/425 in *T. nigroviridis* and 282/430 in *T. rubripes*)(Fig. 2B, lower panel; Table S2 E). In contrast, only 32.2–37.5% of the nGPCR inventory in tetrapods evolved from gene duplication after the separation from teleosts. The combined results thus suggested that a large fraction of nGPCRs in tetrapods and teleosts evolved independently after the separation of these two lineages.

Although vertebrates from pufferfish to humans share a similar gene inventory, recent analyses demonstrated that a WGD occurred before the divergence of teleosts and osteoglossomorphs more than 230–350 million years ago, whereas other ray-finned fish (actinopterygians) and all sarcopterygians (tetrapods and coelacanthiforms) experienced no such event [36], [37], [38], [39], [40], [41], [42], [43], [44], [45], [46], [47]. As tetraploidy was deleterious and strongly selected against, the duplicated genomes in the tetraploid teleost ancestor eventually coalesced in a process called diploidization [3], [48], [49], [50], [51]. Based on a spectrum of analytical approaches and stringency settings in defining WGD-derived gene duplicates, several recent studies have estimated that only 8 to 15% of WGD-derived duplicates were retained in present-day pufferfish, *Takifugu rubripes* and *Tetraodon nigroviridis* [36], [37], [43], [45], [47], [52]. The finding that over 65% of the pufferfish nGPCR repertoire consists of lineage-specific duplicates was unexpected as evidence has shown that, 1) vertebrates from teleosts to tetrapods share a similar gene inventory, and 2) only 8–15% of WGD-derived gene duplicates survive in pufferfish [36], [37], [43], [45], [47], [52]. These data implied that evolution of nGPCRs as a category could have been effected by a selection pressure different from that for the rest of the genome after the instance of WGD in teleosts.

### WGD-derived nGPCR duplicates were retained at a rate significantly higher than that for the entire genome

**Figure 3:**
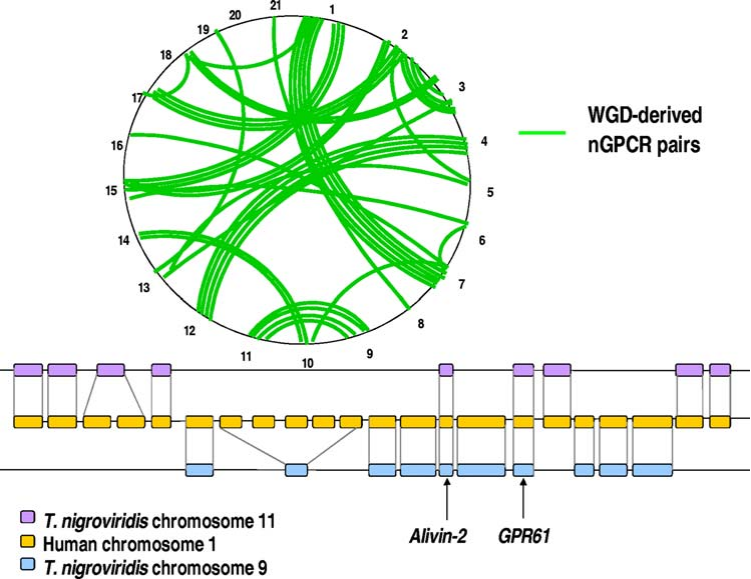
Analysis of gene retention following whole genome duplication (WGD) in *Tetraodon nigroviridis*. A) Global distribution of 39 pairs of WGD-derived nGPCR duplicates on *T. nigroviridis* chromosomes (upper panel). Pairs of duplicates on two syntenic chromosome regions are indicated by green lines, similar to the distribution of WGD-derived syntenic regions mapped previously [36]. Schematic representation of the WGD-derived *GPR61* duplicates on chromosomes 1 and 9 of *T. nigroviridis* (lower panel). The duplicates are flanked by different sets of genes found on the syntenic region of human chromosome 1 in a disequilibrate manner. Another pair of WGD-derived genes in the nearby regions, Alivin-2, also is indicated. B) The retention rate of WGD-derived nGPCRs duplicates (23.5%) is significantly higher than a high estimate (15%) for the entire genome (t-test, P = 0.00014)[36], [37], [43], [45], [47], [52]. To compare differences in gene retention rate, each nGPCR family was assigned with a fixed value, 0 for families with a singleton in *T. nigroviridis*, and 1 for those with WGD-derived duplicates in *T. nigroviridis*. *, significantly different from the expected value.

Given the finding that teleosts experienced a lineage-specific WGD during evolution, we hypothesized that the large nGPCR inventory in teleosts could be attributed to, 1) increases in the incidence of tandem duplication-derived nGPCRs, and/or 2) increases in the retention of WGD-derived nGPCR duplicates. Analysis of the distribution of the166 nGPCR families with assigned chromosomal localization(s) on *T. nigroviridis* chromosomes showed that 17 pairs of these nGPCRs represent paralogs derived from tandem duplications (Table S3 A). Likewise, we found that 18, 17, 18, and 12 groups of paralogous nGPCRs from human, rat, mouse, and chicken, respectively, were derived from intrachromosomal tandem duplications (Table S3 B), and that all tetrapods are endowed with seven groups of these duplicates (Table S3 C). Therefore, the rate of tandem duplication in teleosts and tetrapods is similar, and the small number of tandem duplication-derived duplicates cannot account for the large expansion of nGPCR homologs in pufferfish. In contrast, we found that 39 pairs of these nGPCRs were located on two WGD-derived syntenic chromosomal regions (or homologons), reminiscent of the binary distribution of the 750 pairs of previously characterized WGD-derived gene duplicates in *T. nigroviridis* (Fig. 3A, upper panel; an example of the binary distribution for WGD-derived *GPR61* duplicates is shown in the lower panel; Tables S3 D and S3 E)[36], [51]. The finding is important as data indicate that more than 23.5% (39/166 receptor families with assigned chromosomal localization(s)) of WGD-derived nGPCR genes were retained and fixed in pufferfish. This retention rate is significantly higher than the high retention rate estimate of 15% for the entire genome from studies using similar statistical criteria (Fig. 3B); t-test, P = 0.00014) [36], [37], [43], [45], [47], [52]. In support of the above findings, analysis of the *T. rubripes* genome showed that orthologs for at least 33 of the 39 pairs of WGD-derived nGPCR duplicates are present in *T. rubripes* in spite of a lack of chromosomal localization information in this species (Table S2 B). Because duplicated genes in general degenerate within a few million years [3], these data thus indicate that nGPCR gene duplicates have a greater probability of escaping gene loss after WGD than average genes in the genome.

### Preferential retention of nGPCRs that interact with ligands not encoded by a gene

Although no prior study has addressed the mechanisms for the preferential retention of genes, studies of genes from unicellular organisms and mammals suggested that gene duplicability could be associated with gene complexity and protein length [12], [13], [14]. To investigate whether the preferential retention of nGPCRs following WGD could be associated with select molecular attributes of nGPCRs, we analyzed the relationships between retention rate and, 1) receptor length, 2) chemical properties of the cognate ligand, and 3) molecular weights (MWs) of ligands. Findings of a significant correlation between retention rate and one of these traits would not only further support the existence of preferentiality in gene retention, but also reveal the underlying mechanisms. Because data on the open reading frame (ORF) of human nGPCRs and their cognate ligands are more complete as compared to those of pufferfish, we used human counterparts as proxies for the analysis of receptor length and ligand size. First, our comparisons of the receptor length of nGPCRs showed that the average receptor length for all nGPCRs is 551±32 amino acids (Log_2_ ORF = 8.89±0.04, N = 269; Table S4 A), and that there is a negligible difference in receptor length between nGPCRs with WGD-derived duplicates in *T. nigroviridis* (Log_2_ ORF = 8.92±0.10, N = 39) and those with a singleton (Log_2_ ORF = 8.91±0.06, N = 153). Thus, the increased retention of WGD-derived nGPCR duplicates is not associated with the length, or protein complexity, of the receptors.

**Figure 4:**
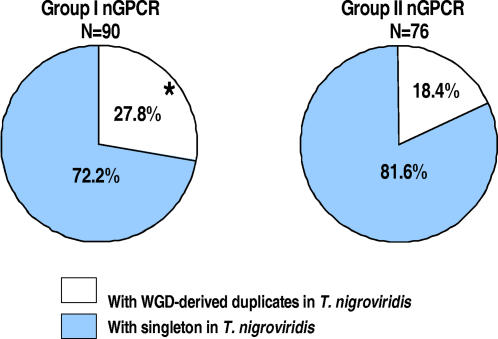
Preferential retention of nGPCR duplicates with a nonpeptide ligand after WGD in teleosts. A) The retention rate of WGD-derived duplicates for nGPCRs with a nonpeptide ligand(s) (Group I, 27.8%), but not nGPCRs with a polypeptide ligand (Group II, 18.4%), is significantly higher than the estimate for the entire genome (t-test, P = 0.00068). The Group I receptors include nGPCRs of subclasses A1-A4 and class C whereas the Group II receptors include those belonging to subclasses A5–A7, class B, and class F. To compare differences in gene retention rate, each nGPCR family was assigned with a fixed value, 0 for families with a singleton in *T. nigroviridis*, and 1 for those with WGD-derived duplicates in *T. nigroviridis*. *, significantly different from the expected value 15%. B) The average MW (mean±SEM, Log_10_ transformed) of the cognate ligands for nGPCRs with WGD-derived duplicates in *T. nigroviridis* (2.44±0.25) is significantly less than those of singleton (3.01±0.09) or the entire pool of nGPCRs (2.92±0.07). For statistical analysis, the MW of photons was arbitrarily set as one. *, significantly different from that of singleton nGPCRs.

Second, we analyzed the relationship between retention rate and the chemical properties of the cognate ligand. Earlier studies have shown that there is a strong correlation between the phylogeny of nGPCRs and the chemical properties of their cognate ligands–nGPCRs with close relatedness tend to interact with ligand(s) of similar chemical properties [19]. To factor these two associated parameters (receptor phylogeny and ligand properties) into our analysis, we divided the nGPCRs into two separate groups: Group I included subclasses A1–A4 and class C receptors, the majority of which interact or potentially interact with a nonpeptide ligand (e.g., photon, ions, monoamine derivatives, lipophilic compounds, and nucleoside derivatives), and Group II included subclasses A5–A7, class B, and class F receptors, which primarily interact with a gene-encoded polypeptide(s). We found that Group I nGPCRs (27.8%; 25/90 families) have a significantly higher retention rate than the genome average (Fig. 4A; t-test, P = 0.00068) whereas the Group II receptors (18.4%; 14/76 families) did not. These data thus suggest that the preferential retention of nGPCRs as a group is a result of greater retention of Group I nGPCRs that interact with a nonpeptide ligand. This distinction in the retention rate of Group I and Group II nGPCRs is further reflected in analyses of ligand size and retention rate. Comparison of the MWs of ligands for all nGPCRs with a known ligand showed that the MWs of ligands for singleton nGPCRs (Log_10_MW = 3.01±0.09, N = 104; Fig. 4B; Table S4 B) are 60% greater than that of nGPCRs with WGD-derived duplicates in *T. nigroviridis* (Log_10_MW = 2.44±0.25, N = 26; P<0.05), consistent with the finding that interaction with a small nonpeptide ligand could provide an opportunity for retention. Because duplicated nGPCRs with a nonpeptide ligand presumably could undergo sub-functionalization or neo-functionalization without concurrent genetic changes in their major interacting partner–a property not shared by the majority of genes–by default, they would have a greater chance of escaping random gene loss before acquiring a beneficial mutation.

### Retention of WGD-derived polypeptide nGPCRs is facilitated by the co-evolution of WGD-derived ligand genes

Aside from the proposition that preferentiality in nGPCR retention is associated with the chemical properties of a ligand, we investigated whether the retention of WGD-derived nGPCR duplicates could be effected by other selection forces because select subcategories of nGPCRs that interact with a polypeptide ligand (polypeptide nGPCRs) also exhibit a higher retention rate when compared to the average for the genome (e.g., 33.3% of class B nGPCR families with assigned chromosome localizations contain WGD-derived duplicates in *T. nigroviridis*). Because an extra set of cognate ligands that interact with polypeptide nGPCRs was also generated during WGD, the parallel duplication of ligand-receptor pairs potentially could provide opportunities for the evolution of novel signaling pathways and facilitate the retention of the duplicated ligand-receptor pairs. Therefore, if the pufferfish genome retained a higher number of WGD-derived cognate ligand-nGPCR pairs as compared to the estimate derived from whole genome analysis, the data would further support our hypothesis that nGPCR signaling pathways are favored for retention after WGD. To investigate this possibility, we searched and analyzed the chromosomal distribution of genes encoding high affinity polypeptide ligands of nGPCRs belonging to subclasses A5–A7 and class B in humans and *T. nigroviridis*. In this analysis, the ligands for class F nGPCRs were excluded because they are promiscuous in receptor interactions, and no clear cognate ligand-receptor pairs can be defined.

With the same approach that we used to identify nGPCR homologs and novel peptide hormones in earlier studies [53], [54], [55], [56], [57], [58], we identified 118 human and 118 *T. nigroviridis* ligand genes that encoded polypeptide ligands for the 81 families of nGPCRs known to interact with a polypeptide ligand (Tables S5 A and S5 B). Based on syntenic mapping and sequence comparison, we inferred that these ligand genes were derived from 76 ancestral genes in the MRCA of tetrapods and teleosts, and that 17.3% of these ligand gene families (9 pairs out of 52 families of ligand genes with assigned chromosome localizations) in *T. nigroviridis* contained WGD-derived duplicates, a level of gene retention similar to that of the genome average (Table S5 C).

Based on the 15% estimate for gene retention in the entire genome, there is a 2.25% probability for parallel retention of any given pair of WGD-derived ligands and their WGD-derived cognate receptors assuming they evolved independently. Against this low probability, we found that in *T. nigroviridis* over 9.6% of WGD-derived ligand genes (5/52 families of ligand genes with assigned chromosome localizations; *NMB*, *RLN3*, *INSL5*, *CALCA*, and *ADM*) coevolved with four pairs of WGD-derived cognate receptor duplicates (8.9%; 4/45 families of polypeptide nGPCRs with assigned chromosome localizations; *GRPR*, *RLN3R1*, *RLN3R2*, and *CLR*), a rate three to fourfold that of random probability (Table S5 C; the binary distribution for WGD-derived *CALCA* and *ADM* duplicates on syntenic chromosome fragments is shown in Fig. S1). Importantly, these data also showed that 55.6% (5/9) of ligand families and 44.4% (4/9) of polypeptide nGPCR families with WGD-derived duplicates in *T. nigroviridis* coevolved with a pair of WGD-derived partners. In retrospect, the 8.9∼9.6% retention rate of WGD-derived cognate ligand-receptor pairs observed would require a 29.8∼31% retention rate for all WGD-derived genes in the entire genome, a high level not compatible with any previous study [36], [37], [52]. Therefore, the most parsimonious evolutionary course for the observation is that parallel duplication of polypeptide nGPCRs and their cognate ligand genes by WGD was crucial to allow the retention of WGD-derived ligand-receptor pairs. Of importance, these data also further support the hypothesis that nonpeptide nGPCR duplicates were preferentially retained after WGD as a result of low genetic constraint. Whereas the underlying mechanisms for the co-retention of WGD-derived ligand-receptor pairs remain to be investigated, these WGD-derived ligand-nGPCR pairs could evolve in a manner similar to the “divergent resolution” model that was proposed to illustrate the separation of different copies of a duplicated gene in allopatric populations during sympatric evolution [59]. In this scenario, fitness associated with increased signal-to-noise ratio of the two diverging WGD-derived co-orthologus ligand-receptor pairs in individuals was selected, similar to the retention of a different copy of duplicated genes in reproductively separated populations [59].

### WGD-derived nGPCR duplicates underwent drastic divergence in the functional domain

**Figure 5:**
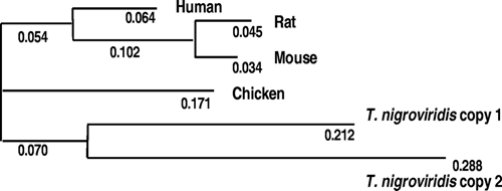
Sequence divergence of WGD-derived nGPCR duplicates in *T. nigroviridis*. Global distance tree based on concatenated sequences of the 39 families of nGPCRs with WGD-derived duplicates in *T. nigroviridis.* Distances are indicated next to individual branches. The tree was calculated using a Gonnet 250 matrix. Each pair of WGD-derived duplicates was subdivided into two subgroups (the conserved copy 1 and the divergent copy 2) and analyzed separately. For nGPCR families with more than one ortholog in tetrapods, one was chosen randomly for analysis.

In addition to the above, we have observed that WGD-derived nGPCR duplicates generally exhibit a low degree of sequence similarity to each other, suggesting a trend of asymmetric divergence in these co-orthologs. To investigate whether nGPCR duplicates exhibit an accelerated divergence that could serve new functions, we compared the sequence divergence of the two WGD-derived duplicates. On average, pufferfish nGPCRs share 71.1–71.8% sequence similarity with human orthologs (Fig. S2). These estimates are similar to those of the entire proteomes among these species; therefore, nGPCRs as a group evolved at a pace similar to that of the rest of the proteome [36]. However, a distance tree calculated from the concatenated sequences of the 39 families of nGPCRs with WGD-derived duplicates in *T. nigroviridis* showed that the two WGD-derived co-orthologs are farther from each other as compared to the distance to human orthologs (Fig. 5). These results are reminiscent of that reported for WGD-derived gene duplicates in yeast [51], and suggest that the WGD-derived nGPCR duplicates evolved via sub-neofunctionalization in which one copy of duplicates would undergo positive selection and evolve faster than the other [51].

### GPCR signaling is a favored evolutionary path

By analyzing the fate of orthologous genes of nGPCRs and their cognate ligands in vertebrates, we demonstrated that nGPCR signaling has been a favored evolutionary path in a natural experiment conducted over the past 230 million years. Importantly, our studies satisfy several requirements for demonstrating the preferential retention of genes, rather than an increased gene duplicability, during evolution. In addition to the revelation that given an equal opportunity for duplication, a higher probability for retention could be realized, at least in the realm of nGPCRs, we showed that this greater probability could be due to interaction with ligands not encoded by a gene.

**Figure 6:**
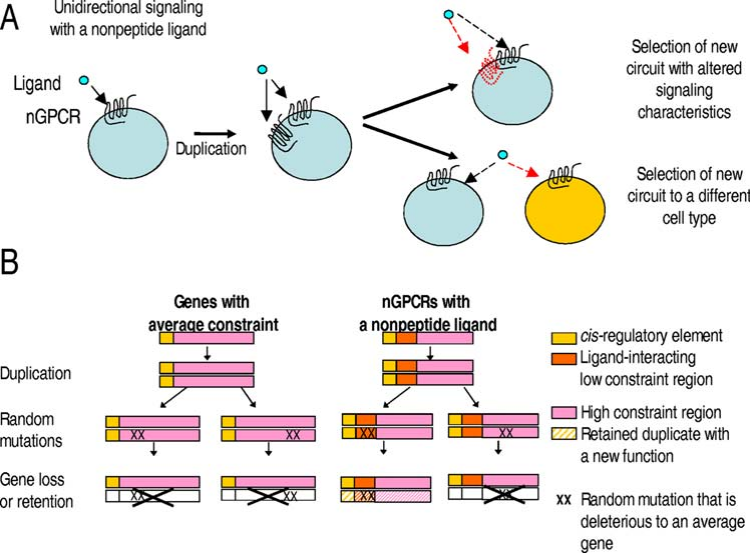
Putative mechanisms for the preferential retention of nGPCR genes after WGD. A) As compared to intracellular proteins that usually form complexes with many partners bi-directionally, a nonpeptide nGPCR, with part of its functional motifs dedicated to unidirectional interactions with an extracellular ligand, likely has a lower constraint on the divergence of the ligand-interaction domain [12], [13]. Mutations of the transactivation or the functional domain could subsequently lead to the generation of novel signaling circuit to the same cell population but with different pharmacological characteristics (indicated by a red dashed line) or new signaling pathway to a different cell population (indicated by a cell with yellow color). B) Schematic representation of the fitness associated with nGPCRs that interact with nonpeptide ligands during natural selection. In the event of gene duplication, the low constraint derived from interacting with a nonpeptide ligand would provide nGPCR duplicates with a higher probability for acquiring novelty and becoming fixed in the descendent genome, as compared to genes with average constraint. A beneficial mutation could occur in either the *cis*-regulatory element or functional domain (indicated by a schematic pattern change). An X sign across the gene indicates gene loss. An xx sign indicates deleterious mutations for an average gene.

Based on these findings, we speculate that a lower genetic constraint associated with a nonpeptide ligand, together with the unidirectional signaling characteristics, could allow the duplicated nGPCRs to survive a longer period of selection before acquiring beneficial mutations as compared to an intracellular polypeptide which normally forms complexes with many partners in two-way communication (Fig. 6A). Mutations of either the transactivation or the functional domain could then lead to the generation of novel unidirectional intercellular signaling circuits among cells; the new circuits include signaling to the same cell population but with different pharmacological characteristics or to a different cell population (Fig. 6A). Genetically, a lower constraint associated with these characteristics could allow nGPCR genes to better tolerate deleterious random mutations and accumulate beneficial mutations, thus allowing nGPCR duplicates to be fixed with a higher probability as compared to genes with average constraint (Fig. 6B). This hypothesis is compatible with the concepts that, 1) gene duplicability in unicellular organisms increases when the number of subunits in a protein complex decreases [9], [12], 2) a major portion of young genes exhibiting positive selection as calculated by the Ka/Ks ratio are genes involved in transient intercellular interactions such as defense, gamete interaction, or immunity against exogenous agents [36], [60], [61], [62], 3) major lineage-specific duplicated genes in mammals are genes that function in immunity, chemosensory, and reproduction [32], [63], and 4) single-nucleotide polymorphisms are more often found in GPCR genes as compared with non-GPCR genes [64]. In addition, because the preferentiality in nGPCR retention encompasses nGPCRs that interact with a variety of ligands, our data would reject alternative hypotheses regarding the large inventory of WGD-derived nGPCRs in teleosts, such as it being a consequence of adaptation to specific environmental factors surrounding the time of WGD in the teleost ancestor, or the development of a particular physiological process that is specific to the evolution of teleosts. Furthermore, it is interesting to note that recent phylogenomics analyses indicated that protein families related to GPCR signaling pathways represent a major group of genes expanded before amniota and mammalian radiation, and that proteins involved in interaction with the environment (e.g., immune response and xenobiotic metabolism) expanded steadily through gene duplications at various points of vertebrate evolution [60]. Overall, the combined evidences support our hypothesis that nGPCR duplicates are preferentially retained after gene duplication and caution the inference of studies assuming different gene families were retained at a similar pace during evolution.

Nonetheless, inasmuch as the lower genetic constraint hypothesis applies, the preferential retention of nGPCRs could be effected by a combination of selection forces. In addition to gamete compatibility, it is well recognized that differences in cognition and sensory perception could represent a particularly strong force leading to reproductive isolation. The provision of novel cognition and sensory perception pathways mediated by nGPCRs after WGD may constitute a rich source for adapting to new niches by providing the ability to adjust sensing, foraging, courtship, and other behaviors, without changes in the fundamental architecture of the cellular components, thereby leading to an enhanced retention of duplicated nGPCR genes [62], [65]. By the same token, we speculate that the same selection force underlying the preferential retention of nGPCRs after WGD may be the common denominator in the repeated expansion of nGPCRs and chemosensory 7TM receptors in different metazoan lineages [18], [20], [26], [29].

Finally, our studies generally validate century-old comparative endocrinology studies indicating all vertebrates share a similar set of hormones and receptors for cognition, sensation, and humoral homeostasis maintenance. However, the revelation that more than 280 nGPCRs and over 25 polypeptide ligand genes in teleosts are lineage-specific paralogs indicates that nGPCR-mediated regulatory circuits in teleosts have evolved with a remodeled platform, and points to the presence of a robust intercellular signaling network involving hundreds of novel ligand-receptor signaling pathways not found in tetrapods [62].

## Materials and Methods

### Nomenclature

We used the GPCR classification proposed by Bockaert and Pin [23] and Vassilatis et al. [19]. Human nGPCRs were named according to the recommendation of the International Union of Pharmacology [22], and each family of orthologous receptors is denoted by the name of the human ortholog(s).

### Protein and genomic sequence data

Human and rodent nGPCR sequences were obtained from the HPMR database, http://receptome.stanford.edu/hpmr/home.asp [17], and the NCBI databases ftp://ftp.ncbi.nlm.nih.gov/genomes. Chicken genomic and protein sequences were downloaded from the NCBI ftp site, ftp://ftp.ncbi.nlm.nih.gov/genomes/Gallus_gallus/ [66]. The *T. rubripes* proteome and genome sequences were obtained from the JGI database, http://genome.jgi-psf.org [67]. The *T. nigroviridis* genomic and protein sequences were obtained from the Genoscope database, http://www.genoscope.cns.fr [36].

### Determination of orthologous and co-orthologous relationships

Orthologous genes belonging to an nGPCR family from different species were determined by a series of reciprocal pairwise sequence comparisons using the BLAST server [32], [68], [69] and syntenic mapping. Initially, human and mouse nGPCR sequences were compared against the proteomes of rat, chicken, *T. nigroviridis,* and *T. rubripes*. The top thirty nonredundant hits were collected. Unique protein sequences with E<0.0001 were analyzed with additional blast searches against the human nGPCR dataset to detect the best reciprocal hits. Sequences that contained erroneous components from a neighboring gene were trimmed manually to obtain a continuous nGPCR ORF. The best hits were then collected and verified by blast searches against a human chemosensory GPCR dataset to exclude orthologs for olfactory GPCRs, vomeronasal receptor-like genes, taste receptors, and pheromone receptors from further analysis. In addition, proteins with a 7TM domain but do not share a common root with classes A, B, C, or F nGPCRs were excluded from analysis. Because GPCRs belong to each of the above-mentioned GPCR groups exhibit a distinct sequence profile, nGPCRs of various vertebrate species can be identified unambiguously using this procedure [19], [22].

For human nGPCRs where orthologs were not found in the protein databases of other species, the nGPCRs were analyzed with blast searches against genome sequences using the TBLASTN. Similar to studies of proteomes, the thirty best genomic hits were collected. Unique genomic sequences with E<0.0001 were then verified by blast searches against the human GPCR dataset. Identities of the genes encoded by genomic hits were further verified by blast searches against the nr database in GenBank in order to exclude nonGPCR or chemosensory GPCR genes. Sequence similarity between orthologous or co-orthologous nGPCRs was generated by the NCBI bl2seq program on a local server using default settings without a filter [70].

### Phylogenetic reconstruction

Unlike olfactory and vomeronasal receptors which expanded repeatedly in select vertebrate lineages, most nGPCR families originated before the evolution of euteleostomi species and contain an orhtolog or a small number of paralogs in most vertebrates. Based on the best reciprocal hit approach, we determined that >60% of nGPCR families contain one ortholog in different tetrapods (Table S2 E). However, the evolutionary history of >60% families of teleost nGPCRs cannot be resolved with this approach. To determine the evolutionary relationship of orthologous nGPCRs in each nGPCR family or within a subgroup of nGPCR families as well as concatenated sequences, we used the the ClustalW multiple sequence alignment program version 1.82 (http://www.ebi.ac.uk/clustalw/#) [71]. The phylogenetic reconstruction was based on the Neighbor-Joining (NJ) method [72]. Phylograms were first built with a default parameter (DNA Gap Open Penalty = 15.0, DNA Gap Extension Penalty = 6.66, DNA Matrix = Identity, Protein Gap Open Penalty = 10.0, Protein Gap Extension Penalty = 0.2, Protein Matrix = Gonnet, Protein/DNA ENDGAP = −1, Protein/DNA GAPDIST = 4). For families with multiple paralogs in select species, additional trees were reconstructed using the BLOSUM30 and PAM models as well as the drawtree program of PHYLIP3.65 package (http://evolution.genetics.washington.edu/phylip/getme.html) [73]. If a sequence was found to be positioned outside a main branch consisting of a group of orthologs from teleosts to humans, the sequence was then analyzed together with the next closest related nGPCR groups in an iterated manner until a best fit family was identified. Each of these independent nGPCR families was considered to be derived from an independent ancestral nGPCR rooted in the MRCA of tetrapods and teleosts. These analyses showed that orthologs from most nGPCR families share on average >70% sequence similarity, and most trees share a topology similar to that of concatenated sequences as shown in Fig. 5.

However, preliminary phylogenetic reconstruction studies showed that select WGD-derived duplicates of pufferfish have a basal position relative to the other WGD-derived co-ortholog in the phylogenetic tree, suggesting that gene phylogenies are insufficient to resolve the evolution history of WGD-derived co-orthologs in these nGPCR families. Instead of attributing to a massive gene loss in multiple Classes of tetrapods, we reasoned that the most parsimonious inference would be that the WGD-derived co-orthologs underwent neo-functionalization or sub-functionalization, and that heterotachy incurred by functional divergence led to the aberrant tree topology [15]. Therefore, we sought to determine the phylogenetic relationship of all nGPCR families with syntenic mapping.

### Identification of WGD-derived and tandem duplication-derived nGPCRs

Chromosomal localization of tetrapod nGPCRs was obtained from the NCBI database. Syntenic maps were downloaded from the Genoscope database (http://www.genoscope.cns.fr/externe/English/Projets/Projet_C/data/synteny/TN_HS_SYNT)[36] and Ensembl's BioMart data mining tool (http://www.ensembl.org/multi/martview)[74], [75]. The exact locations for human and *T. nigroviridis* co-orthologs were also verified by BLAT searches using the UCSC Genome Bioinformatics webserver (http://genome.ucsc.edu/cgi-bin/hgBlat)[76]. We inferred that a pair of duplicates would be WGD-derived co-orthologs if they were located on human-*T. nigroviridis* syntenic chromosomal regions. In these analyses, locations of *T. nigroviridis* genes were identified first using the Genoscope map, and then verified with a recently refined map in the Ensemble database. In contrast, nGPCRs found on neighboring loci on the same chromosome were determined to be derived from tandem duplications. Therefore, the presence of an ancestor for a select group of nGPCRs in the MRCA was deduced from analyses combining phylogenetic trees, BLAST results, and syntenic mapping. Based on these analyses, a total of 269 clusters of orthologous nGPCRs, belonging to A1–A7, B, C, and F classes, were obtained (Table S2 A). However, we cannot exclude the possibility, albeit at a low probability, that some teleost homologs found on syntenic chromosomal regions were not WGD-derived co-orthologs.

### Identification of WGD-derived ligand genes

The cognate ligand genes for polypeptide nGPCRs in humans and *T. nigroviridis* were identified by BLAT searches using mature regions of human ligands as the query. Positive hits were then manually sorted. To validate the authenticity of a ligand gene from *T. nigroviridis,* we compared the target sequences to orthologous sequences from all model vertebrate organisms in GenBank. Only sequences that contained the characteristic sequence motifs of the mature region of a given ligand were considered as a positive ortholog [56], [58]. We determined that a pair of ligand duplicates would be WGD-derived co-orthologs in a manner similar to that described for the nGPCR duplicates. Likewise, phylogenies of ligand genes were analyzed similar to that described for nGPCRs. The major difference is the inclusion of only putative mature regions of ligands in these analyses because the prepro-regions of peptide ligands were known to evolve with minimal selection constraints and diverge greatly among closely related species.

### Receptor length and molecular weight of nGPCR ligands

The length of human nGPCR ORF and the molecular weight of the cognate ligand(s) for human nGPCRs were obtained from the NCBI database and the literature by manual searches. In cases with more than one cognate ligand for a given nGPCR, the most potent ligand was used for analysis.

### Statistical analyses

Statistical analyses including t-test and ANOVA were performed using a Prism software package (GraphPad Software, Inc., San Diego, CA). To compare differences in gene retention rate, nGPCR and ligand gene families with a singleton or WGD-derived duplicates in *T. nigroviridis* were assigned with a fixed value, 0 for families with a singleton, and 1 for those with WGD-derived duplicates. The expected rate for retention of WGD-derived duplicates in *T. nigroviridis* was set at a high estimate (15%) based on several previous studies [36], [37], [52].

## Supporting Information

Figure S1 Localization of WGD-derived adrenomedullin (ADM) and calcitonin/CGRP (CALCA) gene duplicates on syntenic regions of chromosomes 5 and 13 of T. nigroviridis.
(0.01 MB PDF)

Figure S2 Comparison of sequence similarity between human nGPCRs and orthologs or co-orthologs from rat, mouse, chicken, T. nigroviridis, and T. rubripes. Each data point represents the average of multiple data points belonging to brackets increased by a 5% step in sequence similarity as shown on the Y axis. The average sequence similarity and sequence identity (mean±SEM) between all human nGPCRs and their orthologs or co-orthologs in other species are shown in the lower panel.
(0.01 MB PDF)

Table S1 A. Inventory of nGPCR genes in human, rat, mouse, chicken, T. nigroviridis, and T. rubripes. B. List of nGPCR gene inventories in human, rat, mouse, chicken, T. nigroviridis, and T. rubripes. The accession number of individual nGPCRs in each species is listed according to their classification. For human nGPCRs, the gene ID is provided.
(0.07 MB PDF)

Table S2 A. Inventory of nGPCR genes in the MRCA of tetrapods and teleosts, and the number of derived nGPCR genes in human, rat, mouse, chicken, T. nigroviridis, and T. rubripes. The MRCA for each family of orthologous nGPCRs is defined as the gene that gave rise to a group of orthologs or co-orthologs in modern species. Each data point represents the total number of genes belonging to a select class or subclass of nGPCRs. The orthologous relationships of nGPCRs from different species are deduced by syntenic mapping and phylogenetic tree building analysis. B. List of nGPCR genes wherein a distinct evolutionary path can be traced from the MRCA of tetrapods and teleosts to modern species. The accession number of individual nGPCRs in each species is listed according to their classification. For human nGPCRs, the gene ID also is provided. Ancestral nGPCRs are denoted by the name of the human ortholog(s). C. List of nGPCR genes for which an ancestral form in the MRCA of tetrapods and teleosts cannot be defined. D. Correlation coefficients of nGPCR inventories between pairs of species. E. Number of singleton and duplicated nGPCR genes in model vertebrates, and number of nGPCR families with gene duplicates in each species.
(0.08 MB PDF)

Table S3 A. Tandem duplication-derived nGPCRs of T. nigroviridis. Tandem duplication-derived genes are defined as paralogous genes found on neighboring loci on the same chromosome. B. Summary of nGPCRs derived from tandem duplication in tetrapods. Tandem duplication-derived genes are defined as paralogous genes found on neighboring loci on the same chromosome. C. List of tandem duplication-derived nGPCRs of tetrapods. The putative origins of these tandem duplication-derived paralogs are indicated on the left column. D. WGD-derived nGPCRs of T. nigroviridis. WGD-derived genes are defined as co-orthologous genes found on WGD-derived syntenic regions on different chromosomes of pufferfish. E. Schematic representation of the WGD-derived GPR61 duplicates on T. nigroviridis chromosomes 9 and 11. The position of WGD-derived GPR61 and neighboring Alivin-2 genes on T. nigroviridis chromosomes as well as their orthologs on human chromosome 1 are indicated by italicized letters.
(0.03 MB PDF)

Table S4 A. List of the open reading frame (ORF) lengths of representative human receptors in each of the 269 nGPCR families inferred in the MRCA of tetrapods and teleosts. The lengths of human nGPCR ORFs were obtained from GenBank, and the majority of these ORFs have been defined experimentally. B. List of MWs of cognate ligands for each of the 190 families of nGPCRs with a known ligand(s). WGD, whole genome duplication; TD, tandem duplication; ND, not detected in T. nigroviridis; UD, undetermined; S, singleton.
(0.03 MB PDF)

Table S5 A. Inventory of polypeptide ligand genes in the MRCA of tetrapods and teleosts, human, and T. nigroviridis. B. List of polypeptide ligand genes in the MRCA of tetrapods and teleosts, and the derived ligand genes in human and T. nigroviridis. The accession number of identified ligands is listed. In cases where no existing accession number is available, the chromosomal position of the identified gene is provided. WGD, whole genome duplication; UD, undetermined; S, singleton. C. List of polypeptide ligands with WGD-derived duplicates in T. nigroviridis as well as their cognate receptors. Cognate receptors with WGD-derived duplicates in T. nigroviridis are shown in bold letters. The accession numbers for identified ligands are listed. In cases where no existing accession number is available, the chromosomal position of the identified gene is provided.
(0.04 MB PDF)
