# Supplementary material for: GPCR Genes Are Preferentially Retained after Whole Genome Duplication
Source: PLoS One. 2008 Apr 2;3(4):e1903. doi: 10.1371/journal.pone.0001903 (PMC2270905; doi:10.1371/journal.pone.0001903)

Fig. S1. Localization of WGD-derived adrenomedullin (ADM) and calcitonin/CGRP (CALCA) gene duplicates on syntenic regions of chromosomes 5 and 13 of *T. nigroviridis.*

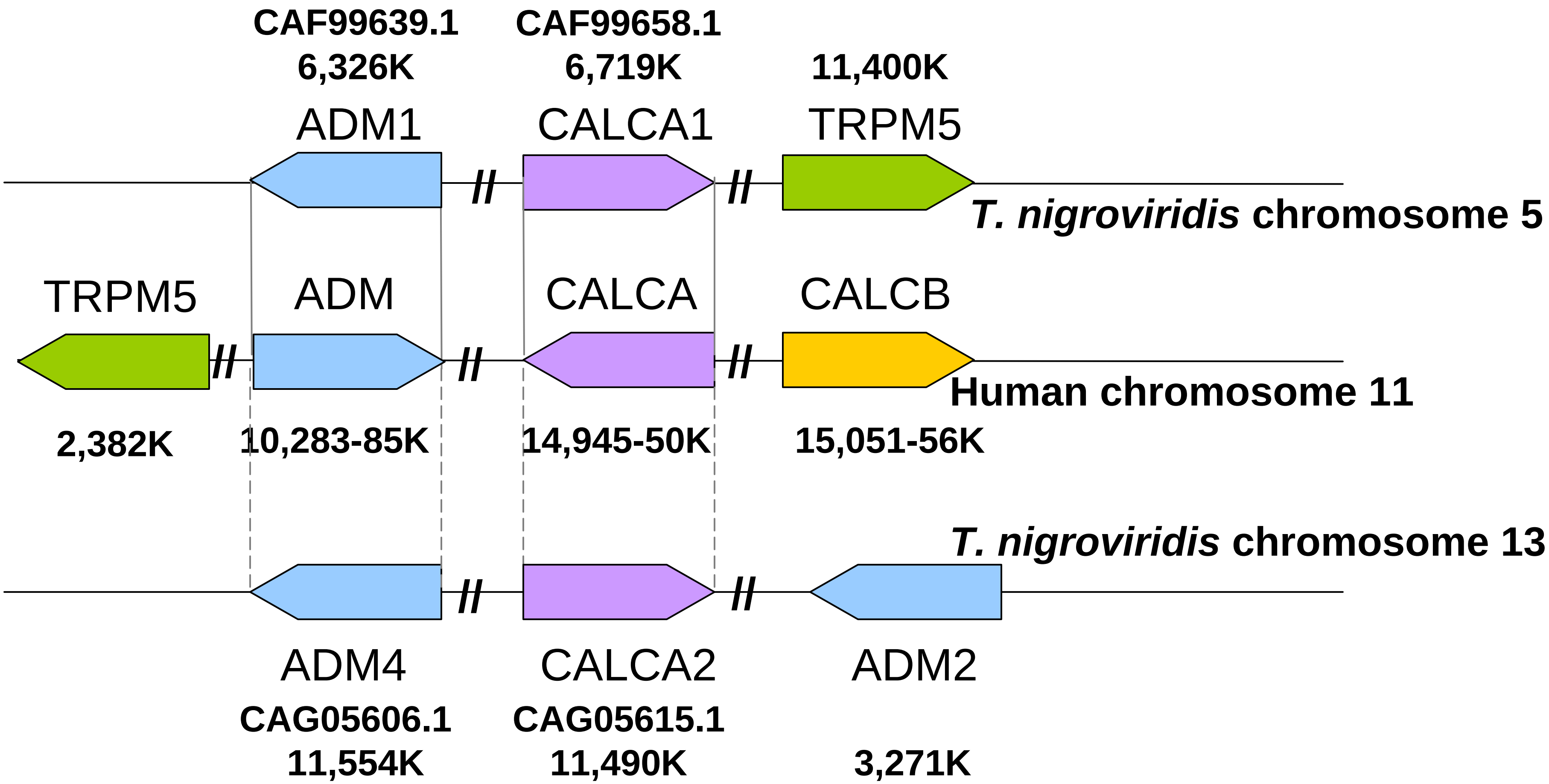

Supplement: Figure S1 — Localization of WGD-derived adrenomedullin (ADM) and calcitonin/CGRP (CALCA) gene duplicates on syntenic regions of chromosomes 5 and 13 of T. nigroviridis. (0.01 MB PDF) [file pone.0001903.s001.pdf]
