# Supplementary material for: GPCR Genes Are Preferentially Retained after Whole Genome Duplication
Source: PLoS One. 2008 Apr 2;3(4):e1903. doi: 10.1371/journal.pone.0001903 (PMC2270905; doi:10.1371/journal.pone.0001903)
Supplement: Figure S2 — Comparison of sequence similarity between human nGPCRs and orthologs or co-orthologs from rat, mouse, chicken, T. nigroviridis, and T. rubripes. Each data point represents the average of multiple data points belonging to brackets increased by a 5% step in sequence similarity as shown on the Y axis. The average sequence similarity and sequence identity (mean±SEM) between all human nGPCRs and their orthologs or co-orthologs in other species are shown in the lower panel. (0.01 MB PDF) [file pone.0001903.s002.pdf]

Fig. S2. Comparison of sequence similarity between human nGPCRs and orthologs or co-orthologs from rat, mouse, chicken, *T. nigroviridis*, and *T. rubripes*.

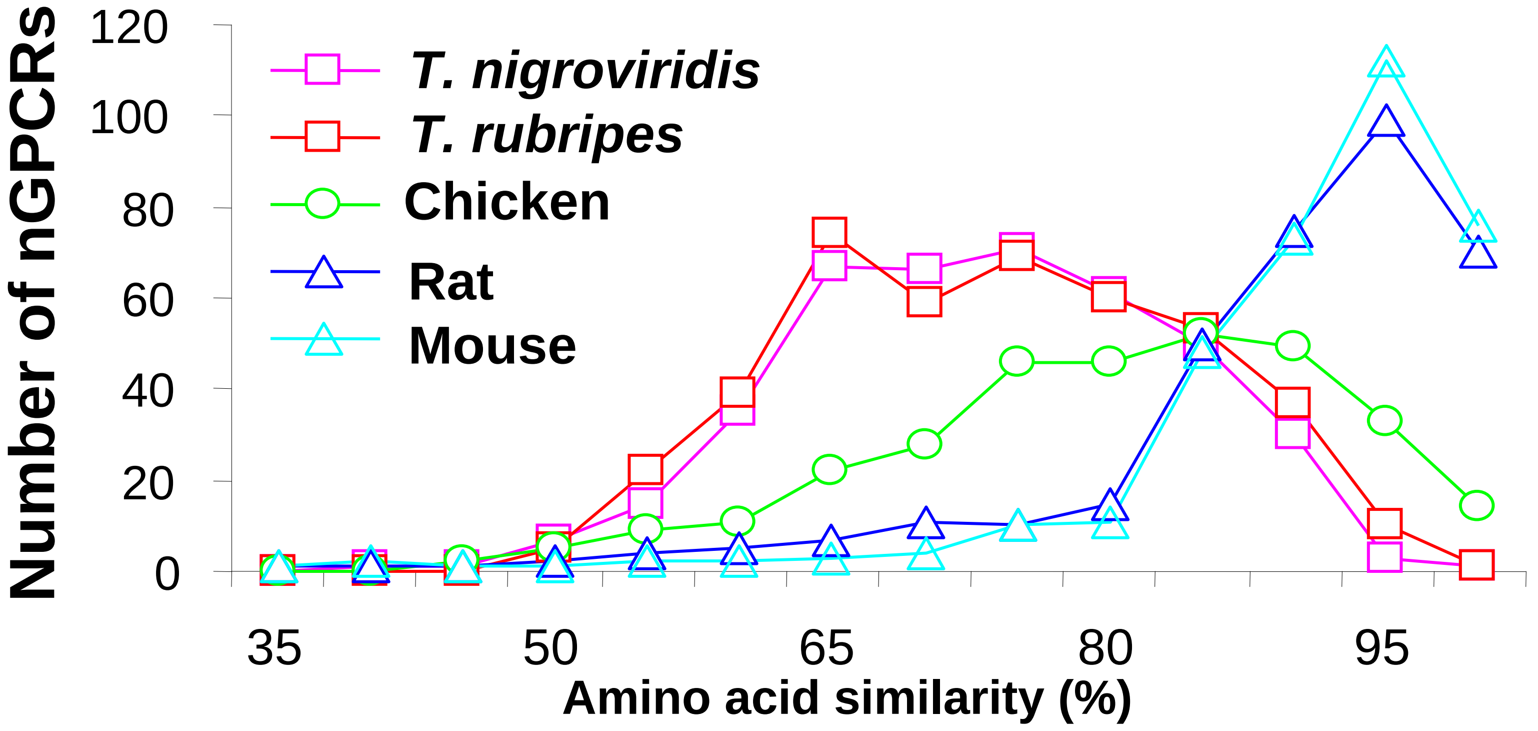

	Similarity, mean±SEM	Identity, mean±SEM
Human vs. rat	87.30±0.59	80.76±0.80
Human vs. mouse	88.77±0.55	82.99±0.70
Human vs. chicken	78.06±0.66	66.55±0.87
Human vs. *T. nigroviridis*	71.13±0.50	56.73±0.66
Human vs. *T. rubripes*	71.84±0.52	57.21±0.66
