# Supplementary material for: GPCR Genes Are Preferentially Retained after Whole Genome Duplication
Source: PLoS One. 2008 Apr 2;3(4):e1903. doi: 10.1371/journal.pone.0001903 (PMC2270905; doi:10.1371/journal.pone.0001903)
Supplement: Table S1 — A. Inventory of nGPCR genes in human, rat, mouse, chicken, T. nigroviridis, and T. rubripes. B. List of nGPCR gene inventories in human, rat, mouse, chicken, T. nigroviridis, and T. rubripes. The accession number of individual nGPCRs in each species is listed according to their classification. For human nGPCRs, the gene ID is provided. (0.07 MB PDF) [file pone.0001903.s003.pdf]

Table S1 A. Inventory of nGPCR genes in human, rat, mouse, chicken, *T. nigroviridis*, and *T. rubripes*.

Class	Human	Rat	Mouse	Chicken	*T. nigroviridis*	*T. rubripes*
**A1**	20	17	19	21	43	44
**A2**	53	63	62	52	84	81
**A3**	26	26	26	19	30	30
**A4**	49	40	44	40	46	46
**A5**	49	47	48	48	63	72
**A6**	8	8	8	8	10	10
**A7**	69	67	71	51	70	68
**A8**	10	20	31	4	0	0
**Subtotal**	**284**	**288**	**309**	**243**	**346**	**351**
**B**	48	44	46	42	53	50
**C**	16	16	16	14	19	23
**F**	11	11	11	11	13	14
**Subtotal**	**75**	**71**	**73**	**67**	**85**	**87**
**Total**	**359**	**359**	**382**	**310**	**431**	**438**

Table S1 B. List of nGPCR gene inventory in human, rat, mouse, chicken, *T. nigroviridis, and T. rubripes.*

Class A
Subclass A1

Gene Name	Human Gene ID	Rat	Mouse	Chicken	*T. nigroviridis*	*T. rubripes*
GPR26	2849	NP_620196	NP_775586	XP_421809	CAG00716<br>CAG03282	FRUP00000148603<br>FRUP00000160817
GPR50	9248	XP_346375	NP_034470	NP_990692	CAF99125	FRUP00000153084
GPR61	83873	XP_227581	NP_780679	NW_060850	CAG06826<br>CAG07504	
GPR62	118442	XP_576464	XP_488192		CAG06806<br>CAG06960	FRUP00000149305
GPR78	27201			XP_426354	GSTENT00035915001<br>CAG05074	FRUP00000132645<br>FRUP00000158903
GPR84	53831	GeneID_367000	NP_109645		CAG06758	FRUP00000162610
GPR101	83550	XP_229186	XP_141764		CAG09823<br>SCAF13862	FRUP00000144472
GPR135	64582	NP_861436	NP_861417	XP_426475	CAF98168	FRUP00000151143
MTNR1A	4543	XP_341442	NP_032665	NP_990693	CAF96594<br>CAG04524	FRUP00000158716<br>FRUP00000160445
MTNR1B	4544	XP_345900	NP_663758	XP_417201	SCAF14601	FRUP00000141583
OPN1LW	5956		GeneID_20164	NP_990771<br>NP_990740	CAF96877	FRUP00000164823
OPN1MW-fish					CAG06878<br>CAG08706<br>CAG10163	FRUP00000141947<br>FRUP00000144085<br>FRUP00000151874
OPN1MW	2652	NP_446000	NP_032132			
OPN1SW	611	NP_112277	NP_031564		CAF96876	FRUP00000164822
OPN3	23596		NP_034228	XP_426139<br>XP_425584	CAF89728<br>CAF97626<br>CAG08854<br>CAG09827	FRUP00000131360<br>FRUP00000140941<br>FRUP00000143705<br>FRUP00000155686
OPN4	94233	NP_620215	NP_038915	XP_421494<br>NP_989956	CAF90276<br>CAF96662<br>CAF99228<br>CAG08413	FRUP00000141781<br>FRUP00000141782<br>FRUP00000145811<br>FRUP00000145812<br>FRUP00000149714<br>FRUP00000163792
OPN5/GPR136	221391	NP_861437	NP_861418	XP_426228<br>XP_420056<br>XP_419178	CAF91597<br>CAG09534<br>CAG03922<br>CAG11258<br>CAG13006<br>CAG00077	FRUP00000133622<br>FRUP00000139290<br>FRUP00000145353<br>FRUP00000149081<br>FRUP00000154669<br>FRUP00000162507<br>FRUP00000164504<br>FRUP00000165286
RE2/GPR161	23432	XP_222837	XP_136361	XP_416594	CAG05829<br>CAG09451	FRUP00000138055<br>FRUP00000148240
RGR	5995	XP_224673	NP_067315	XP_421504	CAF98663<br>CAG13313	FRUP00000142438<br>FRUP00000148015
RHO	6010	NP_254276	NP_663358	XP_414454<br>NP_990821	CAG06941<br>CAG11315<br>CAG11334	FRUP00000136321<br>FRUP00000136323<br>FRUP00000156773<br>scaffold_200
RRH	10692	XP_227718	NP_033128	XP_420649	CAF94915	FRUP00000130657
**Subtotal**	**20**	**17**	**19**	**21**	**43**	**44**

Gene Name	Human Gene ID	Rat	Mouse	Chicken	*T. nigroviridis*	*T. rubripes*
**Subclass A2**						
ADORA1	134	NP_058851	NP_001008533	NP_989647	CAF96797<br>CAG11815<br>CAG07486	FRUP00000128561<br>FRUP00000143490
ADORA2A	135	NP_445746	NP_033760	XP_425280	CAF93754<br>CAG02468	FRUP00000141222<br>FRUP00000142941<br>FRUP00000144716
ADORA2B	136	NP_058857	NP_031439	NP_990418	CAF98510<br>CAG09178	FRUP00000154463
ADORA3	140	NP_037028	NP_033761	NP_989482	CAG11816	FRUP00000149785
ADRA1A	148	NP_058887	NP_038489	XP_425762	CAG11192	FRUP00000141240
ADRA1B	147	NP_058687	NP_031442	XP_414483	CAF92745<br>CAF97256	FRUP00000152462<br>FRUP00000164690
ADRA1D	146	NP_077809	NP_038488	XP_420871	CAF91280	FRUP00000140656
ADRA2A	150	NP_036871	NP_031443	XP_426537	CAG01293<br>CAG05052<br>CAG05356	FRUP00000127209<br>FRUP00000145339<br>FRUP00000154128
ADRA2B	151	NP_612514	NP_033763		CAG07800	FRUP00000129228
ADRA2C	152	NP_612515	NP_031444	XP_426355<br>XP_425203	CAG00720<br>CAG03287	FRUP00000136842<br>FRUP00000158905
ADRA2D-fish					CAF91577	CAC87886<br>CAC87887
ADRB1	153	NP_036833	NP_031445	XP_426540	CAG08867<br>CAG12607	FRUP00000128218<br>FRUP00000147601
ADRB2	154	NP_036624	NP_031446	XP_425195	CAG10129	FRUP00000128843<br>FRUP00000157526
ADRB3	155	NP_037240	NP_038490	XP_428541		
CHRM1	1128	NP_542951	NP_031724		CAG10670	FRUP00000131048
CHRM2	1129	NP_112278	NP_987076	XP_416359	CAG02408<br>CAG03403	FRUP00000127975<br>FRUP00000129486
CHRM3	1131	NP_036659	NP_150372	NP_990730	CAF92462<br>CAG10073	FRUP00000160548<br>scaffold_143
CHRM4	1132	XP_345404	NP_031725	XP_421119	CAG05625<br>CAG06610	FRUP00000147533<br>FRUP00000155473
CHRM5	1133	NP_059058	NP_991352	XP_426437	CAG03529	FRUP00000130167
DRD1	1812	NP_037269	NP_031610	XP_425206	CAF93510<br>CAF95884<br>CAG11724	FRUP00000154522<br>FRUP00000164051<br>FRUP00000164859
DRD2	1813	NP_036679	NP_034207	XP_425811	CAF92229<br>CAF97490<br>CAG13001	FRUP00000129665<br>FRUP00000143523<br>FRUP00000153968
DRD3	1814	NP_058836	NP_031903	XP_416570	CAG04235	scaffold_146
DRD4	1815	NP_037076	NP_031904	XP_420947	CAF95731<br>CAF98376	FRUP00000142755<br>FRUP00000149812
DRD5	1816	NP_036900	NP_038531	XP_426518<br>XP_426351	CAF90994<br>CAG02007	FRUP00000131890<br>FRUP00000138676
GPR21	2844	XP_231251	NP_796357	XP_415391	CAF93159<br>CAG09652	FRUP00000163990

Gene Name	Human Gene ID	Rat	Mouse	Chicken	*T. nigroviridis*	*T. rubripes*
GPR22	2845	XP_234041	NP_780400	XP_001231969	CAF89181<br>CAF96769<br>CAG02014<br>CAG08711	FRUP00000133859<br>FRUP00000162183<br>FRUP00000163060<br>scaffold_374
GPR52	9293	NW_047398	XP_890263	NP_001012941	CAG00742	FRUP00000163527
GPR57g/GPR57/ TAAR3	9288	NP_001009532	NP_001008429			
GPR58/TAAR2	9287	NP_001008512	NP_001007267	XP_427716	CAF94065	FRUP00000131967
GPR75	10936	XP_573685	NP_780699	XP_426099	SCAF15006	scaffold_905
GPR88	54112	NP_113884	NP_071872	XP_001232659		
GPR119	139760	NP_861435	NP_861416	XP_426720<br>XP_426248	CAF97243	FRUP00000143792
GPR160	26996	NP_001020318	XP_130823	XP_422799		
HRH1	3269	NP_058714	NP_032311	XP_425153	CAG07054	FRUP00000164084
HRH2	3274	NP_037097	NP_001010973	XP_425208	CAG01551<br>CAG04750	FRUP00000131597<br>FRUP00000138905
HRH3	11255	NP_445958	NP_598610	XP_425705<br>XP_425117		FRUP00000146131<br>FRUP00000165198
HRH4	59340	NP_571984	NP_694727	XP_426079<br>XP_414481	CAG07613<br>CAG10949	
HTR1A	3350	NP_036717	NP_032334	XP_429136	CAF91711<br>CAF93441	FRUP00000136865<br>FRUP00000154633
HTR1B	3351	NP_071561	NP_034612	XP_419875	CAF89927	FRUP00000161510
HTR1D	3352	NP_036984	NP_032335	XP_001232312	CAG10464	FRUP00000127520
HTR1E	3354			XP_001235179		
HTR1F	3355	NP_068629	NP_032336	XP_425535	CAG09227	FRUP00000155421
HTR2A	3356	NP_058950	NP_766400	XP_425628		
HTR2B	3357	NP_058946	NP_032337	AAF20211	CAC85912<br>CAC86247	FRUP00000147089
HTR2C	3358	NP_036897	NP_032338	XP_426265	CAG12257	FRUP00000135888
HTR4	3360	NP_036985	NP_032339		CAF93600<br>CAF95370<br>CAG04907	FRUP00000138443<br>FRUP00000140658<br>FRUP00000164424
HTR5A	3361	NP_037280	NP_032340	XP_426604<br>XP_425970	CAG04298	scaffold_934
HTR5B		XP_341112	NP_034613			
HTR6	3362	NP_077341	NP_067333	XP_427576	CAG01704	FRUP00000139170<br>FRUP00000139171
HTR7	3363	NP_075227	NP_032341	XP_421666<br>XP_420880	CAG05134<br>CAG09680	FRUP00000139991<br>FRUP00000144402<br>FRUP00000144993
PNRe/PNR/TAAR5	9038	NP_001009650	NP_001009574<br>NP_038736	XP_419740		
TRAR1/Tar1/TAAR1	134864	NP_599155<br>NP_783173	NP_444435		CAF91012<br>CAF92549<br>CAF92550<br>CAF92551<br>CAF93538<br>CAF93617<br>CAF93618<br>CAF94841	FRUP00000131749<br>FRUP00000138381<br>FRUP00000150777<br>FRUP00000150779<br>FRUP00000154655<br>FRUP00000157293<br>scaffold_2245

Gene Name	Human Gene ID	Rat	Mouse	Chicken	*T. nigroviridis*	*T. rubripes*
					CAF95185<br>CAF95186<br>CAF95187<br>CAF96106<br>CAG13003	
TRAR3f/TAR3	134860	NP_783175<br>NP_783176<br>NP_001009975<br>NP_783181<br>NP_783180<br>NP_783178<br>NP_783177<br>NP_783192	NP_001010831<br>NP_001010827<br>NP_001010829<br>NP_001010835<br>NP_001010838<br>NP_001010839			
TRAR4/TAR4/TAAR6	319100	NP_783174	NP_001010828<br>NP_001008499			FRUP00000157578<br>FRUP00000135638
TRAR5/GPR102/TA5/<br>TAR5/TAAR8	83551	NP_783189<br>NP_783191<br>NP_783190	NP_001010830<br>NP_001010840<br>NP_001010837			
**Subtotal**	**53**	**63**	**62**	**52**	**84**	**81**

Subclass A3

CNR1	1268	NP_036916	NP_031752	XP_426191	CAG09211<br>CAG10478	FRUP00000157187<br>CAA64174
CNR2	1269	NP_065418	NP_034054	XP_425791	CAF91752	FRUP00000161224
EDG1	1901	NP_058997	NP_031927	XP_422305	CAF90490	scaffold_6096
EDG2	1902	NP_446388	NP_034466	XP_424918	CAF89669<br>CAF90900<br>CAF92197	FRUP00000154449<br>scaffold_86
EDG3	1903	XP_225216	NP_034231	XP_428818	CAG07943<br>CAG12442	FRUP00000154122<br>scaffold_270<br>scaffold_270
EDG4	9170	XP_573887	NP_064412	XP_423763		scaffold_799
EDG5	9294	NP_058888	NP_034463		SCAF15050<br>chr3_11567980_8876	scaffold_180
EDG6	8698	XP_234930	NP_034232	XP_428050		scaffold_203
EDG7	23566	NP_076459	NP_075359	XP_422368	CAG08072	FRUP00000148649
EDG8	53637	NP_068543	NP_444420		CAF92590	FRUP00000139298
GPR12	2835	NP_110458	NP_001010941	XP_425637	CAG04221<br>CAG12255	FRUP00000158473
GPR3	2827	NP_714949	NP_032180		CAF88618	FRUP00000160201
GPR6	2830	NP_113994	NP_951013	XP_426182	CAG03572	FRUP00000134973
MC1R/MSHR	4157	EDL92809	NP_032585	XP_425135	AAQ55176	AAO65548
MC2R	4158	XP_574167	NP_032586	XP_426070	CAF95601	
MC3R	4159	NP_001020441	NP_032587	NP_001026186		
MC4R	4160	NP_037231	NP_058673	XP_426042	AAQ55178	AAO65551
MC5R	4161	NP_037314	NP_038624	NP_001026186	AAQ55179	AAO65553
PTGDR	5729	NP_071577	NP_032988			
PTGER1	5731	NP_037232	NP_038669			
PTGER2	5732	NP_112350	NP_032990	XP_426485	CAG00989	scaffold_1339

Gene Name	Human Gene ID	Rat	Mouse	Chicken	*T. nigroviridis*	*T. rubripes*
PTGER3	5733	NP_036836	NP_035326	XP_426672	CAG01184	FRUP00000131256
PTGER4	5734	NP_114465	NP_032991	XP_424770	CAF95032<br>CAF96390<br>CAF97110<br>CAG09586	FRUP00000155267<br>FRUP00000157769<br>FRUP00000161139<br>FRUP00000162687
PTGFR	5737	NP_037247	NP_032992	NP_001038122	CAG03303	FRUP00000149163<br>FRUP00000151130
PTGIR	5739	XP_218457	NP_032993		SCAF14537	FRUP00000130879
TBXA2R	6915	NP_058750	NP_033351		CAG00626	FRUP00000152568
**Subtotal**	**26**	**26**	**26**	**19**	**30**	**30**

Subclass A4

ADMR	11318	NP_445754	NP_031438		CAF90054	FRUP00000146637
CMKOR1/RDC1	57007	NP_445804	NP_031748	XP_426554	CAG05877	scaffold_387
CYSLTR1	10800	NP_446093	NP_067451	XP_426249	SCAF7335	FRUP00000150747
CYSLTR2	57105	NP_596904	NP_598481	XP_425629		
EBI2	1880		NP_898852	XP_428804	CAG07299<br>chr2_7825313-7877092	FRUP00000150142<br>FRUP00000161432
F2R	2149	NP_037082	NP_034299	XP_424799	CAG07882	FRUP00000152221
F2RL1	2150	NP_446349	NP_032000	NP_001012626	CAG05264<br>CAG05265	FRUP00000130845<br>FRUP00000130847
F2RL2	2151	NP_445765	NP_034300	XP_414523	CAG05266<br>CAG07880	FRUP00000151261<br>FRUP00000129585<br>FRUP00000130849
F2RL3	9002	NP_446260	NP_032001	XP_418256	CAF95426	chrUn_311834154-530
GPR18	2841		NP_877958	NW_060236	CAG07300	FRUP00000150143
GPR20	2843	NP_071552	NP_775541	XP_425941	CAF99361<br>CAG11945	FRUP00000136628<br>FRUP00000165047
GPR23	2846	XP_228501	NP_780480	XP_425741<br>XP_420150	CAF90112<br>CAG03809	FRUP00000150750
GPR31	2853	XP_217867	GeneID_107431			
GPR34	2857	NP_001020096	NP_035953	XP_420190	SCAF14978	scaffold_1642
GPR35	2859	NP_001032436	NP_071715	XP_422645		
GPR4	2828	NP_001020851	NP_783599		CAF97465<br>CAG12733	FRUP00000128368<br>FRUP00000135821
GPR40	2864	NP_695216	NP_918946			
GPR41	2865	XP_344881	XP_145470		CAF89689	FRUP00000153029
GPR42a/GPR42	2866					
GPR43	2867	NP_001005877	NP_666299	XP_428780<br>XP_426943	CAF93280<br>CAG03362	FRUP00000129763<br>FRUP00000161234
GPR55	9290	AAD22411	NP_001028462			
GPR65	8477	XP_234367	NP_032178	XP_421305	SCAF15019	FRUP00000150188
GPR68	8111	XP_234483	NP_780702	XP_426459	CAF99011	FRUP00000136318
GPR81	27198	XP_001072594	NP_780729			
GPR82	27197		NP_783600	NW_060226		
GPR87	53836	XP_227177	NP_115775	XP_422840		

Gene Name	Human Gene ID	Rat	Mouse	Chicken	*T. nigroviridis*	*T. rubripes*
GPR92	57121	XP_575667	XP_355812	XP_416504		
GPR109A/HM74a	338442	NP_852141	NP_109626		CAF90987<br>CAG05298<br>CAG08446	FRUP00000136429<br>FRUP00000146889<br>FRUP00000148806<br>FRUP00000150723
GPR109B/HM74	8843					
GPR132/G2A	29933	XP_234574	NP_064309	XP_426469		
GPR141/PGR13	353345	NP_861432	NP_861419	XP_418832	SCAF14544	scaffold_240
GPR17	2840	NW_047510	AAH70439		CAG04290<br>CAG05650	FRUP00000128875<br>FRUP00000147658
GPR171/H963	29909		NP_775574	XP_422842		
GPR174/FKSG79	84636	XP_228495	NP_001028423	NP_001008464	CAG14148	scaffold_1393
OXER1/TG1019	165140					
OXGR1/GPR80	27199	NP_997471	NP_001001490	XP_425595<br>XP_425594	CAG04953	FRUP00000159316
P2RY1	5028	NP_036932	NP_032798	XP_427676<br>NP_990664	CAF97378<br>CAG03842	FRUP00000138058
P2RY10	27334	XP_228500	NP_766023	XP_420149<br>XP_420148		
P2RY11	5032				CAG12186	FRUP00000147765
P2RY12	64805	NP_073637	NP_081847	XP_422839	CAF97387	FRUP00000138083
P2RY13/GPR86	53829	NP_001002853	NP_083084		CAF97388	scaffold_239
P2RY14/GPR105	9934	NP_598261	NP_573463	XP_422841	SCAF14677	
P2RY2	5029	NP_058951	NP_032799	XP_425667	CAG00917<br>CAF97358	FRUP00000127686<br>FRUP00000141264<br>FRUP00000165330
P2RY4	5030	NP_113868	NP_065646	XP_420157	CAG09809	FRUP00000163721
P2RY6	5031	NP_476465	NP_898991	NP_990526	CAG00913	FRUP00000151445
P2RY8	286530			NP_001008679		FRUP00000139369
P2Y5/P2RY5	10161	NW_047454	NP_780325	NP_990530	CAG02919<br>CAG12785	FRUP00000141960
PTAFR	5724	NP_445773	XP_357441	XP_425768	CAG10424	FRUP00000134471
SUCNR1/GPR91	56670	XP_579061	NP_115776	XP_422836	CAF97381	FRUP00000138067
**Subtotal**	**49**	**40**	**44**	**40**	**46**	**46**

Subclass A5

AVPR1A	552	NP_444178	NP_058543	XP_425436	CAF96006<br>CAG12390	FRUP00000141525<br>FRUP00000161762
AVPR1B	553	NP_058901	NP_036054	XP_425822<br>XP_425434	CAF96931	scaffold_8423
AVPR2	554	NP_062009	NP_062277		CAG03798<br>CAG07446	FRUP00000161883<br>scaffold_323
BRS3	680	NP_690058	NP_033896	NP_989737		
CCKAR	886	NP_036820	NP_033957	XP_420751	CAG04576	FRUP00000133099
CCKBR	887	NP_037297	NP_031653	NP_001001742		
CCK-fish					CAG00996	FRUP00000135669<br>FRUP00000162216

Gene Name	Human Gene ID	Rat	Mouse	Chicken	*T. nigroviridis*	*T. rubripes*
					CAG05857	FRUP00000162217
EDNRA	1909	NP_036682	NP_034462	NP_989450	CAG02202<br>CAG11066	FRUP00000133802<br>FRUP00000133804<br>FRUP00000133807<br>FRUP00000157593<br>FRUP00000157598
EDNRB	1910	NP_059029	NP_031930	XP_417001<br>NP_989451	CAF90114<br>CAF97649<br>CAG05905	FRUP00000141004<br>FRUP00000141006<br>FRUP00000150746<br>FRUP00000163212
FSHR	2492	NP_954707	NP_038551	NP_990410	CAF91019	scaffold_49
GNRHR	2798	NP_112300	NP_034453	NP_989984		
GNRHR2	114814			NP_001012627	CAF92557<br>CAF93245<br>CAG00826<br>CAG03087<br>CAG13098	FRUP00000134302<br>FRUP00000138360<br>FRUP00000141711<br>FRUP00000156324<br>FRUP00000160897
GPRe/GPR/GPR176	11245	XP_342494	NP_958755	XP_426435	CAG03525	FRUP00000131045
GPR10	2834	NP_631932	NP_963909	XP_428805<br>NP_001019756<br>NP_001012295	CAF97466<br>CAF99282	FRUP00000135820<br>FRUP00000165587
GPR19	2842	NP_542146	NP_032183	XP_428465	CAF90680	FRUP00000131864
GPR27	2850	NP_075587	NP_032184			
GPR37	2861	NP_476549	NP_034468	XP_415992	CAG01068	FRUP00000137022<br>FRUP00000140117<br>scaffold_2384<br>scaffold_3299
GPR37L1/ET(B)R-LP-2	9283	XP_573457	NP_602320	XP_419256	SCAF7416	FRUP00000136126<br>FRUP00000142342
GPR45	11250	XP_237112	NP_444337		CAG05802	FRUP00000163802
GPR63	81491	XP_232847	NP_109658	XP_426189	CAF93484	FRUP00000149653
GPR73/PKR1	10887	NP_620433	NP_067356	XP_419333	CAF98618	FRUP00000139538<br>FRUP00000154172
GPR73L1/PKR2	128674	NP_620434	NP_659193	XP_419334		
GPR74/NPFF receptor 2	10886	NP_076470	NP_573455	XP_426315	CAF97104<br>CAG07773	FRUP00000155275<br>FRUP00000155634
GPR83	10888	NP_536336	NP_034417	XP_426254<br>XP_425651	CAG06215	FRUP00000164362
GPR85	54329	NP_071590	NP_659503	XP_416019	CAG12125	FRUP00000143116
GPR103/QRFP	84109	NP_937842	NP_780733	XP_420626	CAG04805<br>SCAF14750	FRUP00000142516
GPR147/NPFFR1	64106	NP_071627	XP_137119	NP_989693	CAG09719	FRUP00000157824
GPR150/PGR11	285601	XP_574811	NP_780704			
GPR154/NPS receptor	387129	XP_235968	NP_783609	XP_426022		
GPR173/SREB3	54328	NP_071591	NP_081819	XP_414427	CAF93024<br>CAF96921<br>CAG03946	FRUP00000150812<br>FRUP00000153190<br>FRUP00000155856
GRPR	2925	NP_036838	NP_032203	NP_989738	CAF99824<br>CAG05835	FRUP00000156510<br>FRUP00000161978
HCRTR1	3061	NP_037196	NP_945197			
HCRTR2	3062	NP_037206	NP_945200	NP_001019755	CAG09420	FRUP00000163192

Gene Name	Human Gene ID	Rat	Mouse	Chicken	*T. nigroviridis*	*T. rubripes*
LGR4	55366	NP_775450	XP_355385	XP_426162	CAF99699	scaffold_826
LGR5	8549	XP_235149	NP_034325	XP_425441	CAG14806	FRUP00000149831
LGR6	59352	XP_573455	XP_283647	XP_419253	CAG07358	FRUP00000148236<br>FRUP00000148237
LGR7	59350	NP_958820	NP_997617	XP_426253	CAG04641	scaffold_1619
LGR8	122042	XP_344074	NP_569720		CAG00416	FRUP00000159568
LHCGR	3973	NP_037110	NP_038610	NP_990267	CAF91017<br>CAG09466	
NMBR	4829	NP_036931	NP_032729	XP_426167	CAF94959<br>CAG14163	FRUP00000150118<br>GSTENT00011415001
NPY1R	4886	NP_001013050	NP_035064	XP_426285		
NPY2R	4887	NP_076458	NP_032757	XP_420373<br>XP_414599	CAF96589<br>SCAF14573	FRUP00000138240<br>DAA05226
NPY5R	4889	NP_037001	NP_057917	XP_420388	CAG02528<br>CAG05410	FRUP00000160782<br>FRUP00000160783
NPY6R/NPY1RL/PP2/ Y2B	4888		NP_035065			
OXTR	5021	NP_037003	XP_144956	XP_426768	CAG06809<br>CAG06958	FRUP00000127611<br>FRUP00000149294
PPYR1	5540	NP_113769	NP_032945	XP_426511	CAG09405	FRUP00000164394
TACR1	6869	NP_036799	NP_033339	NP_990199	CAG05392<br>CAG12579	FRUP00000138587<br>FRUP00000148382<br>FRUP00000160726
TACR2	6865	NP_542946	NP_033340	XP_426496		
TACR3	6870	NP_058749	NP_067357	XP_001232174	CAG01274<br>CAG11520<br>CAG05682	FRUP00000132595<br>FRUP00000135143<br>FRUP00000147917
TSHR	7253	NP_037020	NP_035778	XP_426455	CAG00111	FRUP00000145651
**Subtotal**	**49**	**47**	**48**	**48**	**63**	**72**

Subclass A6

GHSR	2693	NP_114464	NP_796304	NP_989725	CAF95899	FRUP00000131849
GPR39	2863	DAA06056	AAH85285	XP_422128	CAG11238	FRUP00000135301
MLNR/GPR38	2862			XP_425630	CAG12790	FRUP00000138615
NMUR1	10316	NP_075588	NP_034471	XP_426705	CAF97543<br>CAG03466	FRUP00000153653<br>FRUP00000156953
NMUR2/NMU2R	56923	NP_071611	NP_694719	XP_425209	CAG03508	FRUP00000147487
NTSR1	4923	XP_345485	NP_061236	XP_425707	CAF89857	FRUP00000128303
NTSR2	23620	NP_073186	NP_032773			
TRHR	7201	NP_037179<br>NP 852029	NP_038724<br>NP 573465	XP_425702<br>NP_990261	CAF92799<br>CAF96270<br>CAG03888	FRUP00000134073<br>FRUP00000146669<br>FRUP00000153993
**Subtotal**	**8**	**8**	**8**	**8**	**10**	**10**

Subclass A7

AGTR1	185	NP_112271	NP_796296<br>NP_780295	NP_990488	CAG04270	FRUP00000150672
AGTR2	186	NP_036626	NP_031455	XP_426266	CAG08373	scaffold_1395
AGTRL1	187	NP_112639	NP_035914	XP_425289	CAF90289<br>CAF99292	FRUP00000134029<br>FRUP00000134030

Gene Name	Human Gene ID	Rat	Mouse	Chicken	*T. nigroviridis*	*T. rubripes*
					SCAF12244	FRUP00000146396<br>FRUP00000165603
BDKRB1	623	NP_110478	NP_031565	NP_001074189	CAG10408	FRUP00000150183<br>FRUP00000150187
BDKRB2	624	NP_775123	NP_033877	XP_426461		
BLR1/CXCR5	643	NP_445755	NP_031577	NP_001026083	CAG09055	FRUP00000156705
C3AR1	719	NP_114449	NP_033909	XP_416428		
C5R1	728	NP_446071	NP_031603		CAG02859	FRUP00000134135
CCBP2	1238	NP_511176	NP_067622	XP_418499		
CCR1	1230	NP_065417<br>XP_236742	NP_034042			
CCR2	1231	NP_068638	NP_034045			
CCR3	1232	NP_446410	NP_034044			
CCR4	1233	NP_598216	NP_034046	XP_426017	chr8_3997729_8596	FRUP00000127130
CCR5	1234	NP_446412	NP_034047	XP_418796<br>XP_418795		
CCR6/GPR29	1235	NP_001013163	NP_033965	XP_419608	CAF92962	FRUP00000146532
CCR7	1236	NP_955783	NP_031745	XP_425875	CAG06152	FRUP00000154746
CCR8	1237	XP_236704	NP_031746	XP_418819		
CCR9/GPR28	10803	NP_758832	NP_034043	XP_426014	CAF90274<br>CAG05508	FRUP00000142615
CCR10/GPR2	2826	XP_343969	NP_031747		chr2_1817756-1818529	FRUP00000140298
CCR11	51554	Q9ESK1	NP_663746		CAF88488	FRUP00000138457
CCRL2	9034	XP_236658	NP_059494			
CMKLR1	1240	NP_071554	NP_032179	XP_425239<br>XP_423260	CAF94854<br>CAF94856<br>CAG08499	FRUP00000145765<br>FRUP00000149103<br>FRUP00000155117
CX3CR1	1524	NP_598218	NP_034117	XP_418820	chr8_854312-855332	chrUn:212102004-3119
CXCR3/GPR9	2833	NP_445867	NP_034040		CAF98051<br>CAF98053	FRUP00000138635<br>FRUP00000138636
CXCR4	7852	NP_071541	NP_034041	NP_989948	CAF97662<br>CAG01848	FRUP00000134232<br>FRUP00000137823
CXCR6	10663		NP_109637	AAC23950		
FPR1	2357	XP_218012	NP_038549		CAG11673	scaffold_3925
FPRL1	2358	XP_001057995	NP_032068			
FPRL2	2359	XP_218016<br>XP_218022<br>XP_001073753	XP_622087<br>EDL38017<br>AAC34587<br>AAN63620<br>AAN63621			
FY	2532	NW_047399	NP_034175			
GALR1	2587	NP_037090	NP_032108	XP_426066<br>XP_414080	CAG00818<br>CAG12842	FRUP00000149875<br>FRUP00000153302
GALR2	8811	NP_062045	NP_034384	XP_428968	CAG12230	FRUP00000158738
GALR3	8484	NP_062046	NP_056553			

Gene Name	Human Gene ID	Rat	Mouse	Chicken	*T. nigroviridis*	*T. rubripes*
GPR1	2825	NP_037093	NP_666362	XP_421955		FRUP00000131794
GPR15	2838	XP_221546	XP_156321	XP_425528		
GPR24/MCH receptor 1	2847	NP_113946	NP_660114	XP_420129	CAF94024	FRUP00000130394
GPR25	2848	XP_344147	XP_357126		CAG11769	
GPR30	2852	NP_598257	NP_084047	XP_414765	CAG12216	FRUP00000146157
GPR32	2854					
GPR33	2856	NP_001026993	NP_032185			
GPR44	11251	NP_001012070	NP_034092		CAG03720	FRUP00000149198
GPR54/Kiss receptor	84634	NP_076482	NP_444474		CAG06231	FRUP00000131919
GPR7	2831	NP_001014784	XP_136404	XP_419201		
GPR77	27202	NP_001003710	NP_795886	XP_428039		
GPR8	2832			XP_425710	SCAF14682	
GPR120	338557	XP_215281	NP_861413	XP_426520		
GPR139/PGR3	124274	NP_001019412	XP_146103	XP_428970	CAG11412	AAP72123
GPR142	350383	XP_577136	NP_861414	XP_428957	CAG03049	AAP72122
GPR145/MCH receptor 2	84539				CAF99158	FRUP00000144995
GPR146	115330	XP_573364	NP_084534	XP_414764	CAG12217	scaffold_710
GPR151	134391	Q7TSN5	NP_853521		CAG01252<br>CAG10137	FRUP00000127188<br>scaffold_544
GPR152	390212	XP_001068404	NP_996856			
IL8RA	3577	NP_062183	NP_839972		CAF98991	FRUP00000127720
IL8RB	3579	NP_058879	NP_034039	XP_428206	CAF98209<br>chr2_16451193_2269	FRUP00000131991<br>FRUP00000127485
LTB4R	1241	NP_067688	NP_032545	XP_425304<br>XP_413985	CAF97025<br>CAF97026<br>SCAF14685	FRUP00000157228<br>FRUP00000159672<br>FRUP00000164123
LTB4R2	56413	NP_446092	NP_065236			
OPRD1	4985	NP_036749	NP_038650		CAG00040	FRUP00000153740
OPRK1	4986	NP_058863	NP_035141	XP_426087	CAG10221<br>CAG12280	FRUP00000142489
OPRL1	4987	NP_113757	NP_035142	XP_417424	CAG02324	FRUP00000132165
OPRM1	4988	NP_037203	NP_001034741	XP_419683	CAG10056	FRUP00000162543
RLN3R1/GPCR135/SALPR	51289	NP_001008311	NP_848832	XP_429217	CAF97176<br>CAG01356<br>CAG07748	FRUP00000153586<br>FRUP00000155748
RLN3R2/GPCR142/ GPR100	339403		NP_861538		CAF89883<br>CAF99357<br>CAF99875<br>CAF99890<br>CAF91545	FRUP00000127644<br>FRUP00000129142<br>FRUP00000136065
SSTR1	6751	EDM03458	NP_033242	XP_001234905		scaffold_707
SSTR2	6752	NP_062221	NP_033243	XP_425384	CAF96985<br>CAF96986	AAL32173<br>GSCT00010055001_prot<br>scaffold_1772

Gene Name	Human Gene ID	Rat	Mouse	Chicken	*T. nigroviridis*	*T. rubripes*
SSTR3	6753	NP_598206	NP_033244	NP_001019754	CAF90874<br>CAG12520	scaffold_958<br>scaffold_927<br>scaffold_958
SSTR4	6754	NP_037168	NP_033245	XP_426102		
SSTR5	6755	NP_037014	XP_139909	XP_425241	CAG06053	
UTS2R/GPR14	2837	NP_065412	NP_663415	XP_425371<br>XP_425370<br>XP_425234<br>XP_416275	CAF97882<br>CAG02581<br>CAG02582<br>CAG12512	FRUP00000129229<br>FRUP00000164718<br>scaffold_2802
XCR1/GPR5	2829	XP_236740	NP_035928	XP_426012	CAG11634<br>chrUn_120676885_7499	FRUP00000159812<br>chrUn:277232531_5721<br>chrUn:164653540_7809
CC-fish	fish_4				chr8_3990155_1408	chrUn:197058105_9697
**Subtotal**	**69**	**67**	**71**	**51**	**70**	**68**

Subclass A8

Gene Name	Human Gene ID	Rat	Mouse	Chicken	*T. nigroviridis*	*T. rubripes*
MAS1	4142	NP_036889	NP_032578	XP_427992<br>XP_427991<br>XP_426151<br>XP_423677		
MAS1L	116511					
MRGPRD	116512	NP_001001506	NP_987075			
MRGPRE	116534	NP_001002288	NP_780743			
MRGPRF/MRGF	116535	NP_714944	NP_663354			
MRGPRG/MRGG	386746	NP_982296	NP_987077			
MRGPRH		NP_001002281	NP_109651			
MRGX1e/MRGX1	259249					
MRGX2e/MRGX2	117194	NP_665730<br>NP_001002282<br>NP_001002286<br>NP_750845	NP_694735<br>NP_694741<br>NP_694707<br>NP_705744<br>NP_997417<br>NP_991390<br>NP_997418<br>NP_997419<br>NP_997423<br>AX299180<br>AX299182<br>AX299184<br>AX299186<br>AX299188<br>AX299190<br>AX299192<br>AX299194<br>AX299200			
rat_MrgB3		NP_001002280<br>NP_001002285<br>AAQ08313<br>NP_001002287<br>NP_001002284<br>AAQ08316<br>GeneID_404639<br>NP_001002283<br>GeneID_404654<br>GeneID_404657	NP_780740<br>NP_997420<br>NP_991364<br>NP_997421<br>NP_997422<br>NP_991379<br>AX299214			
MRGX3e/MRGX3	117195					
MRGX4e/MRGX4	117196					
**Subtotal**	**10**	**20**	**31**	**4**	**0**	**0**

Gene Name	Human Gene ID	Rat	Mouse	Chicken	*T. nigroviridis*	*T. rubripes*
**Class B**						
ADCYAP1R1	117	NP_598195	NP_031433	XP_425958<br>XP_418491	CAF94168<br>CAF95249<br>CAF95250<br>CAG12267<br>CAG12268	FRUP00000135842<br>FRUP00000161798<br>FRUP00000161801<br>FRUP00000161807
BAI1	575	XP_343261	NP_778156	XP_418415	CAF97520<br>CAG10859	FRUP00000164474
BAI2	576	XP_232778	NP_775094	XP_423828	CAF99436	
BAI3	577	XP_217367	NP_783573	XP_419892	CAG09441<br>GSTENT00030703001	FRUP00000129349<br>FRUP00000143645<br>FRUP00000143649
CR	799	NP_446268	NP_031614	XP_425985	CAG11122<br>CAG13459	FRUP00000144138<br>FRUP00000147908
CLR	10203	NP_036849	NP_061252	XP_421850	CAF94370<br>CAF98969<br>CAG01331	FRUP00000145573<br>FRUP00000148605
CD97	976	NP_001012164	NP_036055		CAG12162	FRUP00000150608
CELSR1	9620	XP_235570	NP_034016	XP_423746	CAG01167	
CELSR2	1952	XP_342319	NP_059088	XP_428195<br>XP_426750	CAG06842	FRUP00000162857
CELSR3	1951	NP_112610	NP_536685	XP_414354	CAG03262	FRUP00000139273
CRHR1	1394	NP_112261	NP_031788	NP_989652	CAG03022	FRUP00000135485
CRHR2	1395	NP_073205	NP_034083	NP_989785		scaffold_515
ELTD1	64123	NP_071630	NP_573485	XP_422383	CAG01189	FRUP00000131264
EMR1	2015	NP_001007558	NP_034260		CAG04105	
EMR2	30817					
EMR3	84658					
EMR4b/EMR4	326342		NP_631877			scaffold_1738
GCGR	2642	NP_742089	NP_032127	XP_423402	CAG01110<br>CAG03726	FRUP00000135578
GHRHR	2692	NP_036982	NP_001003685	XP_418490	CAF95846	FRUP00000146088<br>FRUP00000146089
GIPR	2696	NP_036846	NP_001074284			
GLP1R	2740	NP_036860	NP_067307	XP_426129	CAG04358	FRUP00000146170
GLP2R	9340	NP_068620	NP_783612	XP_425358	CAF96232	FRUP00000147245
GPR56	9289	NP_689448	NP_061370	XP_413999	CAG00694	
GPR64	10149	NP_852031	NP_848827	XP_416810	CAG00549<br>CAG05443	FRUP00000143610<br>FRUP00000159461
GPR97	222487	XP_226243	NP_766624	XP_413998		FRUP00000158285
GPR110	266977	XP_217359	NP_598537			
GPR111	222611	XP_236958	XP_487485			
GPR112	139378	XP_229201	XP_141802	XP_420232	CAG09829<br>CAG11729	FRUP00000140064<br>FRUP00000140065<br>FRUP00000159797
GPR113/PGR23	165082	XP_233935	XP_355587			FRUP00000150114

Gene Name	Human Gene ID	Rat	Mouse	Chicken	*T. nigroviridis*	*T. rubripes*
GPR114	221188	XP_240979	XP_356118	XP_414000	CAG00691	
GPR115	221393		XP_900079	XP_420068		
GPR116	221395	NP_620810	XP_283438	XP_420066	CAG13000	FRUP00000153997
GPR123	84435	XP_219468	NP_803420	XP_421695	CAG10157	
GPR124	25960	EDM09091	NP_473385	ENSGALP00000004917	CAF90106	FRUP00000146680
GPR125	166647	XP_223485	XP_132089	XP_420763	CAF90021	FRUP00000153701
GPR126	57211	XP_218313	NP_001002268	XP_419712	CAF94956	FRUP00000157379
GPR128	84873	XP_221527	NP_766413			
GPR133	283383	XP_001078502	NP_001074811	XP_415094		FRUP00000137738
GPR144	347088	XP_578117	XP_355327	XP_415385	CAG01306	scaffold_1674
LPHN1	22859	NP_599235	NP_851382	XP_422382	CAG06092<br>chr3_11032320_65518	
LPHN2	23266	NP_075251	NP_001074767	XP_422209<br>XP_422381	CAF95119<br>CAF98480	
LPHN3	23284	NP_570835	NP_941991	XP_420575	CAF93446<br>CAG02284	
MASS1/VLGR1	84059	XP_342370	XP_358310	XP_429120	CAG00389	FRUP00000164895<br>FRUP00000164896
PTHR1	5745	NP_064458	NP_035329	XP_425837	CAF98426<br>CAG12650	FRUP00000144315<br>FRUP00000144316<br>FRUP00000162177
PTHR2	5746	NP_112351	NP_644676	XP_418507	CAF97204	FRUP00000128587<br>FRUP00000128588
SCTR	6344	NP_112377	NP_001012322	XP_422120		
VIPR1	7433	NP_036817	NP_035833	XP_418492<br>NP_001006328	CAF97690<br>CAG04243	FRUP00000137925<br>FRUP00000137926<br>FRUP00000137928<br>FRUP00000140671
VIPR2	7434	NP_058934	NP_033537	NP_001014970	CAF97773<br>CAF98740	FRUP00000128391<br>FRUP00000158712
**Subtotal**	**48**	**44**	**46**	**42**	**53**	**50**

Class C

CASR	846	NP_058692	NP_038831	XP_416491	CAG00571	BAA26122
GABBR1	2550	NP_112290	NP_062312		CAG02742	FRUP00000145292
GPR51/GABBR2	9568	NP_113990	XP_143750	XP_419066	CAG10041	FRUP00000128923
GPRC5A/RAI3	9052	NP_001073359	NP_852109	XP_416200		
GPRC5B	51704	XP_215095	NP_071865	XP_001233984	CAG06055<br>CAG11409	FRUP00000134725<br>FRUP00000143419
GPRC5C	55890	XP_213518	NP_671750	XP_425386	CAG00236<br>CAG03050	FRUP00000130654<br>FRUP00000137392<br>FRUP00000164268
GPRC5D	55507	XP_575701	NP_444348			
GPRC6A	222545	XP_228164	NP_694711	XP_426177		FRUP00000133733<br>FRUP00000163538
GRM1	2911	NP_058707	NP_058672	XP_419652	CAG03586<br>CAG13194	FRUP00000135014<br>FRUP00000143714

Gene Name	Human Gene ID	Rat	Mouse	Chicken	*T. nigroviridis*	*T. rubripes*
GRM2	2912	XP_343471	AAO85115	XP_425148	CAG06804<br>CAG06963	FRUP00000141550<br>FRUP00000149314
GRM3	2913	XP_001069653	NP_862898	XP_416842	CAG13037	FRUP00000129944
GRM4	2914	NP_073157	XP_196179	XP_418031	CAG04030	FRUP00000150481
GRM5	2915	NP_058708	XP_149971	NP_989469	CAG00460	FRUP00000140545<br>FRUP00000140546
GRM6	2916	NP_075209	NP_775548	XP_428614	CAF89911	FRUP00000164837
GRM7	2917	NP_112302	NP_796302	XP_414442	CAF96886<br>CAG11283	FRUP00000132600<br>FRUP00000164838
GRM8	2918	NP_071538	NP_032200	XP_425426	CAG01066<br>CAG12082	FRUP00000147943<br>FRUP00000150688
**Subtotal**	**16**	**16**	**16**	**14**	**19**	**23**

Class F

FZD1	8321	NP_067089	NP_067432	XP_418648		
FZD2	2535	NP_742032	NP_065256	NP_989553	CAG02812	FRUP00000148265
FZD3	7976	NP_703204	NP_067433	XP_420029	CAG00137<br>CAG11035	FRUP00000137946<br>FRUP00000150170
FZD4	8322	NP_072145	NP_032081	NP_989430	CAF96654	FRUP00000140126
FZD5	7855	NP_776210	NP_073558	XP_426568	CAF94116	FRUP00000135762
FZD6	8323	XP_343231	NP_032082	XP_418372	CAF98078	FRUP00000130766
FZD7	8324	XP_237191	NP_032083	NP_989552	CAF91294<br>CAG01343	FRUP00000143203
FZD8	8325	XP_344617	NP_032084	XP_418566	CAF97764<br>CAF98771	FRUP00000148131<br>FRUP00000159741<br>scaffold_122<br>scaffold_1549
FZD9	8326	NP_695217	XP_284144	XP_425392	CAF91428	FRUP00000142700
FZD10	11211	ENSRNOT00000029702	NP_780493	NP_989429	CAF94498	FRUP00000142418
SMO	6608	NP_036939	NP_795970	XP_414970	CAF97226	FRUP00000141346
**Subtotal**	**11**	**11**	**11**	**11**	**13**	**14**
