# Supplementary material for: GPCR Genes Are Preferentially Retained after Whole Genome Duplication
Source: PLoS One. 2008 Apr 2;3(4):e1903. doi: 10.1371/journal.pone.0001903 (PMC2270905; doi:10.1371/journal.pone.0001903)
Supplement: Table S2 — A. Inventory of nGPCR genes in the MRCA of tetrapods and teleosts, and the number of derived nGPCR genes in human, rat, mouse, chicken, T. nigroviridis, and T. rubripes. The MRCA for each family of orthologous nGPCRs is defined as the gene that gave rise to a group of orthologs or co-orthologs in modern species. Each data point represents the total number of genes belonging to a select class or subclass of nGPCRs. The orthologous relationships of nGPCRs from different species are deduced by syntenic mapping and phylogenetic tree building analysis. B. List of nGPCR genes wherein a distinct evolutionary path can be traced from the MRCA of tetrapods and teleosts to modern species. The accession number of individual nGPCRs in each species is listed according to their classification. For human nGPCRs, the gene ID also is provided. Ancestral nGPCRs are denoted by the name of the human ortholog(s). C. List of nGPCR genes for which an ancestral form in the MRCA of tetrapods and teleosts cannot be defined. D. Correlation coefficients of nGPCR inventories between pairs of species. E. Number of singleton and duplicated nGPCR genes in model vertebrates, and number of nGPCR families with gene duplicates in each species. (0.08 MB PDF) [file pone.0001903.s004.pdf]

Table S2 A. Inventory of nGPCR genes in the MRCA of tetrapods and teleosts, and the number of derived nGPCR genes in model vertebrates.

Class	MRCA	Human	Rat	Mouse	Chicken	*T. nigroviridis*	*T. rubripes*
**A1**	19	19	16	18	21	40	41
**A2**	42	52	55	56	52	83	79
**A3**	21	26	26	26	18	30	30
**A4**	34	46	40	43	39	46	46
**A5**	38	46	44	45	46	61	69
**A6**	7	8	8	8	8	10	10
**A7**	46	62	61	65	46	70	68
**Subtotal**	**207**	**259**	**250**	**261**	**230**	**340**	**343**
**B**	38	46	42	44	41	53	50
**C**	14	16	16	16	14	19	23
**F**	10	11	11	11	11	13	14
**Total**	**269**	**332**	**319**	**332**	**296**	**425**	**430**

Table S2 B. List of nGPCR genes wherein a distinct evolutionary path can be traced from the MRCA of tetrapods and teleosts to modern species.

Class A

Subclass A1

MRCA	Human gene ID; accession number	Rat	Mouse	Chicken	*T. nigroviridis*	*T . rubripes*
GPR26	2849; NP 703143	NP_620196	NP_775586	XP_421809	CAG00716<br>CAG03282	FRUP00000148603<br>FRUP00000160817
GPR50	9248; NP 004215	XP_346375	NP_034470	NP_990692	CAF99125	FRUP00000153084
GPR61	83873; NP 114142	XP_227581	NP_780679	NW_060850	CAG06826<br>CAG07504	
GPR62	118442; NP 543141	XP_576464	XP_488192		CAG06806<br>CAG06960	FRUP00000149305
GPR78	27201; NP 543009			XP_426354	GSTENT00035915001<br>CAG05074	FRUP00000132645<br>FRUP00000158903
GPR84	53831; NP 065103	GeneID_367000	NP_109645		CAG06758	FRUP00000162610
GPR101	83550; NP 473362	XP_229186	XP_141764		CAG09823<br>SCAF13862	FRUP00000144472
GPR135	64582; NP 072093	NP_861436	NP_861417	XP_426475	CAF98168	FRUP00000151143
MTNR1A	4543; NP 005949	XP_341442	NP_032665	NP_990693	CAF96594<br>CAG04524	FRUP00000158716<br>FRUP00000160445
MTNR1B	4544; NP 005950	XP_345900	NP_663758	XP_417201	SCAF14601	FRUP00000141583
OPN1LW	5956; NP 064445		GeneID_20164	NP 990771<br>NP 990740	CAF96877	FRUP00000164823
OPN1SW	611; NP 001699	NP_112277	NP_031564		CAF96876	FRUP00000164822
OPN3	23596; NP 055137		NP_034228	XP 426139<br>XP 425584	CAF89728<br>CAF97626<br>CAG08854<br>CAG09827	FRUP00000131360<br>FRUP00000140941<br>FRUP00000143705<br>FRUP00000155686
OPN4	94233; NP 150598	NP_620215	NP_038915	XP 421494<br>NP 989956	CAF90276<br>CAF96662<br>CAF99228<br>CAG08413	FRUP00000141781<br>FRUP00000141782<br>FRUP00000145811<br>FRUP00000145812<br>FRUP00000149714<br>FRUP00000163792
OPN5	221391; NP 859528	NP_861437	NP_861418	XP 426228<br>XP 420056<br>XP 419178	CAF91597<br>CAG00077<br>CAG03922<br>CAG09534<br>CAG11258<br>CAG13006	FRUP00000133622<br>FRUP00000139290<br>FRUP00000145353<br>FRUP00000149081<br>FRUP00000154669<br>FRUP00000162507<br>FRUP00000164504<br>FRUP00000165286
RE2	23432; NP 031395	XP_222837	XP_136361	XP_416594	CAG05829<br>CAG09451	FRUP00000138055<br>FRUP00000148240
RGR	5995; NP 002912	XP_224673	NP_067315	XP_421504	CAF98663<br>CAG13313	FRUP00000142438<br>FRUP00000148015
RHO	6010; NP 000530	NP_254276	NP_663358	XP 414454<br>NP 990821	CAG06941<br>CAG11315<br>CAG11334	FRUP00000136321<br>FRUP00000136323<br>FRUP00000156773<br>scaffold 200
RRH	10692; NP 006574	XP_227718	NP_033128	XP_420649	CAF94915	FRUP00000130657

Subtotal

19	19	16	18	21	40	41

Subclass A2

ADORA1/ADORA3	134; NP 000665<br>140; NP 000668	NP 058851<br>NP 037028	NP 001008533<br>NP 033761	NP 989647<br>NP 989482	CAF96797<br>CAG07486<br>CAG11815<br>CAG11816	FRUP00000128561<br>FRUP00000143490<br>FRUP00000149785
ADORA2A	135; NP 000666	NP 445746	NP 033760	XP 425280	CAF93754<br>CAG02468	FRUP00000141222<br>FRUP00000142941<br>FRUP00000144716
ADORA2B	136; NP 000667	NP 058857	NP 031439	NP 990418	CAF98510<br>CAG09178	FRUP00000154463
ADRA1B/GPR88	147; NP 000670<br>54112; NP 071332	NP 058687<br>NP 113884	NP 031442<br>NP 071872	XP 414483<br>XP 001232659	CAF92745<br>CAF97256	FRUP00000152462<br>FRUP00000164690
ADRA1A	148; NP 000671	NP 058887	NP 038489	XP 425762	CAG11192	FRUP00000141240

MRCA	Human gene ID; accession number	Rat	Mouse	Chicken	*T. nigroviridis*	*T . rubripes*
ADRA2A	150; NP 000672	NP 036871	NP 031443	XP 426537	CAG01293<br>CAG05052<br>CAG05356	FRUP00000127209<br>FRUP00000145339<br>FRUP00000154128
ADRA2B	151; NP 000673	NP 612514	NP 033763		CAG07800	FRUP00000129228
ADRA2C	152; NP 000674	NP 612515	NP 031444	XP 426355<br>XP 425203	CAG00720<br>CAG03287	FRUP00000136842<br>FRUP00000158905
ADRA1D	146; NP 000669	NP 077809	NP 038488	XP 420871	CAF91280	FRUP00000140656
ADRB1/ADRB3	153; NP 000675<br>155; NP 000016	NP 036833<br>NP 037240	NP 031445<br>NP 038490	XP 426540<br>XP 428541	CAG08867<br>CAG12607	FRUP00000128218<br>FRUP00000147601
ADRB2	154; NP 000015	NP 036624	NP 031446	XP 425195	CAG10129	FRUP00000128843<br>FRUP00000157526
CHRM1	1128; NP 000729	NP 542951	NP 031724		CAG10670	FRUP00000131048
CHRM2	1129; NP 000730	NP 112278	NP 987076	XP 416359	CAG02408<br>CAG03403	FRUP00000127975<br>FRUP00000129486
CHRM3	1131; NP 000731	NP 036659	NP 150372	NP 990730	CAF92462<br>CAG10073	FRUP00000160548<br>scaffold 143
CHRM4	1132; NP 000732	XP 345404	NP 031725	XP 421119	CAG05625<br>CAG06610	FRUP00000147533<br>FRUP00000155473
CHRM5	1133; NP 036257	NP 059058	NP 991352	XP 426437	CAG03529	FRUP00000130167
DRD1	1812; NP 000785	NP 037269	NP 031610	XP 425206	CAF93510<br>CAF95884<br>CAG11724	FRUP00000154522<br>FRUP00000164051<br>FRUP00000164859
DRD2	1813; NP 000786	NP 036679	NP 034207	XP 425811	CAF92229<br>CAF97490<br>CAG13001	FRUP00000129665<br>FRUP00000143523<br>FRUP00000153968
DRD3	1814; NP 387512	NP 058836	NP 031903	XP 416570	CAG04235	scaffold 146
DRD4	1815; NP 000788	NP 037076	NP 031904	XP 420947	CAF95731<br>CAF98376	FRUP00000142755<br>FRUP00000149812
DRD5	1816; NP 000789	NP 036900	NP 038531	XP 426518<br>XP 426351	CAF90994<br>CAG02007	FRUP00000131890<br>FRUP00000138676
GPR21	2844; NP 005285	XP 231251	NP 796357	XP 415391	CAF93159<br>CAG09652	FRUP00000163990
GPR22	2845; NP 005286	XP 234041	NP 780400	XP 001231969	CAF89181<br>CAF96769<br>CAG02014<br>CAG08711	FRUP00000133859<br>FRUP00000162183<br>FRUP00000163060<br>scaffold 374
GPR52	9293; NP 005675	NW 047398	XP 890263	NP 001012941	CAG00742	FRUP00000163527
GPR58	9287; NP 055441	NP 001008512	NP 001007267	XP 427716	CAF94065	FRUP00000131967
GPR75	10936; NP 006785	XP 573685	NP 780699	XP 426099	SCAF15006	scaffold 905
GPR119	139760; NP 848566	NP 861435	NP 861416	XP 426720<br>XP 426248	CAF97243	FRUP00000143792
HRH1	3269; NP 000852	NP 058714	NP 032311	XP 425153	CAG07054	FRUP00000164084
HRH2	3274; NP 071640	NP 037097	NP 001010973	XP 425208	CAG01551<br>CAG04750	FRUP00000131597<br>FRUP00000138905
HRH4/HRH3	59340; NP 067637<br>11255; NP 009163	NP 571984<br>NP 445958	NP 694727<br>NP 598610	XP 426079<br>XP 414481<br>XP 425705<br>XP 425117	CAG07613<br>CAG10949	FRUP00000146131<br>FRUP00000165198
HTR1A	3350; NP 000515	NP 036717	NP 032334	XP 429136	CAF91711<br>CAF93441	FRUP00000136865<br>FRUP00000154633
HTR1B	3351; NP 000854	NP 071561	NP 034612	XP 419875	CAF89927	FRUP00000161510
HTR1D	3352; NP 000855	NP 036984	NP 032335	XP 001232312	CAG10464	FRUP00000127520
HTR1F/HTR1E	3354; NP 000856<br>3355; NP 000857	NP 068629	NP 032336	XP 425535<br>XP 001235179	CAG09227	FRUP00000155421
HTR2B	3357; NP 000858	NP 058946	NP 032337	AAF20211	CAC85912<br>CAC86247	FRUP00000147089

MRCA	Human gene ID; accession number	Rat	Mouse	Chicken	*T. nigroviridis*	*T . rubripes*
HTR2C/HTR2A	3356; NP 000612<br>3358; NP 000859	NP 058950<br>NP 036897	NP 766400<br>NP 032338	XP 425628<br>XP 426265	CAG12257	FRUP00000135888
HTR4/GPR160	26996; NP 055188<br>3360; NP 000861	NP 001020318<br>NP 036985	XP 130823<br>NP 032339	XP 422799	CAF93600<br>CAF95370<br>CAG04907	FRUP00000138443<br>FRUP00000140658<br>FRUP00000164424
HTR5A/HTR5B	3361; NP 076917	XP 341112<br>NP 037280	NP 034613<br>NP 032340	XP 426604<br>XP 425970	CAG04298	scaffold 934
HTR6	3362; NP 000862	NP 077341	NP 067333	XP 427576	CAG01704	FRUP00000139170<br>FRUP00000139171
HTR7	3363; NP 062873	NP 075227	NP 032341	XP 421666<br>XP 420880	CAG05134<br>CAG09680	FRUP00000139991<br>FRUP00000144402<br>FRUP00000144993
TRAR1/GPR57q/PNRe	9288; NC 000006<br>9038; NP 003958<br>134864; NP 612200	NP 001009532<br>NP 001009650<br>NP 599155<br>NP 783173	NP 001008429<br>NP 001009574<br>NP 038736<br>NP 444435	XP 419740	CAF91012<br>CAF92549<br>CAF92550<br>CAF92551<br>CAF93538<br>CAF93617<br>CAF93618<br>CAF94841<br>CAF95185<br>CAF95186<br>CAF95187<br>CAF96106<br>CAG13003	FRUP00000131749<br>FRUP00000138381<br>FRUP00000150777<br>FRUP00000150779<br>FRUP00000154655<br>FRUP00000157293<br>scaffold 2245
TRAR4/TRAR5	319100; NP 778237<br>83551; NP 444508	NP 783174<br>NP 783189<br>NP 783191<br>NP 783190	NP 001010828<br>NP 001008499<br>NP 001010830<br>NP 001010840<br>NP_001010837			FRUP00000157578<br>FRUP00000135638

Subtotal

**42**	**52**	**55**	**56**	**52**	**83**	**79**

Subclass A3

CNR1	1268; NP 149421	NP_036916	NP_031752	XP_426191	CAG09211<br>CAG10478	FRUP00000157187<br>scaffold 1709
CNR2	1269; NP 001832	NP_065418	NP_034054	XP_425791	CAF91752	FRUP00000161224
EDG1	1901; NP 001391	NP_058997	NP_031927	XP_422305	CAF90490	scaffold 6096
EDG2	1902; NP 476500	NP_446388	NP_034466	XP_424918	CAF89669<br>CAF90900<br>CAF92197	FRUP00000154449<br>scaffold 86
EDG3/EDG6	1903; NP 005217<br>8698; NP 003766	XP 225216<br>XP 234930	NP 034231<br>NP 034232	XP 428818<br>XP 428050	CAG07943<br>CAG12442	FRUP00000154122<br>scaffold 270<br>scaffold 270<br>scaffold 203
EDG4	9170; NP 004711	XP_573887	NP_064412	XP_423763		scaffold 799
EDG5	9294; NP 004221	NP_058888	NP_034463		SCAF15050<br>chr3 11567980 8876	scaffold 180
EDG7	23566; NP 036284	NP_076459	NP_075359	XP_422368	CAG08072	FRUP00000148649
EDG8	53637; NP 110387	NP_068543	NP_444420		CAF92590	FRUP00000139298
GPR6	2830; NP 005275	NP_113994	NP_951013	XP_426182	CAG03572	FRUP00000134973
GPR12/GPR3	2835; NP 005279<br>2827; NP 005272	NP 110458<br>NP 714949	NP 001010941<br>NP 032180	XP_425637	CAG04221<br>CAG12255<br>CAF88618	FRUP00000158473<br>FRUP00000160201
MC1R	4157; NP 002377	EDL92809	NP_032585	XP_425135	AAQ55176	AAO65548
MC2R	4158; NP 000520	XP_574167	NP_032586	XP_426070	CAF95601	
MC4R	4160; NP 005903	NP_037231	NP_058673	XP_426042	AAQ55178	AAO65551
MC5R/MC3R	4161; NP 005904<br>4159; NP 063941	NP 037314<br>NP 001020441	NP 038624<br>NP 032587	NP 001026186	AAQ55179	AAO65553
PTGER2/PTGDR	5729; NP 000944<br>5732; NP 000947	NP 071577<br>NP 112350	NP 032988<br>NP 032990	XP_426485	CAG00989	scaffold 1339
PTGER3	5733; NP 942008	NP_036836	NP_035326	XP_426672	CAG01184	FRUP00000131256
PTGER4	5734; NP 000949	NP_114465	NP_032991	XP_424770	CAF95032<br>CAF96390<br>CAF97110<br>CAG09586	FRUP00000155267<br>FRUP00000157769<br>FRUP00000161139<br>FRUP00000162687

MRCA	Human gene ID; accession number	Rat	Mouse	Chicken	*T. nigroviridis*	*T . rubripes*
PTGFR/PTGER1	5731; NP 000946<br>5737; NP 000950	NP 037232<br>NP 037247	NP 038669<br>NP 032992	NP_001038122	CAG03303	FRUP00000149163<br>FRUP00000151130
PTGIR	5739; NP 000951	XP_218457	NP_032993		SCAF14537	FRUP00000130879
TBXA2R	6915; NP 001051	NP_058750	NP_033351		CAG00626	FRUP00000152568
**Subtotal**						
**21**	**26**	**26**	**26**	**18**	**30**	**30**

Subclass A4

ADMR	11318; NP 009195	NP_445754	NP_031438		CAF90054	FRUP00000146637
CYSLTR1/CYSLTR2	10800; NP 006630<br>57105; NP 065110	NP 446093<br>NP 596904	NP 067451<br>NP 598481	XP 426249<br>XP 425629	SCAF7335	FRUP00000150747
EBI2	1880; NP 004942		NP_898852	XP_428804	CAG07299<br>chr2 7825313-77092	FRUP00000150142<br>FRUP00000161432
F2R	2149; NP 001983	NP_037082	NP_034299	XP_424799	CAG07882	FRUP00000152221
F2RL1	2150; NP 005233	NP_446349	NP_032000	NP_001012626	CAG05264<br>CAG05265	FRUP00000130845<br>FRUP00000130847
F2RL2	2151; NP 004092	NP_445765	NP_034300	XP_414523	CAG05266<br>CAG07880	FRUP00000151261<br>FRUP00000129585<br>FRUP00000130849
F2RL3	9002; NP 003941	NP_446260	NP_032001	XP_418256	CAF95426	chrUn_311834154-530
GPR4/GPR132	29933; NP 037477<br>2828; NP 005273	XP 234574<br>NP 001020851	NP 064309<br>NP 783599	XP_426469	CAF97465<br>CAG12733	FRUP00000128368<br>FRUP00000135821
GPR17	2840; NP 005282	NW_047510	AAH70439		CAG04290<br>CAG05650	FRUP00000128875<br>FRUP00000147658
GPR18	2841; NP 005283		NP_877958	NW_060236	CAG07300	FRUP00000150143
GPR20	2843; NP 005284	NP_071552	NP_775541	XP_425941	CAF99361<br>CAG11945	FRUP00000136628<br>FRUP00000165047
GPR23/GPR35/GPR55/GPR92	2846; NP 005287<br>2859; NP 005292<br>9290; NP 005674<br>57121; NP 065133	XP 228501<br>NP 001032436<br>AAD22411<br>XP 575667	NP 780480<br>NP 071715<br>NP 001028462<br>XP 355812	XP 425741<br>XP 420150<br>XP 422645<br>XP 416504	CAF90112<br>CAG03809	FRUP00000150750
GPR34	2857; NP 005291	NP_001020096	NP_035953	XP_420190	SCAF14978	scaffold_1642
GPR41/GPR42a	2865; NP 005295<br>2866; NP 005296	XP_344881	XP_145470		CAF89689	FRUP00000153029
GPR43/GPR40	2864; NP 005294<br>2867; NP 005297	NP 695216<br>NP 001005877	NP 918946<br>NP 666299	XP 428780<br>XP 426943	CAF93280<br>CAG03362	FRUP00000129763<br>FRUP00000161234
GPR65	8477; NP 003599	XP_234367	NP_032178	XP_421305	SCAF15019	FRUP00000150188
GPR68	8111; NP 003476	XP_234483	NP_780702	XP_426459	CAF99011	FRUP00000136318
GPR80	27199; NP 543008	NP_997471	NP_001001490	XP 425595<br>XP 425594	CAG04953	FRUP00000159316
GPR86	53829; NP 076403	NP_001002853	NP_083084		CAF97388	scaffold_239
GPR91	56670; NP 149039	XP_579061	NP_115776	XP_422836	CAF97381	FRUP00000138067
GPR105	9934; NP 055694	NP_598261	NP_573463	XP_422841	SCAF14677	
GPR109A/GPR31/GPR81	338442; NP 808219<br>2853; NP 005290<br>27198; NP 115943	NP 852141<br>XP 217867<br>XP 001072594	NP 109626<br>GeneID 107431<br>NP 780729		CAF90987<br>CAG05298<br>CAG08446	FRUP00000136429<br>FRUP00000146889<br>FRUP00000148806<br>FRUP00000150723
GPR141	353345; NP 861456	NP_861432	NP 861419	XP_418832	SCAF14544	scaffold_240
GPR174/P2RY10	84636; NP 115942<br>27334; NP 938147	XP 228495<br>XP 228500	NP 001028423<br>NP 766023	NP 001008464<br>XP 420149<br>XP 420148	CAG14148	scaffold_1393
P2RY1	5028; NP 002554	NP_036932	NP_032798	XP 427676<br>NP 990664	CAF97378<br>CAG03842	FRUP00000138058
P2RY2	5029; NP 788085	NP_058951	NP_032799	XP_425667	CAG00917<br>CAF97358	FRUP00000127686<br>FRUP00000141264<br>FRUP00000165330
P2RY4	5030; NP 002556	NP_113868	NP_065646	XP_420157	CAG09809	FRUP00000163721
P2Y5	10161; NP 005758	NW_047454	NP_780325	NP_990530	CAG02919<br>CAG12785	FRUP00000141960
P2RY6	5031; NP 004145	NP_476465	NP_898991	NP_990526	CAG00913	FRUP00000151445

MRCA	Human gene ID; accession number	Rat	Mouse	Chicken	*T. nigroviridis*	*T . rubripes*
P2RY8	286530; NP 835230			NP_001008679		FRUP00000139369
P2RY11	5032; NP 002557				CAG12186	FRUP00000147765
P2RY12/GPR171/GPR87	29909; NP 037440<br>53836; NP 076404<br>64805; NP 795345	XP 227177<br>NP 073637	NP 775574<br>NP 115775<br>NP 081847	XP 422842<br>XP 422840<br>XP 422839	CAF97387	FRUP00000138083
PTAFR	5724; NP 000943	NP_445773	XP_357441	XP_425768	CAG10424	FRUP00000134471
RDC1	57007; NP 064707	NP_445804	NP_031748	XP_426554	CAG05877	scaffold_387
**Subtotal**						
**34**	**46**	**40**	**43**	**39**	**46**	**46**

Subclass A5

AVPR1A	552; NP 000697	NP_444178	NP_058543	XP_425436	CAF96006<br>CAG12390	FRUP00000141525<br>FRUP00000161762
AVPR1B	553; NP 000698	NP_058901	NP_036054	XP 425822<br>XP 425434	CAF96931	scaffold_8423
AVPR2	554; NP 000045	NP_062009	NP_062277		CAG03798<br>CAG07446	FRUP00000161883<br>scaffold 323
CCKAR/CCKBR	886; NP 000721<br>887; NP 795344	NP 036820<br>NP 037297	NP 033957<br>NP 031653	XP 420751<br>NP 001001742	CAG04576	FRUP00000133099
EDNRA	1909; NP 001948	NP_036682	NP_034462	NP_989450	CAG02202<br>CAG11066	FRUP00000133802<br>FRUP00000133804<br>FRUP00000133807<br>FRUP00000157593<br>FRUP00000157598
EDNRB	1910; NP 003982	NP_059029	NP_031930	XP 417001<br>NP 989451	CAF90114<br>CAF97649<br>CAG05905	FRUP00000141004<br>FRUP00000141006<br>FRUP00000150746<br>FRUP00000163212
FSHR	2492; NP 000136	NP_954707	NP_038551	NP_990410	CAF91019	scaffold 49
GNRHR2	114814; NP 476504			NP_001012627	CAF92557<br>CAF93245<br>CAG00826<br>CAG03087<br>CAG13098	FRUP00000134302<br>FRUP00000138360<br>FRUP00000141711<br>FRUP00000156324<br>FRUP00000160897
GPR	11245; NP 009154	XP_342494	NP_958755	XP_426435	CAG03525	FRUP00000131045
GPR10	2834; NP_004239	NP_631932	NP_963909	XP_428805	CAF97466	FRUP00000135820
GPR19	2842; NP 006134	NP_542146	NP_032183	XP_428465	CAF90680	FRUP00000131864
GPR37	2861; NP 005293	NP_476549	NP_034468	XP_415992	CAG01068	FRUP00000137022<br>FRUP00000140117<br>scaffold 2384<br>scaffold 3299
GPR37L	9283; NP 004758	XP_573457	NP_602320	XP_419256	SCAF7416	FRUP00000136126<br>FRUP00000142342
GPR45	11250; NP 009158	XP_237112	NP_444337		CAG05802	FRUP00000163802
GPR63	81491; NP 110411	XP_232847	NP_109658	XP_426189	CAF93484	FRUP00000149653
GPR73/GPR73L1	10887; NP 620414<br>128674; NP 658986	NP 620433<br>NP 620434	NP 067356<br>NP 659193	XP 419333<br>XP 419334	CAF98618	FRUP00000139538<br>FRUP00000154172
GPR74	10886; NP 444264	NP_076470	NP_573455	XP_426315	CAF97104<br>CAG07773	FRUP00000155275<br>FRUP00000155634
GPR83	10888; NP 057624	NP 536336	NP 034417	XP 426254<br>XP 425651	CAG06215	FRUP00000164362
GPR85	54329; NP 061843	NP_071590	NP_659503	XP_416019	CAG12125	FRUP00000143116
GPR103	84109; NP 937822	NP_937842	NP_780733	XP_420626	CAG04805<br>SCAF14750	FRUP00000142516
GPR173/GPR27	54328; NP 061842<br>2850; NP 061844	NP 071591<br>NP 075587	NP 081819<br>NP 032184	XP_414427	CAF93024<br>CAF96921<br>CAG03946	FRUP00000150812<br>FRUP00000153190<br>FRUP00000155856
GRPR/BRS3	680; NP 001718<br>2925; NP 005305	NP 690058<br>NP 036838	NP 033896<br>NP 032203	NP 989737<br>NP 989738	CAF99824<br>CAG05835	FRUP00000156510<br>FRUP00000161978
HCRTR2/HCRTR1	3061; NP 001516<br>3062; NP 001517	NP 037196<br>NP 037206	NP 945197<br>NP 945200	NP 001019755	CAG09420	FRUP00000163192
LGR4	55366; NP 060960	NP_775450	XP_355385	XP_426162	CAF99699	scaffold_826
LGR5	8549; NP 003658	XP_235149	NP_034325	XP_425441	CAG14806	FRUP00000149831

MRCA	Human gene ID; accession number	Rat	Mouse	Chicken	*T. nigroviridis*	*T . rubripes*
LGR6	59352; NP 067649	XP_573455	XP_283647	XP_419253	CAG07358	FRUP00000148236<br>FRUP00000148237
LGR7	59350; NP 067647	NP_958820	NP_997617	XP_426253	CAG04641	scaffold_1619
LGR8	122042; NP 570718	XP_344074	NP_569720		CAG00416	FRUP00000159568
LHCGR	3973; NP 000224	NP_037110	NP_038610	NP_990267	CAF91017<br>CAG09466	
NMBR	4829; NP 002502	NP_036931	NP_032729	XP_426167	CAF94959<br>CAG14163	FRUP00000150118<br>GSTENT00011415001 pr
NPFFR1	64106; NP 071429	NP_071627	XP_137119	NP_989693	CAG09719	FRUP00000157824
NPY2R	4887; NP 000901	NP_076458	NP_032757	XP 420373<br>XP 414599	CAF96589<br>SCAF14573	FRUP00000138240<br>DAA05226
NPY5R/NPY1R/NPY6R	4886; NP 000900<br>4889; NP 006165<br>4888; NC 000005	NP 001013050<br>NP 037001<br>NP 037003	NP 035064<br>NP 057917<br>NP 035065	XP 426285<br>XP 420388	CAG02528<br>CAG05410	FRUP00000160782<br>FRUP00000160783
OXTR	5021; NP 000907		XP_144956	XP_426768	CAG06809<br>CAG06958	FRUP00000127611<br>FRUP00000149294
PPYR1	5540; NP 005963	NP_113769	NP_032945	XP_426511	CAG09405	FRUP00000164394
TACR1	6869; NP 056542	NP_036799	NP_033339	NP_990199	CAG05392<br>CAG12579	FRUP00000138587<br>FRUP00000148382<br>FRUP00000160726
TACR3/TACR2	6865; NP 001048<br>6870; NP 001050	NP 542946<br>NP 058749	NP 033340<br>NP 067357	XP 426496<br>XP 001232174	CAG01274<br>CAG05682<br>CAG11520	FRUP00000132595<br>FRUP00000135143<br>FRUP00000147917
TSHR	7253; NP 000360	NP_037020	NP_035778	XP_426455	CAG00111	FRUP00000145651
**Subtotal**						
**38**	**46**	**44**	**45**	**46**	**61**	**69**

Subclass A6

GHSR	2693; NP 004113	NP_114464	NP_796304	NP_989725	CAF95899	FRUP00000131849
GPR38	2862; NP 001498			XP_425630	CAG12790	FRUP00000138615
GPR39	2863; NP 001499	DAA06056	AAH85285	XP_422128	CAG11238	FRUP00000135301
NMUR1	10316; NP 006047	NP_075588	NP_034471	XP_426705	CAF97543<br>CAG03466	FRUP00000153653<br>FRUP00000156953
NMUR2	56923; NP 064552	NP_071611	NP_694719	XP_425209	CAG03508	FRUP00000147487
NTSR1/NTSR2	4923; NP 002522<br>23620; NP 036476	XP 345485<br>NP 073186	NP 061236<br>NP 032773	XP_425707	CAF89857	FRUP00000128303
TRHR	7201; NP 003292	NP 037179<br>NP 852029	NP 038724<br>NP 573465	XP 425702<br>NP 990261	CAF92799<br>CAF96270<br>CAG03888	FRUP00000134073<br>FRUP00000146669<br>FRUP00000153993
**Subtotal**						
**7**	**8**	**8**	**8**	**8**	**10**	**10**

Subclass A7

AGTR1	185; NP 114438	NP_112271	NP 796296<br>NP 780295	NP_990488	CAG04270	FRUP00000150672
AGTR2	186; NP 000677	NP_036626	NP_031455	XP_426266	CAG08373	scaffold 1395
AGTRL1	187; NP 005152	NP_112639	NP_035914	XP_425289	CAF90289<br>CAF99292<br>SCAF12244	FRUP00000134029<br>FRUP00000134030<br>FRUP00000146396<br>FRUP00000165603
BDKRB1	623; NP_000701	NP_110478	NP_031565	NP_001074189	CAG10408	FRUP00000150183<br>FRUP00000150187
BLR1	643; NP_116743	NP_445755	NP_031577	NP_001026083	CAG09055	FRUP00000156705
CXCR3	2833; NP 001495	NP_445867	NP_034040		CAF98051<br>CAF98053<br>chr8_854312-855332	FRUP00000138635<br>FRUP00000138636<br>chrUn:212102004-3119
CX3CR1	1524; NP_001328	NP_598218	NP_034117	XP_418820	chr8_3994467_940	chrUn_197062813_3289
CCR1/CCR2/CCR3/CCR4/CCR5	1230; NP 001286<br>1231; NP 000639<br>1232; NP 847899<br>1233; NP 005499<br>1234; NP 000570	NP_065417<br>XP_236742<br>NP_068638<br>NP_446410<br>NP_598216<br>NP_446412	NP_034042<br>NP_034045<br>NP_034044<br>NP_034046<br>NP_034047<br>NP_031744	XP_426017<br>XP_418796<br>XP_418795	chr8_3997729_3998596	FRUP00000127130

MRCA	Human gene ID; accession number	Rat	Mouse	Chicken	*T. nigroviridis*	*T . rubripes*
CCR6	1235; NP 004358	NP_001013163	NP_033965	XP_419608	CAF92962	FRUP00000146532
CCR7	1236; NP 001829	NP_955783	NP_031745	XP_425875	CAG06152	FRUP00000154746
CCR9	10803; NP 006632	NP_758832	NP_034043	XP_426014	CAF90274<br>CAG05508	FRUP00000142615
CMKLR1/GPR33	1240; NP 004063<br>2856; NC 000014	NP 071554<br>NP 001026993	NP 032179<br>NP 032185	XP 425239<br>XP 423260	CAF94854<br>CAF94856<br>CAG08499	FRUP00000145765<br>FRUP00000149103<br>FRUP00000155117
CCR10	2826; NP 057686	XP_343969	NP_031747		chr2_1817756-1818529	FRUP00000140298
CCR11	51554; NP 848540	Q9ESK1	NP_663746		CAF88488	FRUP00000138457
C5R1/C3AR1/GPR77	719; NP 004045<br>728; NP 001727<br>27202; NP 060955	NP 114449<br>NP 446071<br>NP 001003710	NP 033909<br>NP 031603<br>NP 795886	XP 416428<br>XP 428039	CAG02859	FRUP00000134135
CXCR4	7852; NP 003458	NP_071541	NP_034041	NP_989948	CAF97662<br>CAG01848	FRUP00000134232<br>FRUP00000137823
FPR1/FPRL1/FPRL2/GPR152/GPR32	2357; NP 002020<br>2358; NP 001453<br>2359; NP 002021<br>390212; NP 996880<br>2854; NP 001497	XP 218012<br>XP 001057995<br>XP 218016<br>XP 218022<br>XP 001073753<br>XP_001068404	NP 038549<br>NP 032068<br>XP 622087<br>EDL38017<br>AAC34587<br>AAN63620<br>NP_996856<br>AAN63621		CAG11673	scaffold 3925
GALR1	2587; NP 001471	NP_037090	NP_032108	XP 426066<br>XP 414080	CAG00818<br>CAG12842	FRUP00000149875<br>FRUP00000153302
GALR2/GALR3	8811; NP 003848<br>8484; NP 003605	NP 062045<br>NP 062046	NP 034384<br>NP 056553	XP 428968	CAG12230	FRUP00000158738
GPR1	2825; NP 005270	NP_037093	NP_666362	XP_421955		FRUP00000131794
GPR8/GPR7	2831; NP 005276<br>2832; NP 005277	NP_001014784	XP 136404	XP 419201<br>XP 425710	SCAF14682	
GPR14	2837; NP 061822	NP_065412	NP_663415	XP 425371<br>XP 425370<br>XP 425234<br>XP 416275	CAF97882<br>CAG02581<br>CAG02582<br>CAG12512	FRUP00000129229<br>FRUP00000164718<br>scaffold 2802
GPR25/GPR15	2838; NP 005281<br>2848; NP 005289	XP 221546<br>XP 344147	XP 156321<br>XP 357126	XP_425528	CAG11769	
GPR30	2852; NP 001496	NP_598257	NP_084047	XP_414765	CAG12216	FRUP00000146157
GPR44	11251; NP 004769	NP_001012070	NP_034092		CAG03720	FRUP00000149198
GPR54	84634; NP 115940	NP_076482	NP_444474		CAG06231	FRUP00000131919
GPR139	124274; XP 064062	NP_001019412	XP_146103	XP_428970	CAG11412	AAP72123
GPR142	350383; NP 861455	XP_577136	NP_861414	XP_428957	CAG03049	AAP72122
GPR145	84539; NP 115892				CAF99158	FRUP00000144995
GPR146	115330; NP 612454	XP_573364	NP_084534	XP_414764	CAG12217	scaffold 710
GPR151	134391; NP 919227	Q7TSN5	NP_853521		CAG01252<br>CAG10137	FRUP00000127188<br>scaffold 544
IL8RA	3577; NP 000625	NP_062183	NP_839972		CAF98991	FRUP00000127720
IL8RB	3579; NP 001548	NP_058879	NP_034039	XP_428206	CAF98209<br>chr2 16451193 2269	FRUP00000131991<br>FRUP00000127485
LTB4R/LTB4R2	1241; NP 858043<br>56413; NP 062813	NP 067688<br>NP 446092	NP 032545<br>NP 065236	XP 425304<br>XP 413985	CAF97025<br>CAF97026<br>SCAF14685	FRUP00000157228<br>FRUP00000159672<br>FRUP00000164123
GPR24	2847; NP 005288	NP_113946	NP_660114	XP_420129	CAF94024	FRUP00000130394
OPRD1	4985; NP 000902	NP_036749	NP_038650		CAG00040	FRUP00000153740
OPRK1	4986; NP 000903	NP_058863	NP_035141	XP_426087	CAG10221<br>CAG12280	FRUP00000142489
OPRL1	4987; NP 000904	NP_113757	NP_035142	XP_417424	CAG02324	FRUP00000132165
OPRM1	4988; NP 000905	NP_037203	NP_001034741	XP_419683	CAG10056	FRUP00000162543
RLN3R1/GPCR135/SALPR	51289; NP_057652	NP_001008311	NP_848832	XP_429217	CAF97176<br>CAG01356<br>CAG07748	FRUP00000153586<br>FRUP00000155748
RLN3R2/GPCR142/ GPR100	339403; NP_871001		NP_861538		CAF89883<br>CAF99357<br>CAF99875<br>CAF99890<br>CAF91545	FRUP00000127644<br>FRUP00000129142<br>FRUP00000136065

MRCA	Human gene ID; accession number	Rat	Mouse	Chicken	*T. nigroviridis*	*T . rubripes*
SSTR1/SSTR4	6751; NP 001040<br>6754; NP 001043	EDM03458<br>NP 037168	NP 033242<br>NP 033245	XP 426102<br>XP 001234905		scaffold 707
SSTR2	6752; NP 001041	NP 062221	NP 033243	XP 425384	CAF96985<br>CAF96986	AAL32173<br>GSCT00010055001 prot<br>scaffold 1772
SSTR3	6753; NP 001042	NP_598206	NP_033244	NP_001019754	CAF90874<br>CAG12520	scaffold 958<br>scaffold 927<br>scaffold 958
SSTR5	6755; NP 001044	NP_037014	XP_139909	XP_425241	CAG06053	
XCR1/GPR5	2829; NP 005274	XP_236740	NP_035928	XP_426012	CAG11634<br>chrUn 120676885 7499	FRUP00000159812<br>chrUn:277232531 5721<br>chrUn:164653540 7809

Subtotal

**46**	**62**	**61**	**65**	**46**	**70**	**68**

Subclass B

ADCYAP1R1	117; NP 001109	NP_598195	NP_031433	XP 425958<br>XP 418491	CAF94168<br>CAF95249<br>CAF95250<br>CAG12267<br>CAG12268	FRUP00000135842<br>FRUP00000161798<br>FRUP00000161801<br>FRUP00000161807
BAI1	575; NP 001693	XP_343261	NP_778156	XP_418415	CAF97520<br>CAG10859	FRUP00000164474
BAI2	576; NP 001694	XP_232778	NP_775094	XP_423828	CAF99436	
BAI3	577; NP 001695	XP_217367	NP_783573	XP_419892	CAG09441<br>GSTENT00030703001	FRUP00000129349<br>FRUP00000143645<br>FRUP00000143649
CR	799; NP 001733	NP_446268	NP_031614	XP_425985	CAG11122<br>CAG13459	FRUP00000144138<br>FRUP00000147908
CLR	10203; NP 005786	NP_036849	NP_061252	XP_421850	CAF94370<br>CAF98969<br>CAG01331	FRUP00000145573<br>FRUP00000148605
CD97	976; NP 510966	NP_001012164	NP_036055		CAG12162	FRUP00000150608
CELSR1	9620; NP 055061	XP_235570	NP_034016	XP_423746	CAG01167	
CELSR2	1952; NP 001399	XP_342319	NP_059088	XP 428195<br>XP 426750	CAG06842	FRUP00000162857
CELSR3	1951; NP 001398	NP_112610	NP_536685	XP_414354	CAG03262	FRUP00000139273
CRHR1	1394; NP 004373	NP_112261	NP_031788	NP_989652	CAG03022	FRUP00000135485
CRHR2	1395; NP 001874	NP_073205	NP_034083	NP_989785		scaffold_515
ELTD1	64123; XP 371262	NP_071630	NP_573485	XP_422383	CAG01189	FRUP00000131264
EMR1/EMR2/EMR3/EMR4b	2015; NP 001965<br>30817; NP 690883<br>84658; NP 693634<br>326342; XP 377506	NP_001007558	NP 034260<br>NP 631877		CAG04105	scaffold 1738
GCGR	2642; NP 000151	NP_742089	NP_032127	XP_423402	CAG01110<br>CAG03726	FRUP00000135578
GHRHR	2692; NP 000814	NP_036982	NP_001003685	XP_418490	CAF95846	FRUP00000146088<br>FRUP00000146089
GLP1R	2740; NP 002053	NP_036860	NP_067307	XP_426129	CAG04358	FRUP00000146170
GLP2R	9340; NP 004237	NP_068620	NP_783612	XP_425358	CAF96232	FRUP00000147245
GPR56	9289; NP 958932	NP_689448	NP_061370	XP_413999	CAG00694	
GPR64	10149; NP 005747	NP_852031	NP_848827	XP_416810	CAG05443<br>CAG00549	FRUP00000159461<br>FRUP00000143610
GPR97	222487; NP 740746	XP_226243	NP_766624	XP_413998		FRUP00000158285
GPR110/GPR111/GPR113/GPR115/<br>GPR116	266977; NP 079324 ;<br>222611; NP 722581<br>165082; NP 722577<br>221393; NP 722580<br>221395; NP 056049	XP 217359<br>XP 236958<br>XP 233935<br>NP 620810	NP 598537<br>XP 487485<br>XP 355587<br>XP 900079<br>XP 283438	XP 420068<br>XP 420066	CAG13000	FRUP00000150114<br>FRUP00000153997
GPR112	139378; NP 722576	XP_229201	XP_141802	XP_420232	CAG09829<br>CAG11729	FRUP00000140064<br>FRUP00000140065<br>FRUP00000159797

MRCA	Human gene ID; accession number	Rat	Mouse	Chicken	*T. nigroviridis*	*T. rubripes*
GPR114	221188; NP 722579	XP_240979	XP_356118	XP_414000	CAG00691	
GPR123	84435; NP 115798	XP_219468	NP_803420	XP_421695	CAG10157	
GPR124	25960; NP 116166	EDM09091	NP_473385	ENSGALP00000004917	CAF90106	FRUP00000146680
GPR125	166647; XP 291111	XP_223485	XP_132089	XP_420763	CAF90021	FRUP00000153701
GPR126/GPR128	57211; NP 065188<br>84873; NP 116176	XP 218313<br>XP 221527	NP 001002268<br>NP 766413	XP_419712	CAF94956	FRUP00000157379
GPR133	283383; NP 942122	XP_001078502	NP_001074811	XP_415094		FRUP00000137738
GPR144	347088; NP 872417	XP_578117	XP_355327	XP_415385	CAG01306	scaffold_1674
LPHN1	22859; NP 055736	NP_599235	NP_851382	XP_422382	CAG06092<br>chr3 11032320 65518	
LPHN2	23266; NP 036434	NP_075251	NP_001074767	XP 422209<br>XP 422381	CAF95119<br>CAF98480	
LPHN3	23284; NP 056051	NP_570835	NP_941991	XP_420575	CAF93446<br>CAG02284	
PTHR1	5745; NP 000307	NP_064458	NP_035329	XP_425837	CAF98426<br>CAG12650	FRUP00000144315<br>FRUP00000144316<br>FRUP00000162177
PTHR2	5746; NP 005039	NP_112351	NP_644676	XP_418507	CAF97204	FRUP00000128587<br>FRUP00000128588
VIPR1	7433; NP 004615	NP_036817	NP_035833	XP 418492<br>NP 001006328	CAF97690<br>CAG04243	FRUP00000137925<br>FRUP00000137926<br>FRUP00000137928<br>FRUP00000140671
VIPR2	7434; NP 003373	NP_058934	NP_033537	NP_001014970	CAF97773<br>CAF98740	FRUP00000128391<br>FRUP00000158712
VLGR1	84059; NP 115495	XP_342370	XP_358310	XP_429120	CAG00389	FRUP00000164895<br>FRUP00000164896
**Subtotal**						
**38**	**46**	**42**	**44**	**41**	**53**	**50**

Subclass C

CASR	846; NP 000379	NP_058692	NP_038831	XP_416491	CAG00571	BAA26122
GABBR1	2550; NP 068703	NP_112290	NP_062312		CAG02742	FRUP00000145292
GPRC5B/GPRC5A/GPRC5D	9052; NP 003970<br>51704; NP 057319<br>55507; NP 061124	NP 001073359<br>XP 215095<br>XP 575701	NP 852109<br>NP 071865<br>NP 444348	XP 416200<br>XP 001233984	CAG06055<br>CAG11409	FRUP00000134725<br>FRUP00000143419
GPRC5C	55890; NP 061123	XP_213518	NP_671750	XP_425386	CAG00236<br>CAG03050	FRUP00000130654<br>FRUP00000137392<br>FRUP00000164268
GPRC6A	222545; NP 683766	XP_228164	NP_694711	XP_426177		FRUP00000133733<br>FRUP00000163538
GPR51	9568; NP 005449	NP_113990	XP_143750	XP_419066	CAG10041	FRUP00000128923
GRM1	2911; NP 000829	NP_058707	NP_058672	XP_419652	CAG03586<br>CAG13194	FRUP00000135014<br>FRUP00000143714
GRM2	2912; NP 000830	XP_343471	AAO85115	XP_425148	CAG06804<br>CAG06963	FRUP00000141550<br>FRUP00000149314
GRM3	2913; NP 000831	XP_001069653	NP_862898	XP_416842	CAG13037	FRUP00000129944
GRM4	2914; NP 000832	NP_073157	XP_196179	XP_418031	CAG04030	FRUP00000150481
GRM5	2915; NP 000833	NP_058708	XP_149971	NP_989469	CAG00460	FRUP00000140545<br>FRUP00000140546
GRM6	2916; NP 000834	NP_075209	NP_775548	XP_428614	CAF89911	FRUP00000164837
GRM7	2917; NP 870990	NP_112302	NP_796302	XP_414442	CAF96886<br>CAG11283	FRUP00000132600<br>FRUP00000164838
GRM8	2918; NP 000836	NP_071538	NP_032200	XP_425426	CAG01066<br>CAG12082	FRUP00000147943<br>FRUP00000150688
**Subtotal**						
**14**	**16**	**16**	**16**	**14**	**19**	**23**

MRCA	Human gene ID; accession number	Rat	Mouse	Chicken	*T. nigroviridis*	*T . rubripes*
**Subclass F**						
FZD1/FZD2	8321; NP 003496<br>2535; NP 001457	NP 067089<br>NP 742032	NP 067432<br>NP 065256	XP 418648<br>NP 989553	CAG02812	FRUP00000148265
FZD3	7976; NP 059108	NP_703204	NP_067433	XP_420029	CAG00137<br>CAG11035	FRUP00000137946<br>FRUP00000150170
FZD4	8322; NP 036325	NP_072145	NP_032081	NP_989430	CAF96654	FRUP00000140126
FZD5	7855; NP 003459	NP_776210	NP_073558	XP_426568	CAF94116	FRUP00000135762
FZD6	8323; NP 003497	XP_343231	NP_032082	XP_418372	CAF98078	FRUP00000130766
FZD7	8324; NP 003498	XP_237191	NP_032083	NP_989552	CAF91294<br>CAG01343	FRUP00000143203
FZD8	8325; NP 114072	XP 344617	NP 032084	XP 418566	CAF97764<br>CAF98771	FRUP00000148131<br>FRUP00000159741<br>scaffold 122<br>scaffold 1549
FZD9	8326; NP 003499	NP_695217	XP_284144	XP_425392	CAF91428	FRUP00000142700
FZD10	11211; NP 009128	ENSRNOT00000029702	NP_780493	NP_989429	CAF94498	FRUP00000142418
SMO	6608; NP 005622	NP_036939	NP_795970	XP_414970	CAF97226	FRUP00000141346
**Subtotal**						
**10**	**11**	**11**	**11**	**11**	**13**	**14**

Table S2 C. List of nGPCR genes for which an ancestral form in the MRCA of tetrapods and teleosts can not be defined.

Tetrapod-specific genes

Class A

Subclass A1

Gene Name	Human Gene ID	Rat	Mouse	Chicken	*T. nigroviridis*	*T. rubripes*
OPN1MW	2652	NP_446000	NP_032132			

Subclass A2

TRAR3f/TAR3	134860	NP_783175<br>NP_783176<br>NP_001009975<br>NP_783181<br>NP_783180<br>NP_783178<br>NP_783177<br>NP_783192	NP_001010831<br>NP_001010827<br>NP_001010829<br>NP_001010835<br>NP_001010838<br>NP_001010839			

Subclass A4

GPR109B/HM74	8843					
GPR82	27197		NP_783600	NW_060226		
OXER1/TG1019	165140					

Subclass A5

GNRHR	2798	NP_112300	NP_034453	NP_989984		
GPR150/PGR11	285601	XP_574811	NP_780704			
GPR154/NPS receptor	387129	XP_235968	NP_783609	XP_426022		

Subclass A7

BDKRB2	624	NP_775123	NP_033877	XP_426461		
CCBP2	1238	NP_511176	NP_067622	XP_418499		
CCR8	1237	XP_236704	NP_031746	XP_418819		
CCRL2	9034	XP_236658	NP_059494			
CXCR6	10663		NP_109637	AAC23950		
FY	2532	NW_047399	NP_034175			
GPR120	338557	XP_215281	NP_861413	XP_426520		

Subclass A8

MAS1	4142	NP_036889	NP_032578	XP_427992<br>XP_427991<br>XP_426151<br>XP_423677		
MAS1L	116511					
MRGPRD	116512	NP_001001506	NP_987075			
MRGPRE	116534	NP_001002288	NP_780743			
MRGPRF/MRGF	116535	NP_714944	NP_663354			
MRGPRG/MRGG	386746	NP_982296	NP_987077			
MRGPRH		NP_001002281	NP_109651			

MRGX1e/MRGX1	259249					
MRGX2e/MRGX2	117194	NP_665730<br>NP_001002282<br>NP_001002286<br>NP_750845	NP_694735<br>NP_694741<br>NP_694707<br>NP_705744<br>NP_997417<br>NP_991390<br>NP_997418<br>NP_997419<br>NP_997423<br>AX299180<br>AX299182<br>AX299184<br>AX299186<br>AX299188<br>AX299190<br>AX299192<br>AX299194<br>AX299200			
MRGX3e/MRGX3	117195					
MRGX4e/MRGX4	117196					
rat_MrgB3		NP_001002280<br>NP_001002285<br>AAQ08313<br>NP_001002287<br>NP_001002284<br>AAQ08316<br>GeneID_404639<br>NP_001002283<br>GeneID_404654<br>GeneID_404657	NP_780740<br>NP_997420<br>NP_991364<br>NP_997421<br>NP_997422<br>NP_991379<br>AX299214			

Class B

GIPR	2696	NP_036846	NP_001074284			
SCTR	6344	NP_112377	NP_001012322	XP_422120		

Teleost-specific genes

Class A

Subclass A1

OPN1MW-like					CAG06878.1<br>CAG08706.1<br>CAG10163.1	FRUP00000141947<br>FRUP00000144085<br>FRUP00000151874

Subclass A2

ADRA2D-like					CAF91577.1	CAC87886.1<br>CAC87887.1

Subclass A5

CCKR-like					CAG00996.1<br>CAG05857.1	FRUP00000135669<br>FRUP00000162216<br>FRUP00000162217

Table S2 D. Correlation coefficients of nGPCR inventories between pairs of species.

	**Human**	**Rat**	**Mouse**	**Chicken**	***T. nigroviridis***	***T. rubripes***
**Human**	1	0.8754	0.9136	0.5012	0.1239	0.0696
**Rat**	0.8754	1	0.9561	0.4943	0.2071	0.1150
**Mouse**	0.9136	0.9561	1	0.5003	0.2159	0.1165
**Chicken**	0.5012	0.4943	0.5003	1	0.1659	0.1871
T. nigroviridis	0.1239	0.2071	0.2159	0.1659	1	0.7903
T. rubripes	0.06960	0.1150	0.1165	0.1871	0.7903	1

Table S2 E. Number of singleton and duplicated nGPCR genes in model vertebrates, and the number of nGPCR families with gene duplicates in each species.

	Human	Rat	Mouse	Chicken	*T. nigroviridis*	*T. rubripes*
Singletons (% of total)	225 (67.8)	216 (67.7)	218 (65.7)	185 (62.5)	149 (35.1)	148 (34.4)
Duplicates (% of total)	107 (32.2)	103 (32.3)	114 (34.3)	111 (37.5)	276 (64.9)	282 (65.6)
Total	332	319	332	296	425	430
No. of nGPCR families with duplicates (% of total families analyzed)	44 (16.4)	42 (16.3)	44 (16.8)	50 (21.3)	111 (42.7)	106 (41.7)
No. of nGPCR families identified	269	258	262	235	260	254
