# Supplementary material for: GPCR Genes Are Preferentially Retained after Whole Genome Duplication
Source: PLoS One. 2008 Apr 2;3(4):e1903. doi: 10.1371/journal.pone.0001903 (PMC2270905; doi:10.1371/journal.pone.0001903)
Supplement: Table S3 — A. Tandem duplication-derived nGPCRs of T. nigroviridis. Tandem duplication-derived genes are defined as paralogous genes found on neighboring loci on the same chromosome. B. Summary of nGPCRs derived from tandem duplication in tetrapods. Tandem duplication-derived genes are defined as paralogous genes found on neighboring loci on the same chromosome. C. List of tandem duplication-derived nGPCRs of tetrapods. The putative origins of these tandem duplication-derived paralogs are indicated on the left column. D. WGD-derived nGPCRs of T. nigroviridis. WGD-derived genes are defined as co-orthologous genes found on WGD-derived syntenic regions on different chromosomes of pufferfish. E. Schematic representation of the WGD-derived GPR61 duplicates on T. nigroviridis chromosomes 9 and 11. The position of WGD-derived GPR61 and neighboring Alivin-2 genes on T. nigroviridis chromosomes as well as their orthologs on human chromosome 1 are indicated by italicized letters. (0.03 MB PDF) [file pone.0001903.s005.pdf]

Table S3 A. Tandem duplication-derived nGPCRs of *T. nigroviridis*

	Class	MRCA	*T. nigroviridis* chromosome	*T. nigroviridis* copy 1	*T. nigroviridis* copy 2	Human chromosome
1	A1	GPR62	chr11	CAG06806.1	CAG06960.1	chr3
2	A1	GPR101	chr1	CAG09823.1	SCAF13862	chrX
3	A1	OPN5	chr9	CAG03922.1	CAG11258.1	chr6
4	A1	OPN5	chr14	CAG13006.1	CAG00077.1	chr6
5	A1	RHO	chr9	CAG11315.1	CAG11334.1	chr3
6	A2	ADORA1/ADORA3	chr11	CAF96797.1	CAG11815.1	chr1
7	A2	HTR2B	chr1	CAC85912.1	CAC86247.1	chr2
8	A3	EDG5	chr3	SCAF15050	chr3_11567k-11569k	chr19
9	A4	GPR4/GPR132	chr16	CAF97465.1	CAG12733.1	chr19
10	A4	EBI2	chr2	CAG07299.1	chr2:7825k-7877k	chr13
11	A5	AVPR2	chr9	CAG03798.1	CAG07446.1	chrX
12	A5	GPR103	chr2_random	CAG04805.1	SCAF14750	chr4
13	A5	OXTR	chr11	CAG06809.1	CAG06958.1	chr3
14	A5	TACR2/TACR3	chr18	CAG01274.1	CAG11520.1	chr4
15	A7	RLN3R1/GPCR135	chr5	CAF99875.1	CAF99890.1	chr5
16	B	BAI3	chr17	CAG09441.1	GSTENT00030703001	chr6
17	C	GRM2	chr11	CAG06804.1	CAG06963.1	chr3

Table S3 B. Summary of nGPCRs derived from tandem duplication in tetrapods.

	Subclass	MRCA	Human	Rat	Mouse	Chicken
1	**A1**	**OPN5**				2
2	**A2**	**ADORA1**				2
3		**TRAR3f**		8	6	
4		**TRAR1**	3	4	5	
5		**TRAR4**	2	4	5	
6	**A3**	**PTGER2**	2	2	2	
7	**A4**	**P2RY12**	3	2	3	3
8		**GPR174**	2	2	2	3
9		**CYSLTR1**				2
10		**GPR23**	4	3	2	
11		**GPR43**	2	2	2	
12		**GPR109A**	2	2	2	
13		**GPR41**	2			
14	**A5**	**NPY5R**	2	2	2	2
15		**PKR1**	2	2	2	2
16	**A7**	**CCR4**	5	6	4	3
17		**UTS2R**				2
18		**LTB4R**	2	2	2	
19		**FPR1**	4	6	7	
20	**A8**	**MAS1**	7	17	28	3
21	**B**	**GPR116**	4	3	4	3
22		**ADCYAP1R1**				2
23		**EMR1**	4		2	
24	**C**	**GPRC5B**	4	3	4	
	**Total number of TD-derived genes**		56	70	84	29
	**Total number of orthologous nGPCR groups with TD**		18	17	18	12

TD, Tandem duplication

Table S3 C. List of tandem duplication-derived nGPCRs of tetrapods.

Origin of duplication		Subclass	MRCA	Human Gene Name	Human	Rat	Mouse	Chick
Present in all four tetrapods	1	A4	**P2RY12**	**GPR171, GPR87, P2RY12**	NP_037440, NP_076404, NP_795345	XP_227177, NP_073637	NP_775574, NP_115775, NP_081847	XP_422842, XP_422840, XP_422839
	2	A4	**GPR174**	**GPR174, P2RY10**	NP_115942, NP_938147	XP_228495, XP_228500	XP_142040, NP_766023	NP_001008464, XP_420149, XP_420148
	3	A5	**NPY5R**	**NPY1R**	NP_000900, NP_006165	NP_001013050, NP_037001	NP_035064, NP_057917	XP_426285, NP_001026301
	4	A5	**PKR1**	**GPR73L1, PKR1**	NP_658986, NP_620414	NP_620434, NP_620433	NP_659193, NP_067356	XP_419334, XP_419333
	5	A7	**CCR4**	**CCR1, CCR2, CCR3, CCR4, CCR5**	NP_001286, NP_000639, NP_847899, NP_005499, NP_000570	NP_065417, XP_236742, NP_068638, NP_446410, NP_598216, NP_446412	NP_034042, NP_034045, NP_034044, NP_034046	XP_426017, NP_001039300, NP_001039299
	6	A8	**MAS1**	**MAS1, MRGPRD, MRGPRDE, MRGPRF, MRGX1e, MRGX2e, MRGX3e, MRGX4e**	NP_944605, NP_659452, NP_671732, NP_473371, NP_473372, NP_473373, NP_000530	NP_036889, NP_001001506, NP_001002288, NP_714944, NP_982296, NP_001002282, NP_001002286, NP_665730, NP_750845, AAQ08313, AAQ08316, NP_001002280, NP_001002283, NP_001002284, NP_001002285,NP_001002287, NP_001002281	NP_987075, NP_780743, NP_663354, NP_987077, NP_997423, NP_694707, NP_694735, NP_694741, NP_705744, NP_991390, NP_997417, NP_997418, NP_997419, NP_780740, NP_991364, NP_991379, NP_997420, NP_997421, NP_997422, NP_109651, AX299180, AX299182, AX299184, AX299186, AX299188, AX299190, AX299192, AX299194	XP_427992, XP_427991, XP_423677
	7	B	**GPR116**	**GPR110, GPR111, GPR115, GPR116**	NP_079324, NP_722581, NP_722580, NP_056049	XP_217359, XP_236958, NP_620810	NP_598537, XP_487485, XP_128679, XP_283438	XP_420068, XP_420066
	8	A4	**GPR41**	**GPR41, GPR42a**	NP_005295, NP_005296			
	9	A2	**TRAR3f**			NP_001009975, NP_783175, NP_783176, NP_783177, NP_783178, NP_783180, NP_783181, NP_783192	NP_001010831, NP_001010827, NP_001010829, NP_001010835, NP_001010838,NP_001010839	

Present only in mammals	10	A2	**TRAR1**	**GPR57g, PNRe, TRAR1**	NC_000006, NP_003958, NP_778227	NP_001009532, NP_001009650, NP_599155, NP_783173	NP_001008429, NP_001009574, NP_038736, NP_444435	
	11	A2	**TRAR4**	**TRAR4, TRAR5**	NP_778237, NP_444508	NP_783174, NP_783189, NP_783190, NP_783191	NP_001010828, NP_001008499, NP_001010830, NP_001010840, NP_001010837	
	12	A3	**PTGER2**	**PTGDR, PTGER2**	NP_000944, NP_000947	NP_071577, NP_112350	NP_032988, NP_032990	
	13	A4	**GPR23**	**GPR23, GPR35, GPR55, GPR92**	NP_005292, NP_005674	NP_001032436, AAD22411, XP_575667	NP_071715, XP_136804	
	14	A4	**GPR43**	**GPR40, GPR43**	NP_005294, NP_005297	NP_695216, NP_001005877	NP_918946, NP_666299	
	15	A4	**GPR109A**	**GPR109A, GPR31, GPR81**	NP_808219, NP_115943	NP_852141, NP_780729	NP_109626, NP_780729	
	16	A7	**LTB4R**	**LTB4R, LTB4R2**	NP_858043, NP_062813	NP_067688, NP_446092	NP_032545, NP_065236	
	17	A7	**FPR1**	**FPR1, FPRL1, FPRL2, GPR152, GPR32**	NP_002020, NP_001453, NP_002021, NP_001497	XP_218012, XP_218016, XP_218022, XP_001057995, XP_001073753, XP_001068404	NP_038549, NP_032068, XP_622087, EDL38017, AAC34587, AAN63620, AAN63621	
	18	C	**GPRC5B**	**GPRC5A, GPRC5B, GPRC5D, C5R1, GPR77**	NP_003970, NP_061124, NP_001727, NP_060955	NP_001073359, XP_575701, NP_001003710	NP_852109, NP_444348, NP_031603, NP_795886	
	19	B	**EMR1**	**EMR1, EMR2, EMR3, EMR4b**	NP_001965, NP_690883, NP_693634, XP_377506		NP_034260, NP_631877	
Present only in chicken	20	A1	**OPN5**	**OPN5**				XP_426228, XP_420056
	21	A2	**ADORA1**	**ADORA1**				NP_989647, NP_989482
	22	A4	**CYSLTR1**	**CYSLTR1**				XP_426249, XP_425629
	23	A7	**UTS2R**	**UTS2R**				XP_425371, XP_425370
	24	B	**ADCYAP1R1**	**ADCYAP1R1**				XP_425958, NP_001092076

Table S3 D. WGD-derived nGPCRs of *T. nigroviridis*

Class	MRCA	*T. nigroviridis* chromosomes	*T. nigroviridis* copy 1	*T. nigroviridis* copy 2	Human chromosome
A1	GPR26	chr 2;17	CAG05074.1	GSTENT00035915001	chr10
A1	GPR61	chr 9;11	CAG06826.1	CAG07504.1	chr1
A1	OPN3-like	chr 1;7	CAG08854.1	CAG09827.1	chrX
A1	OPN4	chr 4;12	CAF99228.1	CAG08413.1	chr10
A1	RE2/GPR161	chr 2;17	CAG05829.1	CAG09451.1	chr1
A1	RGR	chr 2;17	CAF98663.1	CAG13313.1	chr10
A1	RHO	chr 9;11	CAG06941.1	CAG11334.1	chr3
A2	ADORA1/ADORA3	chr 9;11	CAG07486.1	CAG11815.1	chr1
A2	ADRA1B/GPR88	chr 1;7	CAF92745.1	CAF97256.1	chr5
A2	ADRA2A	chr 17;18	CAG01293.1	CAG05052.1	chr10
A2	CHRM2	chr 13;19	CAG02408.1	CAG03403.1	chr7
A2	CHRM4	chr 3;13	CAG05625.1	CAG06610.1	chr11
A2	DRD1	chr 1;7	CAF95884.1	CAG11724.1	chr5
A2	GPR21	chr 4;12	CAF93159.1	CAG09652.1	chr9
A2	HRH2	chr 1;7	CAG01551.1	CAG04750.1	chr5
A2	HRH4/HRH3	chr 1;15	CAG07613.1	CAG10949.1	chr18
A3	CNR1	chr 10;14	CAG09211.1	CAG10478.1	chr6
A3	PTGER4	chr 4;12	CAF97110.1	CAG09586.1	chr5
A4	GPR20	chr 8;21	CAF99361.1	CAG11945.1	chr8

Class	**MRCA**	***T. nigroviridis* chromosomes**	**T. nigroviridis copy 1**	***T. nigroviridis copy 2***	**Human chromosome**
A4	P2RY2	chr 7;10	CAG00917.1	CAF97358.1	chr11
A4	P2Y5/P2RY5	chr 7;16	CAG02919.1	CAG12785	chr13
A5	EDNRB	chr 2;15	CAF97649.1	CAG05905.1	chr13
A5	GPR74/NPFF receptor 2	chr 4;12	CAF97104.1	CAG07773.1	chr4
A5	GRPR/BRS3	chr 2;5	CAF99824.1	CAG05835.1	chrX
A7	IL8RB	chr2;3	CAF98209.1	chr2:16451193_2269	chr2
A7	CXCR4	chr 2;15	CAF97662.1	CAG01848.1	chr2
A7	RLN3R1	chr 1;15	CAG01356.1	CAG07748.1	chr19
A7	RLN3R2	chr 5;13	CAF89883.1	CAF99875.1	chr15
B	CLR	chr 2;3	CAF98969.1	CAG01331.1	chr2
B	GPR64	chr 6;7	CAG00549.1	CAG05443.1	chrX
B	GPR112	chr 1;7	CAG09829.1	CAG11729.1	chrX
B	LPHN1	chr 3;18	CAG06092	chr3_11032320_65518	chr19
B	LPHN2	chr 1;15	CAF95119	CAF98480	chr1
B	VIPR1	chr 6;15	CAF97690.1	CAG04243.1	chr3
C	GPRC5B/GPRC5A/GPRC 5D	chr 3;18	CAG06055.1	CAG11409.1	chr16
C	GPRC5C	chr 2;3	CAG00236.1	CAG03050.1	chr17
C	GRM1	chr 10;14	CAG03586.1	CAG13194.1	chr6
C	GRM7	chr 9;11	CAF96886.1	CAG11283.1	chr3
F	FZD7	chr 2;3	CAF91294.1	CAG01343.1	chr2

Table S3 E. Schematic representation of the WGD-derived *GPR61* duplicates on *T. nigroviridis* chromosomes 9 and 11.

First syntenic region on *T. nigroviridis* chromosome 11	Gene	Human Chromosome 1	Second syntenic region on *T. nigroviridis* chromosome 9
**11** : 5919052-5922303	AHCYL1	**1** : 110239350-110277038	-
**11** : 5924983-5929169	NP_149079.2	**1** : 110289296-110309287	-
**11** : 5931462-5935646	SLC6A17	**1** : 110446636-110453108	-
**11** : 5931462-5935646	SLC6A17	**1** : 110421594-110429630	-
**11** : 5940778-5943221	RBM15	**1** : 110593170-110601337	-
-	EPS8L3	**1** : 110004744-110018606	**9** : 2866270-2869733
-	GSTM3	**1** : 109988596-109995702	**9** : 2869973-2872055
-	GSTM4	**1** : 109910745-109920166	**9** : 2869973-2872055
-	GSTM2	**1** : 109922733-109929947	**9** : 2869973-2872055
-	GSTM5	**1** : 109942484-109948409	**9** : 2869973-2872055
-	GSTM1	**1** : 109966907-109972930	**9** : 2869973-2872055
-	AMPD2	**1** : 109874501-109886798	**9** : 2873619-2877462
-	GNAT2	**1** : 109857817-109867747	**9** : 2901821-2907129
11 : 5947339-5948830	***Alivin-2***	***1 : 109761489-109764346***	***9 : 2934618-2936132***
-	GNAI3	**1** : 109803343-109849017	**9** : 2907966-2918306
11 : 5954943-5956340	***GPR61***	***1 : 109794551-109800497***	***9 : 2921704-2922744***
**11** : 5966635-5971303	AMPD2	**1** : 109874501-109886798	-
-	ATXN7L2	**1** : 109738603-109747396	**9** : 2939359-2942203
-	Sypl2	**1** : 109721142-109734212	**9** : 2942523-2945526
-	SORT1	**1** : 109568465-109652615	**9** : 2947941-2955504
**11** : 5973422-5977119	EPS8L3	**1** : 110004744-110018606	-
**11** : 5978240-5980600	IRF6	**1** : 206349657-206367874	-
