# Supplementary material for: GPCR Genes Are Preferentially Retained after Whole Genome Duplication
Source: PLoS One. 2008 Apr 2;3(4):e1903. doi: 10.1371/journal.pone.0001903 (PMC2270905; doi:10.1371/journal.pone.0001903)
Supplement: Table S4 — A. List of the open reading frame (ORF) lengths of representative human receptors in each of the 269 nGPCR families inferred in the MRCA of tetrapods and teleosts. The lengths of human nGPCR ORFs were obtained from GenBank, and the majority of these ORFs have been defined experimentally. B. List of MWs of cognate ligands for each of the 190 families of nGPCRs with a known ligand(s). WGD, whole genome duplication; TD, tandem duplication; ND, not detected in T. nigroviridis; UD, undetermined; S, singleton. (0.03 MB PDF) [file pone.0001903.s006.pdf]

Table S4 A. List of the open reading frame (ORF) lengths of representative human receptors in each of the 269 nGPCR families.

Receptor class	Representative human GPCR	Human gene ID	ORF length (amino acids)	Origin of pufferfish homologs
A1	GPR26	2849	337	WGD
A1	GPR61	83873	451	WGD
A1	OPN3	23596	403	WGD
A1	OPN4	94233	478	WGD
A1	RE2	23432	529	WGD
A1	RGR	5995	295	WGD
A1	OPN5	221391	354	TD
A1	GPR101	83550	508	TD
A1	GPR78	27201	363	UD
A1	GPR62	118442	368	TD
A1	MTNR1A	4543	350	UD
A1	RHO	6010	348	WGD/TD
A1	GPR135	64582	494	S
A1	GPR50	9248	613	S
A1	GPR84	53831	396	S
A1	MTNR1B	4544	362	S
A1	OPN1LW	5956	364	S
A1	OPN1SW	611	348	S
A1	RRH	10692	337	S
A2	ADRA1B	147	520	WGD
A2	ADRA2A	150	450	WGD
A2	CHRM2	1129	466	WGD
A2	CHRM4	1132	478	WGD
A2	DRD1	1812	446	WGD
A2	GPR21	2844	349	WGD
A2	HRH2	3274	359	WGD
A2	HRH4	59340	445	WGD
A2	HTR2B	3357	481	TD
A2	ADORA2A	135	412	UD
A2	ADORA2B	136	332	UD
A2	ADRA2C	152	461	UD
A2	ADRB1	153	477	UD
A2	CHRM3	1131	590	UD
A2	DRD2	1813	443	UD
A2	DRD4	1815	467	UD
A2	DRD5	1816	477	UD
A2	GPR22	2845	433	UD
A2	HTR1A	3350	421	UD
A2	HTR4	3360	338	UD
A2	HTR7	3363	479	UD
A2	TRAR1	134864	339	UD
A2	ADORA1	134	326	WGD/TD
A2	ADRA1A	148	499	S
A2	ADRA1D	146	572	S
A2	ADRA2B	151	450	S
A2	ADRB2	154	413	S
A2	CHRM1	1128	460	S
A2	CHRM5	1133	532	S
A2	DRD3	1814	400	S
A2	GPR119	139760	335	S
A2	GPR52	9293	361	S
A2	GPR58	9287	306	S
A2	GPR75	10936	540	S
A2	HRH1	3269	487	S
A2	HTR1B	3351	390	S
A2	HTR1D	3352	377	S
A2	HTR1F	3355	365	S
A2	HTR2C	3358	471	S
A2	HTR5B	3361	357	S

A2	HTR6	3362	440	S
A2	TRAR4	319100	345	ND in *T. nigroviridis*
A3	CNR1	1268	472	WGD
A3	EDG3	1903	378	S
A3	PTGER4	5734	488	WGD
A3	EDG5	9294	370	TD
A3	EDG2	1902	364	UD
A3	GPR12	2835	334	UD
A3	CNR2	1269	360	S
A3	EDG1	1901	381	S
A3	EDG7	23566	353	S
A3	EDG8	53637	398	S
A3	GPR6	2830	362	S
A3	MC1R	4157	317	S
A3	MC2R	4158	297	S
A3	MC4R	4160	332	S
A3	MC5R	4161	325	S
A3	PTGER2	5732	359	S
A3	PTGER3	5733	402	S
A3	PTGFR	5737	402	S
A3	PTGIR	5739	386	S
A3	TBXA2R	6915	343	S
A3	EDG4	9170	351	ND in *T. nigroviridis*
A4	EBI2	1880	361	WGD
A4	GPR20	2843	358	WGD
A4	GPR23	2846	370	S
A4	P2RY2	5029	377	WGD
A4	P2Y5	10161	344	WGD
A4	GPR4	2828	380	TD
A4	F2RL1	2150	397	UD
A4	F2RL2	2151	374	UD
A4	GPR109A	338442	363	UD
A4	GPR17	2840	367	UD
A4	GPR43	2867	300	UD
A4	P2RY1	5028	373	UD
A4	ADMR	11318	404	S
A4	F2R	2149	425	S
A4	F2RL3	9002	385	S
A4	GPR105	9934	338	S
A4	GPR141	353345	305	S
A4	GPR174	84636	333	S
A4	GPR18	2841	331	S
A4	GPR34	2857	305	S
A4	GPR41	2865	346	S
A4	GPR65	8477	337	S
A4	GPR68	8111	365	S
A4	GPR80	27199	337	S
A4	GPR86	53829	333	S
A4	GPR91	56670	330	S
A4	P2RY11	5032	374	S
A4	P2RY12	64805	319	S
A4	P2RY4	5030	365	S
A4	P2RY6	5031	328	S
A4	PTAFR	5724	342	S
A4	RDC1	57007	362	S
A4	CYSLTR1	10800	337	ND in *T. nigroviridis*
A4	P2RY8	286530	359	ND in *T. nigroviridis*
A5	EDNRB	1910	442	WGD
A5	GNRHR2-(partial)	114814	292	S
A5	GPR74	10886	522	WGD
A5	GRPR	2925	399	WGD
A5	AVPR2	554	371	TD
A5	GPR103	84109	431	TD
A5	OXTR	5021	389	TD
A5	TACR3	6870	398	TD
A5	AVPR1A	552	418	UD
A5	EDNRA	1909	427	UD

A5	GPR10	2834	370	UD
A5	GPR173	54328	373	UD
A5	LHCGR	3973	699	UD
A5	NMBR	4829	390	UD
A5	NPY2R	4887	381	UD
A5	NPY5R	4889	384	UD
A5	TACR1	6869	407	UD
A5	AVPR1B	553	424	S
A5	CCKAR	886	428	S
A5	FSHR	2492	695	S
A5	GPR	11245	515	S
A5	GPR19	2842	415	S
A5	GPR37	2861	613	S
A5	GPR37L	9283	481	S
A5	GPR45	11250	376	S
A5	GPR63	81491	419	S
A5	GPR73	10887	393	S
A5	GPR83	10888	423	S
A5	GPR85	54329	370	S
A5	HCRTR2	3062	425	S
A5	LGR4	55366	951	S
A5	LGR5	8549	907	S
A5	LGR6	59352	828	S
A5	LGR7	59350	757	S
A5	LGR8	122042	754	S
A5	NPFFR1	64106	430	S
A5	PPYR1	5540	375	S
A5	TSHR	7253	764	S
A6	NMUR1	10316	426	S
A6	TRHR	7201	398	UD
A6	GHSR	2693	366	S
A6	GPR38	2862	412	S
A6	GPR39	2863	453	S
A6	NMUR2	56923	415	S
A6	NTSR1	4923	418	S
A7	CXCR4	7852	352	WGD
A7	GPR151	134391	419	WGD
A7	RLN3R1	51289	469	WGD
A7	AGTRL1	187	380	UD
A7	BLR1	643	372	UD
A7	CCR9	10803	369	UD
A7	CMKLR1	1240	371	UD
A7	GALR1	2587	349	UD
A7	GPR14	2837	389	UD
A7	LTB4R	1241	352	UD
A7	OPRK1	4986	380	UD
A7	SSTR2	6752	369	UD
A7	SSTR3	6753	418	UD
A7	AGTR1	185	359	S
A7	AGTR2	186	363	S
A7	BDKRB2	624	391	S
A7	C5R1	728	482	S
A7	CCR5	1234	384	S
A7	CCR6	1235	374	S
A7	CCR7	1236	374	S
A7	CCR10	2826	362	S
A7	CCR11	51554	350	S
A7	CX3CR1	1524	355	S
A7	CXCR3	2833	368	S
A7	FPR1	2357	350	S
A7	GALR2	8811	387	S
A7	GPR139	124274	661	S
A7	GPR142	350383	469	S
A7	GPR145	2847	422	S
A7	GPR146	115330	333	S
A7	GPR24	84539	340	S
A7	GPR25	2848	360	S

A7	GPR30	2852	375	S
A7	GPR44	11251	395	S
A7	GPR54	84634	398	S
A7	GPR8	2832	328	S
A7	IL8RA	3577	350	S
A7	IL8RB	3579	360	S
A7	OPRD1	4985	372	S
A7	OPRL1	4987	370	S
A7	OPRM1	4988	400	S
A7	SSTR5	6755	364	S
A7	GPR1	2825	355	ND in *T. nigroviridis*
A7	SSTR1	6751	391	ND in *T. nigroviridis*
A7	XCR1	2829	333	UD
A7	RLN3R2	339403	374	WGD/TD
B	CLR	10203	461	WGD
B	GPR112	139378	2799	WGD
B	GPR64	10149	1014	WGD
B	LPHN2	23266	1403	WGD
B	VIPR1	7433	457	WGD
B	BAI3	577	1522	TD
B	ADCYAP1R1	117	525	UD
B	BAI1	575	1584	UD
B	CR	799	474	UD
B	GCGR	2642	477	UD
B	LPHN1	22859	1474	UD
B	LPHN3	23284	1240	UD
B	PTHR1	5745	593	UD
B	VIPR2	7434	438	UD
B	BAI2	576	1572	S
B	CD97	976	835	S
B	CELSR1	9620	3014	S
B	CELSR2	1952	2923	S
B	CELSR3	1951	3312	S
B	CRHR1	1394	415	S
B	ELTD1	64123	606	S
B	EMR1	2015	886	S
B	GHRHR	2692	423	S
B	GLP1R	2740	463	S
B	GLP2R	9340	553	S
B	GPR114	221188	528	S
B	GPR116	221395	915	S
B	GPR123	84435	1280	S
B	GPR124	25960	1331	S
B	GPR125	166647	1124	S
B	GPR126	57211	404	S
B	GPR144	347088	332	S
B	GPR56	9289	693	S
B	PTHR2	5746	550	S
B	VLGR1	84059	6307	S
B	CRHR2	1395	411	ND in *T. nigroviridis*
B	GPR133	283383	633	ND in *T. nigroviridis*
B	GPR97	222487	549	ND in *T. nigroviridis*
C	GPRC5B	51704	357	WGD
C	GPRC5C	55890	453	WGD
C	GRM1	2911	1194	WGD
C	GRM7	2917	915	WGD
C	GRM2	2912	872	TD
C	GRM8	2918	908	UD
C	CASR	846	1078	S
C	GABBR1	2550	961	S
C	GPR51	9568	941	S
C	GRM3	2913	877	S
C	GRM4	2914	912	S
C	GRM5	2915	1180	S
C	GRM6	2916	877	S
C	GPRC6A	222545	926	ND in *T. nigroviridis*
F	FZD7	8324	574	WGD

F	FZD3	7976	666	UD
F	FZD8	8325	694	UD
F	FZD10	11211	581	S
F	FZD4	8322	537	S
F	FZD5	7855	585	S
F	FZD6	8323	706	S
F	FZD9	8326	591	S
F	SMO	6608	787	S
F	FZD1	8321	648	S

Table S4 B. List of MWs of cognate ligands for each of the 190 families of nGPCRs with a known ligand(s).

Receptor class	Representative human GPCR	Human gene ID	Origin of pufferfish homologs	Chemical properties of ligands	Representative ligand	Ligand size Log_{10}(MW)
A1	MTNR1A	4543	UD	amine	Melatonin	2.37
A1	MTNR1B	4544	S	amine	Melatonin	2.37
A1	OPN1LW	5956	S	photon	photon	0.00
A1	OPN1SW	611	S	photon	photon	0.00
A1	OPN3	23596	WGD	photon	photon	0.00
A1	OPN4	94233	WGD	photon	photon	0.00
A1	OPN5	221391	TD	photon	photon	0.00
A1	RGR	5995	WGD	photon	photon	0.00
A1	RHO	6010	WGD	photon	photon	0.00
A1	RRH	10692	S	photon	photon	0.00
A2	ADORA1	134	WGD	nucleotide	Adenosine	2.43
A2	ADORA2A	135	UD	nucleotide	Adenosine	2.43
A2	ADORA2B	136	UD	nucleotide	Adenosine	2.43
A2	ADRA1A	148	S	amine	Noradrenaline	2.23
A2	ADRA1B	147	WGD	amine	Adrenaline	2.26
A2	ADRA1D	146	S	amine	Adrenaline	2.26
A2	ADRA2A	150	WGD	amine	Adrenaline	2.26
A2	ADRA2B	151	S	amine	Adrenaline	2.26
A2	ADRA2C	152	UD	amine	Adrenaline	2.26
A2	ADRB1	153	UD	amine	Noradrenaline	2.23
A2	ADRB2	154	S	amine	Adrenaline	2.26
A2	CHRM1	1128	S	amine	Acetylcholine	2.16
A2	CHRM2	1129	WGD	amine	Acetylcholine	2.16
A2	CHRM3	1131	UD	amine	Acetylcholine	2.16
A2	CHRM4	1132	WGD	amine	Acetylcholine	2.16
A2	CHRM5	1133	S	amine	Acetylcholine	2.16
A2	DRD1	1812	WGD	amine	Dopamine	2.19
A2	DRD2	1813	UD	amine	Dopamine	2.19
A2	DRD3	1814	S	amine	Dopamine	2.19

A2	DRD4	1815	UD	amine	Dopamine	2.19
A2	DRD5	1816	UD	amine	Dopamine	2.19
A2	HRH1	3269	S	amine	Histamine	2.05
A2	HRH2	3274	WGD	amine	Histamine	2.05
A2	HRH4	59340	WGD	amine	Histamine	2.05
A2	HTR1A	3350	UD	amine	5-Hydroxytryptamine	2.25
A2	HTR1B	3351	S	amine	5-Hydroxytryptamine	2.25
A2	HTR1D	3352	S	amine	5-Hydroxytryptamine	2.25
A2	HTR1F	3355	S	amine	5-Hydroxytryptamine	2.25
A2	HTR2B	3357	TD	amine	5-Hydroxytryptamine	2.25
A2	HTR2C	3358	S	amine	5-Hydroxytryptamine	2.25
A2	HTR5B	3361	S	amine	5-Hydroxytryptamine	2.25
A2	HTR6	3362	S	amine	5-Hydroxytryptamine	2.25
A2	HTR7	3363	UD	amine	5-Hydroxytryptamine	2.25
A2	TRAR1	134864	UD	amine	Phenylethylamine, tyramine	2.08
A3	CNR1	1268	WGD	lipophylic molecule	Cannabinoid	2.50
A3	CNR2	1269	S	lipophylic molecule	Cannabinoid	2.50
A3	EDG1	1901	S	lipophylic molecule	Sphingosine 1-phosphate	2.58
A3	EDG2	1902	UD	lipophylic molecule	Lysophosphatidic acid	2.64
A3	EDG3	1903	S	lipophylic molecule	Sphingosine 1-phosphate	2.58
A3	EDG4	9170	ND	lipophylic molecule	Lysophosphatidic acid	2.64
A3	EDG5	9294	TD	lipophylic molecule	Sphingosine 1-phosphate	2.58
A3	EDG7	23566	S	lipophylic molecule	Lysophosphatidic acid	2.64
A3	EDG8	53637	S	lipophylic molecule	Sphingosine 1-phosphate	2.58
A3	GPR12	2835	UD	lipophylic molecule	Sphingosine 1-phosphate	2.58
A3	GPR6	2830	S	lipophylic molecule	Sphingosine 1-phosphate	2.58
A3	MC1R	4157	S	peptide	Melanocyte stimulating hormone	3.42
A3	MC2R	4158	S	peptide	Adrenocorticotropic hormone	3.42
A3	MC4R	4160	S	peptide	Melanocyte stimulating hormone	3.42
A3	MC5R	4161	S	peptide	Melanocyte stimulating hormone	3.42
A3	PTGER2	5732	S	lipophylic molecule	Prostaglandin D2	2.55
A3	PTGER3	5733	S	lipophylic molecule	Prostaglandin E2	2.55
A3	PTGER4	5734	WGD	lipophylic molecule	Prostaglandin E2	2.55
A3	PTGFR	5737	S	lipophylic molecule	Prostaglandin E2	2.55

A3	PTGIR	5739	S	lipophylic molecule	Prostacyclin	2.55
A3	TBXA2R	6915	S	lipophylic molecule	Thromboxane A2	2.55
A4	CYSLTR1	10800	ND	lipophylic molecule	Leukotriene D4	2.70
A4	F2R	2149	S	peptide	Thrombin	3.63
A4	F2RL1	2150	UD	peptide	Serine proteases	3.63
A4	F2RL2	2151	UD	peptide	Thrombin	3.63
A4	F2RL3	9002	S	peptide	Serine proteases	3.63
A4	GPR105	9934	S	nucleotide	UDP-Glucose	2.75
A4	GPR109A	338442	UD	amine	Nicotinic acid (high affinity)	2.09
A4	GPR23	2846	S	lipophylic molecule	Lysophosphatidic acid	2.64
A4	GPR41	2865	S	lipophylic molecule	Carboxylic acids	2.16
A4	GPR43	2867	UD	lipophylic molecule	Long chain carboxylic acids	2.58
A4	GPR80	27199	S	lipophylic molecule	Ketoglutarate	2.16
A4	GPR86	53829	S	nucleotide	ADP	2.63
A4	GPR91	56670	S	lipophylic molecule	Succinate	2.07
A4	P2RY1	5028	UD	nucleotide	ADP	2.63
A4	P2RY11	5032	S	nucleotide	ATP	2.71
A4	P2RY2	5029	WGD	nucleotide	UTP, ATP	2.71
A4	P2RY4	5030	S	nucleotide	UTP	2.68
A4	P2RY6	5031	S	nucleotide	UDP	2.61
A4	PTAFR	5724	S	lipophylic molecule	Platelet-activating factor	2.72
A5	AVPR1A	552	UD	peptide	Vasopressin	3.04
A5	AVPR1B	553	S	peptide	Vasopressin	3.04
A5	AVPR2	554	TD	peptide	Vasopressin	3.04
A5	CCKAR	886	S	peptide	Cholecystokinin	3.19
A5	EDNRA	1909	UD	peptide	Endothelin 1, endothelin 2	3.63
A5	EDNRB	1910	WGD	peptide	Endothelins 1, 2 and 3	3.63
A5	FSHR	2492	S	peptide	Follicle-stimulating hormone	4.48
A5	GNRHR2	114814	S	peptide	Gonadotrophin-releasing hormone	3.04
A5	GPR10	2834	UD	peptide	Prolactin-releasing peptide	3.36
A5	GPR103	84109	TD	peptide	RF-Amide P518 gene product	3.40
A5	GPR73	10887	S	peptide	Prokineticins 1 and 2	3.56
A5	GPR74	10886	WGD	peptide	Neuropeptide FF	3.16
A5	HCRTR2	3062	S	peptide	Orexin A, orexin B	3.57

A5	LGR7	59350	S	peptide	Relaxin	3.78
A5	LGR8	122042	S	peptide	Relaxin	3.78
A5	LHCGR	3973	UD	peptide	gonadotropin	4.48
A5	NMBR	4829	UD	peptide	Neuromedin B	3.56
A5	NPFFR1	64106	S	peptide	Neuropeptide FF	3.16
A5	NPY2R	4887	UD	peptide	Neuropeptide Y	3.62
A5	NPY5R	4889	UD	peptide	Neuropeptide Y	3.62
A5	OXTR	5021	TD	peptide	Oxytocin	3.00
A5	PPYR1	5540	S	peptide	Pancreatic polypeptide	3.48
A5	TACR1	6869	UD	peptide	Substance P	3.05
A5	TACR3	6870	TD	peptide	Neurokinin A	3.05
A5	TSHR	7253	S	peptide	Thyroid-stimulating hormone	4.48
A5	GRPR	2925	WGD	peptide	Gastrin-releasing peptide	3.46
A6	GHSR	2693	S	peptide	Ghrelin	3.51
A6	GPR38	2862	S	peptide	Motilin	3.43
A6	NMUR1	10316	S	peptide	NMU, NMS	3.59
A6	NMUR2	56923	S	peptide	NMU, NMS	3.59
A6	NTSR1	4923	S	peptide	Neurotensin	3.19
A6	TRHR	7201	UD	peptide	Thyrotropin-releasing hormone	2.58
A7	AGTR1	185	S	peptide	Angiotensin	3.11
A7	AGTR2	186	S	peptide	Angiotensin	3.11
A7	AGTRL1	187	UD	peptide	Apelin	3.52
A7	BDKRB2	624	S	peptide	Bradykinin	3.03
A7	BLR1	643	UD	peptide	CXCL13	4.02
A7	C5R1	728	S	peptide	Anaphylatoxin C3a	3.90
A7	CCR10	2826	S	peptide	CCL27, CCL28	4.02
A7	CCR5	1234	S	peptide	CCR3,4,5,8,14	4.02
A7	CCR6	1235	S	peptide	CCL20	4.02
A7	CCR7	1236	S	peptide	CCL2, CCL7, CCL8, CCL13	4.02
A7	CCR9	10803	UD	peptide	CCL25	4.02
A7	CMKLR1	1240	UD	peptide	RARRES2	4.18
A7	CX3CR1	1524	S	peptide	CX3CL1	4.02
A7	CXCR3	2833	S	peptide	CXCL9,10,11,12	4.02
A7	CXCR4	7852	WGD	peptide	CXCL12	4.02

A7	FPR1	2357	S	peptide	N-Formyl-L-Met-L-Leu-L-Phe (fMLP)	2.60
A7	GALR1	2587	UD	peptide	Galanin	3.50
A7	GALR2	8811	S	peptide	Galanin	3.50
A7	GPR14	2837	UD	peptide	Urotensin II	3.14
A7	GPR145	2847	S	peptide	Melanin-concentrating hormone	3.38
A7	GPR24	84539	S	peptide	Melanin-concentrating hormone	3.38
A7	GPR30	2852	S	lipophylic molecule	progesterone	2.50
A7	GPR44	11251	S	lipophylic molecule	11-Dehydrothromboxane B2	2.56
A7	GPR54	84634	S	peptide	KiSS-1 gene product	3.77
A7	GPR8	2832	S	peptide	Neuropeptide W, neuropeptide B	3.49
A7	IL8RA	3577	S	peptide	CXCL8	4.02
A7	IL8RB	3579	S	peptide	CXCL1—3, CXCL5—8	4.02
A7	LTB4R	1241	UD	lipophylic molecule	Leukotriene B4	2.53
A7	OPRD1	4985	S	peptide	POMC-BETA-ENDORPHIN	3.26
A7	OPRK1	4986	UD	peptide	Dynorphin A	3.33
A7	OPRL1	4987	S	peptide	Nociceptin/orphanin FQ	3.26
A7	OPRM1	4988	S	peptide	Endorphin	3.04
A7	RLN3R1	51289	WGD	peptide	Relaxin-3	3.78
A7	SSTR1	6751	ND	peptide	Somatostatin	3.21
A7	SSTR2	6752	UD	peptide	Somatostatin	3.21
A7	SSTR3	6753	UD	peptide	Somatostatin	3.21
A7	SSTR5	6755	S	peptide	Somatostatin	3.21
A7	RLN3R2	339403	WGD	peptide	RLN3, INSL5	3.78
A7	XCR1	2829	UD	peptide	XCL1, CXCL2	4.02
B	ADCYAP1R1	117	UD	peptide	PACAP	3.50
B	CR	799	UD	peptide	Calcitonin, Amylin	3.54
B	CLR	10203	WGD	peptide	CGRP, adrenomedullin, intermedin	3.78
B	CRHR1	1394	S	peptide	urocortins	3.68
B	CRHR2	1395	ND	peptide	Urocortins	3.68
B	GCGR	2642	UD	peptide	Glucagon	3.58
B	GHRHR	2692	S	peptide	GHRH	3.71
B	GLP1R	2740	S	peptide	Glucagon-like peptide 1	3.58
B	GLP2R	9340	S	peptide	Glucagon-like peptide 2	3.58
B	PTHR1	5745	UD	peptide	Parathyroid hormone	3.50

B	PTHR2	5746	S	peptide	TIP-39	3.50
B	VIPR1	7433	WGD	peptide	VIP, PACAP	3.66
B	VIPR2	7434	UD	peptide	VIP, PACAP	3.66
C	CASR	846	S	ion	Calcium	1.60
C	GABBR1	2550	S	amine	GABAa	2.45
C	GPR51	9568	S	amine	GABAa	2.45
C	GPRC6A	222545	ND	amine	amino acids	2.04
C	GRM1	2911	WGD	amine	L-glutamate	2.16
C	GRM2	2912	TD	amine	L-glutamate	2.16
C	GRM3	2913	S	amine	L-glutamate	2.16
C	GRM4	2914	S	amine	L-glutamate	2.16
C	GRM5	2915	S	amine	L-glutamate	2.16
C	GRM6	2916	S	amine	L-glutamate	2.16
C	GRM7	2917	WGD	amine	L-glutamate	2.16
C	GRM8	2918	UD	amine	L-glutamate	2.16
F	FZD1	8321	S	peptide	extent Wnt2	4.56
F	FZD10	11211	S	peptide	Wnt	4.56
F	FZD3	7976	UD	peptide	Wnt	4.56
F	FZD4	8322	S	peptide	Wnt, Wnt5a, and NDP	4.56
F	FZD5	7855	S	peptide	Wnt and Wnt5a	4.56
F	FZD6	8323	S	peptide	Wnt	4.56
F	FZD7	8324	WGD	peptide	Wnt and Wnt5a	4.56
F	FZD8	8325	UD	peptide	Wnt and Wnt1	4.56
F	FZD9	8326	S	peptide	Wnt and Wnt2	4.56
F	SMO	6608	S	peptide	Wnt	4.56
