# Supplementary material for: GPCR Genes Are Preferentially Retained after Whole Genome Duplication
Source: PLoS One. 2008 Apr 2;3(4):e1903. doi: 10.1371/journal.pone.0001903 (PMC2270905; doi:10.1371/journal.pone.0001903)
Supplement: Table S5 — A. Inventory of polypeptide ligand genes in the MRCA of tetrapods and teleosts, human, and T. nigroviridis. B. List of polypeptide ligand genes in the MRCA of tetrapods and teleosts, and the derived ligand genes in human and T. nigroviridis. The accession number of identified ligands is listed. In cases where no existing accession number is available, the chromosomal position of the identified gene is provided. WGD, whole genome duplication; UD, undetermined; S, singleton. C. List of polypeptide ligands with WGD-derived duplicates in T. nigroviridis as well as their cognate receptors. Cognate receptors with WGD-derived duplicates in T. nigroviridis are shown in bold letters. The accession numbers for identified ligands are listed. In cases where no existing accession number is available, the chromosomal position of the identified gene is provided. (0.04 MB PDF) [file pone.0001903.s007.pdf]

Table S5 A. Inventory of polypeptide ligand genes in the MRCA of tetrapods and teleosts, human, and *T. nigroviridis*. Based on syntenic mapping, the 118 *T.nigroviridis* ligand genes are sub-divided as WGD-derived (WGD), singleton (S), undetermined (UD), and teleost-specific (T).

	MRCA		Human	*Tetraodon nigroviridis*					
Receptor class	No. of nGPCRs with a polypeptide ligand	No. of ligand genes	No. of human ligand genes	Total No. of ligand genes	WGD	UD	S	T	Ortholog found in *D. rerio* or *T. rubripes*
A5	26	31	37	41	8	8	23	2	0
A6	6	6	6	6	0	0	6	0	0
A7	36	24	57	46	4	19	14	8	1
B	13	15	18	25	6	10	9	0	0
Total	81	76	118	118	18	37	52	10	1

Table S5 B. List of polypeptide ligand genes in the MRCA of tetrapods and teleosts, and the derived ligand genes in human and *T. nigroviridis*. Whole genome duplication (WGD); unknown duplication(UD); singleton(S).

Ancestral count	Receptor Class	Ancestral ligand	Human ligand gene ID	*T.nigroviridis*				
				Ligand gene identifier	Origin of homologs	WGD	UD	S
1	A5	**AVP** - vasopressin	551	CAG09578	**S**	0	0	1
2	A5	**OXT -** oxytocin-neurophysin	5020	CAG09583	**S**	0	0	1
3	A5	**CCK** - cholecystokinin	885	CAF99479	**WGD**	1	0	0
				CAG12884		1	0	0
4	A5	**CGα** - glycoprotein hormones, alpha subunit	1081	CAG10482	**S**	0	0	1
5	A5	**EDN1** - endothelin 1	1906	chrUn_rnd:105180240-344	**UD**	0	1	0
				chrUn_rnd:125929085-177		0	1	0
6	A5	**EDN2** - endothelin 2	1907	CAG06007	**S**	0	0	1
7	A5	**EDN3** - endothelin 3	1908	CAG07564	**S**	0	0	1
8	A5	**FSHβ** - follicle-stimulating hormone	2488	CAF99923	**S**	0	0	1
9	A5	**GAST** - gastrin	2520	BAC44896	**S**	0	0	1
10	A5	**GNRH1** - gonadotrophin-releasing hormone 1	2796	BAE45689	**S**	0	0	1
11	A5	**GNRH2** - gonadotrophin-releasing hormone 2	2797	BAE45691	**S**	0	0	1
12	A5	**GPBβ5** - glycoprotein hormone beta 5	122876	CAG03758	**UD**	0	1	0
				CAG05739		0	1	0
13	A5	**GRP** - gastrin-releasing peptide	2922	chr4:3430570-1166	**S**	0	0	1
14	A5	**HCRT** - orexin A and orexin B	3060	CAG02825	**S**	0	0	1
15	A5	**INSL5** - insulin-like 5	10022	chr1:15664564-5453	**WGD**	1	0	0
				chr15:3444219-81		1	0	0
16	A5	**RLN3** - relaxin 3**, INSL3**	117579/3640	CAG06046	**WGD**	1	0	0
				chr3:8563693-833		1	0	0
17	A5	**RLN/RLN2/INSL4/INSL6**	6013/6019/3641/11172	chr4:1081257-741	**S**	0	0	1
18	A5	**LHβ** - luteinizing hormone, β subunit	3972	CAG00338	**S**	0	0	1
19	A5	**NMB** - neuromedin B	4828	chr5:2458757-852	**WGD**	1	0	0
				chr13:2188886-963		1	0	0
20	A5	**NPFF, NPAF**	8620	CAG07075	**S**	0	0	1
21	A5	**NPY** - neuropeptide Y	4852	CAG11987	**UD**	0	1	0
				CAG11107		0	1	0
22	A5	**PYY, PPY**	5697/5539	CAG02076	**UD**	0	1	0
				chr2:4330403-1166		0	1	0
23	A5	**P518** - RF-Amide P518	347148	GSCT00026421001	**S**	0	0	1
24	A5	**PrRP** - prolactin-releasing peptide	51052	chr6:1313515-86	**S**	0	0	1

Ancestral count	Receptor Class	Ancestral ligand	Human ligand gene ID	*T.nigroviridis* Ligand gene identifier	Origin of homologs	WGD	UD	S
**25**	A5	**PROK1** - prokineticin 1 (EG-VEGF)	84432	CAG07489	**S**	0	0	1
**26**	A5	**PROK2** - prokineticin 2	60675	CAF96920	**S**	0	0	1
**27**	A5	**RFRP** - RFamide-related peptide	64111	CAF99426	**S**	0	0	1
**28**	A5	**TAC1** - substance P/tachykinin1	6863	CAG11662	**S**	0	0	1
**29**	A5	**TAC3** - neurokinin B /tachikinin 3	6866	CAG07431	**S**	0	0	1
**30**	A5	**TAC4** - tachykinin 4	255061	CAG04412	**S**	0	0	1
**31**	A5	**TSHβ** - thyroid-stimulating hormone	7252	CAG11806	**S**	0	0	1
**Subtotal**			**36**			**8**	**8**	**23**
**1**	A6	**GHRL** - ghrelin	51738	chr16:8282093-8282194	**S**	0	0	1
**2**	A6	**MLN** - motilin	4295	chr11:774453-775188	**S**	0	0	1
**3**	A6	**NMS** - neuromedin S	129521	chr2:17978277-659	**S**	0	0	1
**4**	A6	**NMU** - neuromedin U	10874	chr1_rnd:2147854-9206	**S**	0	0	1
**5**	A6	**NTS** - neuromedin N and neurotensin	4922	chr13:11758087-185	**S**	0	0	1
**6**	A6	**TRH** - thyrotropin-releasing hormone	7200	CAG11330	**S**	0	0	1
**Subtotal**			**6**			**0**	**0**	**6**
**1**	A7	**AGT** - angiotensin	183	CAG10094	**S**	0	0	1
**2**	A7	**APLN** - apelin	8862	CAG08364	**S**	0	0	1
**3**	A7	**CORT** - cortistatin	1325	CAG07556	**S**	0	0	1
**4**	A7	**GAL** - galanin	51083	chrUn_rnd:85620427-1348	**S**	0	0	1
**5**	A7	**KISS1**	3814	SCAF14526	**S**	0	0	1
**6**	A7	**KNG1** - bradykinin	3827	CAF96097	**S**	0	0	1
**7**	A7	**NPB** - neuropeptide B	256933	CAG00214	**WGD**	1	0	0
				CAG06031		1	0	0
**8**	A7	**PDYN** - dynorphin A	5173	*D.rerio*	**S**	0	0	1
**9**	A7	**PENK** - proenkephalin	5179	CAG11635	**S**	0	0	1
**10**	A7	**POMC -** proopiomelanocortin	5443	Un_rnd:112416896-112417622	**UD**	0	1	0
				14:4949877-4950632		0	1	0
**11**	A7	**MCH** - melanin-concentrating hormone	5367	CAF93560	**UD**	0	1	0
				chr19:990239-877		0	1	0
**12**	A7	**NOC** - nociceptin/orphanin FQ	5368	CAG12991	**UD**	0	1	0
				CAG03094		0	1	0
				chrUn_rnd:79082359-535		0	1	0
**13**	A7	**SST** - somatostatin	6750	CAG07675	**UD**	0	1	0
				CAG02176		0	1	0
**14**	A7	**UTS2** - urotensin II	10911	CAG04941	**S**	0	0	1
**15**	A7	**UTS2D -** urotensin related peptide	257313	chr9:8502656-967	**S**	0	0	1
**16**	A7	**CCL20**	6364	chr15:4409609-10309	**S**	0	0	1

Ancestral count	Receptor Class	Ancestral ligand	Human ligand gene ID	*T.nigroviridis*				
				**Ligand gene identifier**	**Origin of homologs**	**WGD**	**UD**	**S**
**17**	A7	**CCL22**	6367	SCAF2108	**S**	0	0	1
**18**	A7	**CCL24**	6369	chr18:7004875-6283	**S**	0	0	1
**19**	A7	**CCL21/25**	6366/6370	chr15:5048784-9029	**UD**	0	1	0
				CAG07167	**WGD**	1	0	0
				CAG07971		1	0	0
**20**	A7	**CCL27/28**	10850/56477	chr15:5046891-5047156	**UD**	0	1	0
				chr15:4114798-956	**UD**	0	1	0
				CAF90799	**UD**	0	1	0
				chr2:17691812-907	**UD**	0	1	0
				CAG09577	**UD**	0	1	0
**21**	A7	**CXCL8**	3576	CAG11181	**UD**	0	1	0
				CAF90539	**UD**	0	1	0
**22**	A7	**CXCL9/10/11**	4283/3627/6373	GSCT00006672001	**S**	0	0	1
**23**	A7	**CXCL12**	6387	CAG09382	**UD**	0	1	0
				chr18:2280936-96670	**UD**	0	1	0
**24**	A7	**CXCL14**	9547	CAF90863	**S**	0	0	1
**Subtotal**			**28**			**4**	**19**	**15**
**1**	B	**ADM** - adrenomedullin	133	CAF99639	**WGD**	1	0	0
				CAG05606		1	0	0
				chr15:7151047-646	**UD**	0	1	0
**2**	B	**IMD** - intermedin	79924	chr13:3271568-697	**UD**	0	1	0
				CAG13555		0	1	0
**3**	B	**CALCA, CALCB-** calcitonin; calcitonin gene-related peptide	796/797	CAF99659	**WGD**	1	0	0
				chr5: 6718835-9962		1	0	0
				chrUn_rnd:66411282-990	**UD**	0	1	0
**4**	B	**IAPP** - amylin	3375	CAF89835	**S**	0	0	1
**5**	B	**CRH** - corticotropin releasing factor	1392	chr6:2514921-5169	**S**	0	0	1
**6**	B	**GCG** - glucagon, GLP1, GLP2	2641	CAG04974	**WGD**	1	0	0
				CAG09338 (AA740-end)		1	0	0
**7**	B	**GHRH** - growth hormone-releasing hormone	2691	CAF96902	**S**	0	0	1
**8**	B	**ADCYAP1 -** adenylate cyclase activating polypeptide 1	116	CAG12289	**UD**	0	1	0
				GSTENT00031777001		0	1	0
				CAG10213		0	1	0
**9**	B	**PTH** parathyroid hormone	5741	chrUn_rnd:153387022-120	**S**	0	0	1
**10**	B	**PTHLH** - parathyroid hormone-like hormone	5744	CAG08679	**UD**	0	1	0
				CAF88940=CAF87848		0	1	0
				chrUn_rnd:12100351-452		0	1	0
**11**	B	**TIP39**	113091	CAG12455	**S**	0	0	1
**12**	B	**UCN** - urocortin	7349	chr14:9250391-492	**S**	0	0	1
**13**	B	**UCN2** - urocortin 2	90226	chr11:1019834-998	**S**	0	0	1
**14**	B	**UCN3** - urocortin 3	114131	CAG07278	**S**	0	0	1
**15**	B	**VIP -** vasoactive intestinal polypeptide	7432	CAF97050	**S**	0	0	1
**Subtotal**			**16**			**6**	**10**	**9**
**Ligand genes not present in *T. nigroviridis***								
1	A5	**NPS** - neuropeptide S	594857					

Ancestral count	Receptor Class	Ancestral ligand	Human ligand gene ID	*T.nigroviridis*				
				Ligand gene identifier	Origin of homologs	WGD	UD	S
**2**	A7	**GALP** - galanin-like peptide	85569					
**3**	A7	**NPW** - neuropeptide W	283869					
**4**	A7	**CCL1**	6346					
**5**	A7	**CCL2**	6347					
**6**	A7	**CCL3**	6348					
**7**	A7	**CCL4**	6351					
**8**	A7	**CCL5**	6352					
**9**	A7	**CCL7**	6354					
**10**	A7	**CCL8**	6355					
**11**	A7	**CCL11**	6356					
**12**	A7	**CCL13**	6357					
**13**	A7	**CCL14**	6358					
**14**	A7	**CCL15**	6359					
**15**	A7	**CCL16**	6360					
**16**	A7	**CCL17**	6361					
**17**	A7	**CCL18**	6362					
**18**	A7	**CCL19**	6363					
**19**	A7	**CCL23**	6368					
**20**	A7	**CCL26**	10344					
**21**	A7	**CXCL1**	2919					
**22**	A7	**CXCL2**	2920					
**23**	A7	**CXCL3**	2921					
**24**	A7	**CXCL4=PF4V**	5197					
**25**	A7	**CXCL5**	6374					
**26**	A7	**CXCL6**	6372					
**27**	A7	**CXCL7=PPBP**	5473					
**28**	A7	**CXCL13**	10563					
**29**	A7	**CXCL15**	337989					
**30**	A7	**CXCL16**	58191					
**31**	B	**SCT** - secretin	6343					
**32**	B	**GIP -** gastric inhibitory polypeptide	2695					

Teleost-specific genes

**1**	A5	**EDN-like**	1000001	chr8:5583520-870				
**2**	A5	**GNRH-like**	1000002	BAE45693				
**1**	A7	**CCL-like**	1000003	CAG07793				
**2**				CAG07792				
**3**				chr2:17775947-17776174				
**4**				CAF98923				
**5**				chr12:3595953-6120				
**6**				chr12:3597953-8132				
**7**	A7	**CXCL-like**	1000004	CAF95728				
**8**				CAG09635				

Table S5 C. List of polypeptide ligands with WGD-derived duplicates in *T. nigroviridis* as well as their cognate receptors. Cognate receptors with WGD-derived duplicates in *T. nigroviridis* are shown in bold letters.

	Receptor class	Ligand gene symbol	Ligand description	Cognate receptors	Human ligand gene ID	*T. nigroviridis* ligand gene identifier
Coevolved with a pair of WGD-derived nGPCRs	A5	***NMB***	Neuromedin B	***GRPR***, *NMBR*	4828	chr5:2458757-852<br>chr13:2188886-963
	A5	***INSL5***	Insulin-like 5	***RLN3R2***	10022	chr1:15664564-5453<br>chr15:3444219-81
	A7	***RLN3***	Relaxin-3	***RLN3R1***, *LGR7*	117579	CAG06046<br>chr3:8563693-833
	B	***ADM***	Adrenomedullin	***CRLR***	133	CAF99639<br>CAG05606
	B	***CALCA***	Calcitonin; calcitonin gene-related peptide	***CRLR***, *CR*	796	CAF99659<br>chr5: 6718835-9962
Not coevolved with a pair of WGD-derived nGPCRs	A5	***CCK***	Cholecystokinin	*CCKAR*	885	CAF99479<br>CAG12884
	A7	***NPB***	Neuropeptide B	*GPR7, GPR8*	256933	CAG00214<br>CAG06031
	A7	***CCL25***	CCL25	*CCR9*	6370	CAG07167<br>CAG07971
	B	***GCG***	Glucagon, GLP1, GLP2	*GCGR, GLP1R, GLP2R*	2641	CAG04974<br>CAG09338 (amino acid 740-end)
